# Prediction of outcome in newly diagnosed myeloma: a meta-analysis of the molecular profiles of 1905 trial patients

**DOI:** 10.1038/leu.2017.179

**Published:** 2017-06-30

**Authors:** V Shah, A L Sherborne, B A Walker, D C Johnson, E M Boyle, S Ellis, D B Begum, P Z Proszek, J R Jones, C Pawlyn, S Savola, M W Jenner, M T Drayson, R G Owen, R S Houlston, D A Cairns, W M Gregory, G Cook, F E Davies, G H Jackson, G J Morgan, M F Kaiser

**Affiliations:** 1 Division of Molecular Pathology, The Institute of Cancer Research, London, UK; 2 MIRT, University of Arkansas for Medical Sciences, Little Rock, AR, USA; 3 Department of Haematology, Hopital Huriez, CHRU, Lille, France; 4 MRC Holland, Amsterdam, The Netherlands; 5 Department of Haematology, University Hospital Southampton, Southampton, UK; 6 Clinical Immunology, School of Immunity & Infection, University of Birmingham, Birmingham, UK; 7 Department of Haematology, St James’s University Hospital, Leeds, UK; 8 Clinical Trials Research Unit, University of Leeds, Leeds, UK; 9 Section of Experimental Haematology, Leeds Institute of Cancer & Pathology, University of Leeds, Leeds, UK; 10 Department of Haematology, Newcastle University, Newcastle upon Tyne, UK

## Abstract

Robust establishment of survival in multiple myeloma (MM) and its relationship to recurrent genetic aberrations is required as outcomes are variable despite apparent similar staging. We assayed copy number alterations (CNA) and translocations in 1036 patients from the NCRI Myeloma XI trial and linked these to overall survival (OS) and progression-free survival. Through a meta-anlysis of these data with data from MRC Myeloma IX trial, totalling 1905 newly diagnosed MM patients (NDMM), we confirm the association of t(4;14), t(14;16), t(14;20), del(17p) and gain(1q21) with poor prognosis with hazard ratios (HRs) for OS of 1.60 (*P*=4.77 × 10^−7^), 1.74 (*P*=0.0005), 1.90 (*P*=0.0089), 2.10 (*P*=8.86 × 10^−14^) and 1.68 (*P*=2.18 × 10^−14^), respectively. Patients with ‘double-hit’ defined by co-occurrence of at least two adverse lesions have an especially poor prognosis with HRs for OS of 2.67 (*P*=8.13 × 10^−27^) for all patients and 3.19 (*P*=1.23 × 10^−18^) for intensively treated patients. Using comprehensive CNA and translocation profiling in Myeloma XI we also demonstrate a strong association between t(4;14) and *BIRC2/BIRC3* deletion (*P*=8.7 × 10^−15^), including homozygous deletion. Finally, we define distinct sub-groups of hyperdiploid MM, with either gain(1q21) and *CCND2* overexpression (*P*<0.0001) or gain(11q25) and *CCND1* overexpression (*P*<0.0001). Profiling multiple genetic lesions can identify MM patients likely to relapse early allowing stratification of treatment.

## Introduction

While survival for multiple myeloma (MM) has improved over the last decade with the introduction of immunomodulatory drugs and proteasome inhibitors most MM patients will still relapse.^1^ Upfront identification of patients who are likely to relapse early offers the prospect of intervening pre-emptively to maintain remission. Furthermore, identifying tumor sub-groups with targetable molecular dependencies has the potential to inform on biologically driven therapy.

Myeloma cells are typified by recurrent chromosomal aberrations, a number of which have been variously associated with poor prognosis, notably t(4;14), t(14;16), t(14;20), deletion 17p and gain of 1q.^2^ We and others have recently reported that the co-occurrence of multiple genetic lesions may have greater significance for predicting patient outcome than any single abnormality.^3, 4^ Since many of the molecular abnormalities in MM are only present at relatively low frequency, robustly establishing the impact of molecular sub-classes on prognosis is contingent on the analysis of large patient series that have been uniformly treated.

Here we report a meta-analysis of the relationship between genetic profile and prognosis in newly diagnosed MM (NDMM) using data from two UK multi-center phase III clinical trials, totalling 1905 patients. This dataset includes previously generated data on the MRC Myeloma IX trial and an expanded analysis of the NCRI Myeloma XI trial. In addition, we analysed molecular copy number profiling in 1036 Myeloma XI patients to identify sub-groups with molecular addictions that could be therapeutically targetable.^5, 6^

## Materials and methods

### Myeloma XI trial patients

1036 patients with NDMM enrolled in the UK NCRI Myeloma XI phase III trial were molecularly profiled. Trial characteristics are described in Supplementary Methods. At the time of analysis, the trial endpoints have not been published. Median follow-up was 36.0 months. The study was undertaken with written informed consent from patients and ethical approval was obtained from the Oxfordshire Research Ethics Committee (MREC 17/09/09, ISRCTN49407852).

### Myeloma IX trial patients

Detailed characteristics and main outcomes of MRC Myeloma IX have been reported previously and summarised in Supplementary Methods.^7^ The study was undertaken with written informed consent from patients and ethical approval was obtained from the MRC Leukaemia Data Monitoring and Ethics committee (MREC 02/08/95, ISRCTN68454111). For the present analysis we included data from 869 of the 1960 NDMM patients with available clinical and comprehensive cytogenetic data.^3^ Median follow-up for this group was 72 months.^3, 8^ Accompanying gene expression and mapping array data have been previously published (GSE15695).^6, 9, 10^

### Samples

For both trials myeloma cells from bone marrow aspirate samples were obtained at diagnosis and purified (>95%) using immune-magnetic cell sorting (Miltenyi Biotec, Bergisch Gladbach, Germany). RNA and DNA were extracted using RNA/DNA mini kit or Allprep kits (QIAGEN) according to manufacturers’ instructions.

### Copy number and translocation detection

Technical details about fluorescence *in situ* profiling of Myeloma IX have been published previously.^11^ Myeloma XI cases were centrally analysed using MLPA and qRT-PCR. The SALSA MLPA P425-B1 MM probemix (MRC Holland, Amsterdam, The Netherlands) was used as previously described.^12, 13^ The newly developed probemix X073-A1 was used to profile 1007 of the 1036 cases in an identical fashion (MLPA Probe Mix: Supplementary Table 1). Copy number at each locus was determined as described previously.^12, 13^

Multiplexed qRT-PCR was used to determine *IGH* translocation status using a translocation and cyclin D (TC)-classification based algorithm (Supplementary Methods), as previously described.^10^

### Statistical methods

All statistical analyses were undertaken using R version 3.3 and the ‘survival’, ‘rms’, ‘metafor’, ‘survC1’, ‘JAGS’ and ‘BayesMed’ packages.^14^

Progression-free survival (PFS) was defined as the time from the date of randomization to progression, according to IMWG criteria, or death from any cause. Overall survival (OS) was defined as the time from the date of randomization to death from any cause. Kaplan–Meier survival curves were generated and the homogeneity between groups was evaluated with the log-rank test. Cox regression analysis was used to estimate hazard ratios (HRs) and respective 95% confidence intervals (CI) and adjustment for variables was performed by multivariable analysis. Fixed effects meta-analysis was performed using individual patient data. Correlations between structural aberrations were analysed using Bayesian inference. A Bayes factor (BF_01_) of BF_01_<0.01 was considered significant. The association between categorical variables was examined using the Fisher exact test. The association between myeloma subtype and gene expression was assessed using the Mann–Whitney test. A two-sided *P*-value <0.05 was considered significant.

## Results

### Descriptive patient characteristics and structural aberrations

The clinical characteristics of the 1036 newly profiled Myeloma XI trial patients and the 869 Myeloma Trial IX patients are detailed in Table 1. Overall there were no significant differences between trial patients in terms of gender, age and proportion that had been in receipt of intensive/non-intensive therapy. Although the frequencies of the primary IGH translocations, del(17p), del(1p32), del(13q) and del(16q) in tumours were similar in Myeloma IX and XI trial patients, a higher proportion of Myeloma IX patients had hyperdiploidy (HRD), gain(1q) and del(22q) (Table 1). Amongst Myeloma XI trial patients, homozygous deletion of *CDKN2C* (1p32), *BIRC2/BIRC3* (11q22) and amplification of *CKS1B* (1q21) and *MYC* (8q24) were the commonest focal homozygous copy number changes, which were seen at similar frequencies to those previously reported (Table 1).^15^

### Relationship between cytogenetic aberrations and survival

In both trial series, the archetypical high-risk lesions del(17p), gain(1q) and t(4;14) were each significantly associated with shorter PFS and OS (Table 2). In the combined analysis, respective HR for OS were 2.1 for del(17p) (*P*=8.86 × 10^−14^), 1.68 for gain(1q) (*P*=2.18 × 10^−14^) and 1.60 for t(4;14) (*P*=4.77 × 10^−7^; Table 2; Supplementary Figures 1 and 2). In addition, the t(14;16) and t(14;20) translocations involving *MAF* and *MAFB* were also associated with shorter OS with respective HRs of 1.74 (*P*=0.0005) and HR 1.90 (*P*=0.0089). Respective inference C-statistic estimates for adequacy of risk prediction are shown in Supplementary Tables 5 and 6.

Deletion of 1p32 (*CDKN2C*) was significantly associated with shorter OS (HR 1.46; *P*=0.0002; Table 2). This association was confined to patients in receipt of intensive treatment (in the combined analysis: HR 1.89; *P*=1.23 × 10^−5^ vs HR 1.05; *P*=0.72 for non-intensive treatment). The association of del(1p32) with OS was independent from gain(1q21) by multivariable analysis (*P*<0.05) in the intensive treatment groups of both trials.

**Figure 1:**
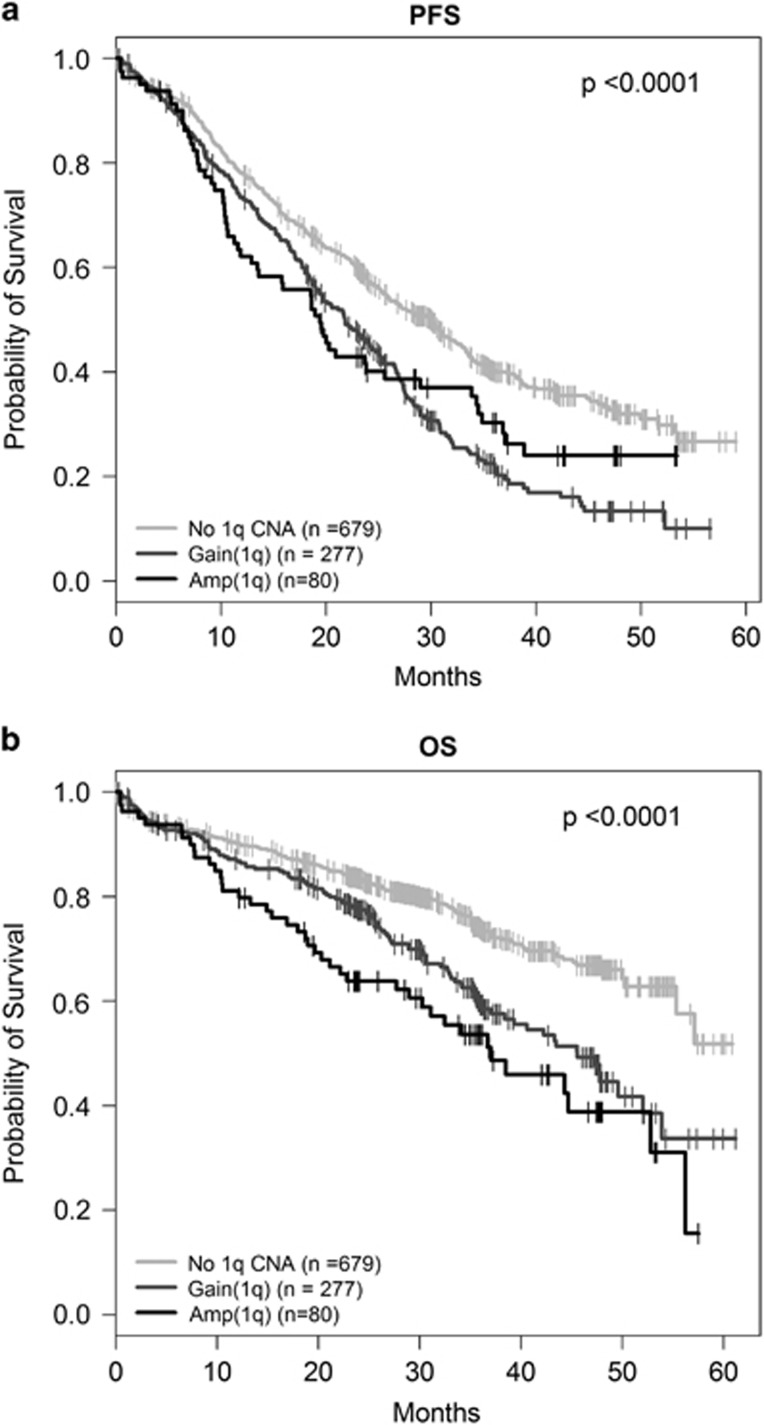
Chromosome 1q21 copy number status and outcome in Myeloma XI. Kaplan–Meier curves and log-rank *P*-values for (**a**) PFS (**b**) OS for normal vs gain vs amplification of 1q21.

To examine the relationship between 1q21 status and outcome in more detail we sub-classified Myeloma XI patients (*n*=1036) by diploid vs gain vs amplification status. 1q21 gain was confirmed as a high-risk lesion and was associated with significantly shorter PFS (HR 1.56; *P*=3.53 × 10^−7^) and OS (HR 1.67; *P*=3.30 × 10^−5^) than normal 1q copy number status. Amp(1q) was also associated with shorter PFS (HR 1.44; *P*=0.01) and OS (HR 2.28; *P*=2.32 × 10^−6^) compared to normal 1q, but there was no significant difference to gain(1q) (PFS: HR 0.91; *P*=0.54; OS: HR 1.36; *P=*0.09 for OS). Median PFS was 19.4 vs 21.8 vs 30.1 months (*P*<0.0001) and 24-months OS 63.8 vs 77.5 vs 83.5% (*P*<0.0001) for amp(1q), gain(1q) and normal 1q, respectively (Figure 1; Supplementary Table 7).

### ‘Double-hit’ as a high-risk classifier

We next examined the impact of a ‘double-hit’ based on the co-occurrence of at least any two of the following: (1) Adverse translocations t(4;14), t(14;16), t(14;20); (2) gain(1q); (3) del(17p).^3^ For Myeloma XI the three risk groups, defined by ‘double-hit’, 1 or no adverse lesions, were associated with median PFS of 17.0, 24.2 and 31.1 months (log-rank *P*=5.7 × 10^−13^), with corresponding median 24-months OS of 66.1, 76.6 and 86.4% (*P*=4.4 × 10^−13^). These findings were consistent with Myeloma IX (Table 2). In the combined analysis of all 1905 patients the HR for ‘double-hit’ was 2.23 for PFS (*P*=7.92 × 10^−26^) and 2.67 for OS (*P*=8.13 × 10^−27^; Table 2; Supplementary Figures 1 and 2). Similarly to Myeloma IX, the ‘triple-hit’ of an adverse translocation, Gain(1q) and del(17p) was associated with a very short median OS of 19 months with a HR of 6.23 (*P*=1.31 × 10^−7^) vs no adverse lesion (Supplementary Figure 5).

In both Myeloma IX and XI trials the impact of a ‘double-hit’ on patient outcome was independent of International Staging System (ISS; Supplementary Table 3). Moreover, integration of ISS and genetic risk defined ‘double-hit’-ISS ultra high risk (ISS II or III and ‘double-hit’ 12.0%), intermediate risk (ISS I and ‘double-hit’ ISS II and 1 adverse lesion; ISS III and no or 1 adverse lesion; 44.1%) and favourable risk groups (ISS I and no or 1 adverse lesion; ISS II and no adverse lesion; 43.9%). 'double hit'-ISS ultra high risk was associated with HR 2.85 (*P*=8.32 × 10^−31^) for PFS and HR 4.12 (*P*=2.85 × 10^−36^) for OS in the meta-analysis (Table 2).

### Genetic markers and survival in intensively treated patients

Since young and fit patients are most likely to be considered for intensified combination therapy, we subsequently focused on the relationship between molecular profile and survival of this sub-group of Myeloma XI (*n*=598) and Myeloma IX (*n*=511) patients.

In these 1109 intensively treated patients, del(17p), gain(1q) and t(4;14) were consistently associated with shorter PFS and OS; combined HRs of 2.65 (3.04 × 10^−12^), 1.77 (1.65 × 10^−8^) and 1.87 (7.62 × 10^−7^), respectively (Table 3; Supplementary Figures 3 and 4). In this group, t(14;16) was associated with shorter PFS (HR 1.80; *P*=0.0021) and OS (HR 1.82; *P*=0.013). The t(14;20) was not associated with adverse PFS or OS, but the lesion was only present in eight Myeloma IX and five Myeloma XI cases (Table 3; Supplementary Figures 3 and 4).

In Myeloma XI, the groups with a ‘double-hit’, 1 adverse or no adverse lesion were associated with median PFS of 19.7, 30.9 and 44.8 months (log-rank *P*=2.5 × 10^−13^) and 24-months OS of 72.3, 86.2 and 92.2% (*P*=1.6 × 10^−10^; Supplementary Figure 3). By meta-analysis, intensively treated patients with a ‘double-hit’ had a HR for PFS of 2.61 (*P*=1.07 × 10^−20^) and HR for OS of 3.19 (*P*=1.23 × 10^−18^; Table 3). Survival time increased for all risk groups of intensively treated patients in Myeloma XI compared to Myeloma IX (median PFS: 14.4, 21.9 and 30.8 months; 24 month OS: 63.9, 75.4 and 86.0%, respectively). Median PFS was 5.3 months longer for ‘double-hit’ in Myeloma XI vs IX, but 14 months longer for the group without any risk lesion.

On the basis of clinical and genetic information (Supplementary Table 4) the ‘double-hit’-ISS ultra high-risk group comprising 12.5% of patients were associated with a HR of 3.11 (*P*=1.59 × 10^−20^) for PFS and HR 4.79 (*P*=5.10 × 10^−23^) for OS.

### Associations of copy number changes with translocations and targetable lesions

**Figure 2:**
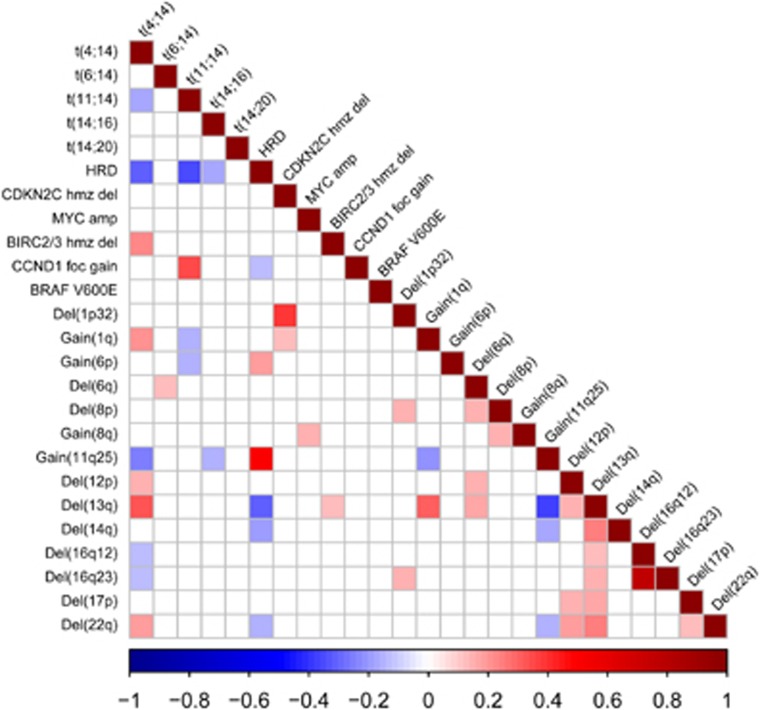
Associations between copy number aberrations and translocations in Myeloma XI. A Bayesian approach was used to identify all potential associations between genetic lesions. Significant interactions (BF<0.01) are colour-coded, red representing positive and blue negative associations. Correlation factors and Bayes Factors are provided in Supplementary Table 2. amp, amplification; foc gain, focal gain; hmz del, homozygous deletion.

We next focused on genetic sub-groups of MM that could be specifically targetable using copy number and translocation data on the 1036 Myeloma XI patients. Figure 2 provides an overview of correlations between CNA and translocations (Supplementary Table 2). Of particular note was a relationship between NFκB-pathway CNA and translocation groups.

### Potentially targetable NFκB-pathway gene deletions are common in myeloma

Deletions of NFκB-pathway modulating genes *TNFAIP3*, *BIRC2/BIRC3*, *TRAF3* or *CYLD* were identified in 16.6, 4.8, 13.9 and 16.9% of Myeloma XI cases, respectively. Nearly half of all tumors (43.2%) harbored an NFκB-pathway gene abnormality. Overall, a deletion of more than one NFκB-pathway gene was detectable in 9.7% of tumors and in 42.4% of these cases involved deletions of both *TNFAIP3* and *CYLD*.

### t(4;14) myeloma is associated with *BIRC2/BIRC3* deletions

We identified *BIRC* NFκB-pathway deletions to be enriched in t(4;14) MM (Figure 2). Specifically, 29/135 (21.5%) t(4;14) vs 20/852 (2.3%) non-t(4;14) (*P*=8.7 × 10^−15^) tumors were *BIRC2/BIRC3* deleted. Intriguingly, homozygous *BIRC2/BIRC3* deletions were present in 15/135 (11%) of t(4;14) as compared to 7/872 non-t(4;14) tumors (0.8% BF_01_=4.3 × 10^−12^; *P*=1.0 × 10^−8^; Supplementary Table 2). Almost all t(4;14) tumors (28/29) with any *BIRC2/BIRC3* deletion expressed *FGFR3*, more than expected (*P*=0.015). Deletions of *TRAF3* (14q32) seen in t(4;14) were mutually exclusive of *BIRC* deletions (*P*=0.016) and more than expected *FGFR3*-negative: 20/29 (69% *P*=0.0001). In contrast, deletions of *CYLD* were generally significantly under-represented in the t(4;14) group (6/137; 4.4%) as compared with non-t(4;14) (169/899; 18.8% BF=0.007; *P*=4.0 × 10^−6^).

### High-risk and hypodiploidy-associated lesions

The t(4;14) subgroup was significantly associated with hypodiploidy lesions (HYL) del(12p) (BF=1.1 × 10^−4^), del(13q) (BF=1.1 × 10^−25^) and del(22q) (BF=1.1 × 10^−7^; Figure 2).^16^ Deletion of 17p was also associated with deletions of 12p (BF=0.0004), 13q (BF=3.7 × 10^−6^) and 22q (BF=0.0076), but there was no correlation between del(17p) and t(4;14). MM with t(4;14) was associated with gain(1q) (BF=3.0 × 10^−8^), but not with del(1p32), which was only significantly correlated with del(8q) (BF=0.0009) and del(16q23) (BF=0.0003). In contrast, t(11;14) and HRD cases, the latter defined by extra copies of any two of chromosomes 5, 9 or 15, were negatively associated with gain(1q) (BF=1.6 × 10^−3^ and BF=0.06, respectively). Collectively, HRD cases were negatively associated with del(13q) (BF=1.9 × 10^−21^) and del(22q) (BF=0.0002).

### Molecular sub-classification of hyperdiploid myeloma

**Figure 3:**
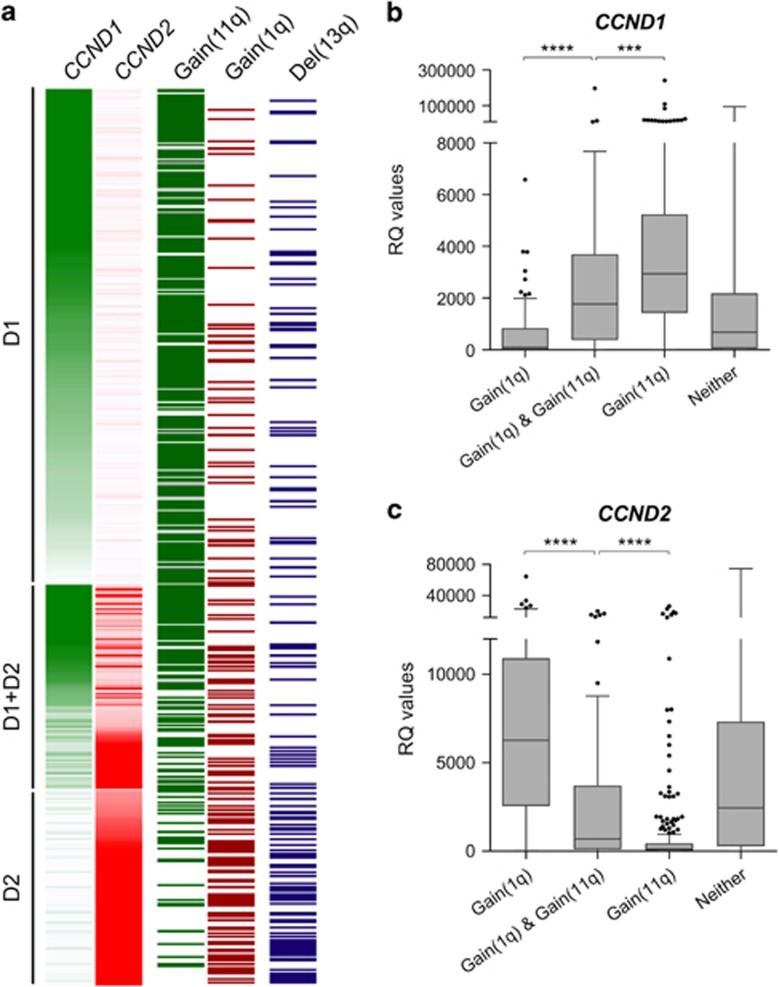
HRD genetic sub-groups in Myeloma XI. (**a**) Each row represents one of in total 1007 cases. Expression intensity is coded in green for *CCND1* and red for *CCND2* expression. Gain of 11q25 is shown in dark green, gain of 1q in dark red and deletion 13q in dark blue; white=no abnormality detected. B+C. *CCND1* (**b**) and CCND2 (**c**) qRT-PCR expression levels (relative quantitative RQ values, *GAPDH* normalised) for HRD cases with gain(1q), gain(1q)+gain(11q25), gain(11q25) or neither. Gene expression levels were significantly different for all possible group-wise comparisons (two-sided Mann–Whitney *U* test; ****P<0.0001; ***P<0.001).

We noted heterogeneity within the HRD subgroup in terms of co-occurrence of lesions. Although HRD as a whole group was strongly correlated with gain(11q25) (BF=1.2 × 10^−66^), a subgroup lacking gain(11q25) was characterised by gain(1q) (Figure 3; Supplementary Figure 6).

Of the 488 HRD cases in Myeloma XI, 68% had gain(11q25) and 29% gain(1q). Both lesions co-occurred in 15% of HRD cases, less than expected (BF=0.0004). Accordingly, most HRD patients could be classified as having gain(1q)-HRD, gain(11q25)-HRD or gain(1q)+gain(11q25)-HRD (Figure 2a). Gain(1q)-HRD was associated with overexpression of *CCND2* and silenced *CCND1* (*P*<0.0001). In contrast, gain(11q25) was associated with *CCND1* expression and silenced *CCND2* (*P*<0.0001). We validated this correlation between gain(1q)-HRD and *CCND2* and gain(11q25)-HRD and *CCND1* expression in the Myeloma IX dataset (Supplementary Figure 7). The TC classification-defined D1 and D2 sub-groups of HRD MM on the basis of *CCND1* and *CCND2* overexpression^17^ and our findings suggest similarity between gain(11q25)-HRD and the D1, gain(1q)-HRD and the D2 and gain(1q)+gain(11q)-HRD and the D1+D2 TC classification subgroup.

Further differences between the HRD subtypes were noted: 13q was deleted in 41.1% (58/141) of gain(1q)-HRD (BF=6.0 × 10^−6^; *P*<0.0001), but only in 15.4% (50/325) of gain(11q25)-HRD (BF=5.5 × 10^−11^; *P*<0.0001). We validated this finding in the Myeloma IX dataset, where del(13q) was also positively associated with gain(1q)-HRD (*P*=0.024) and negatively associated with gain(11q25) (*P*=0.041).

### Prognostic impact of molecular sub-groups in HRD

**Figure 4:**
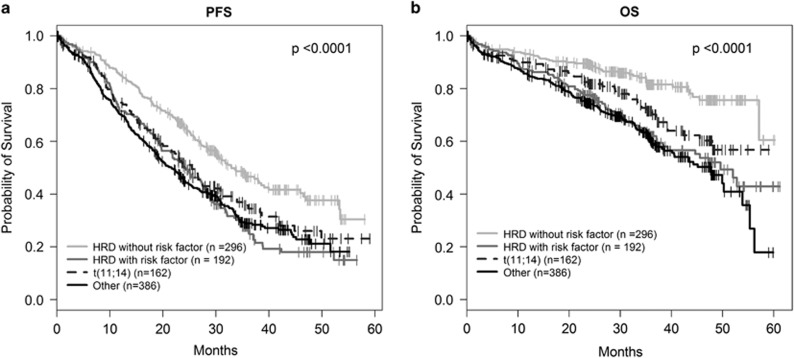
Survival in HRD MM with and without risk factors in Myeloma XI. Kaplan–Meier curves and log-rank *P*-values for (**a**) PFS (**b**) OS.

Gain of 1q, del(1p32) and del(17p) was associated with shorter OS (HR 1.81, *P*=0.001; HR 2.44, *P*=0.0004; HR 1.89, *P*=0.022; respectively) in the 488 HRD cases. Gain(1q) and del(1p32) but not del(17p) was also associated with shorter PFS (HR 1.56, *P*=0.0003; HR 1.66, *P*=0.005; HR 1.30, *P*=0.23 respectively; (Supplementary Table 8, Supplementary Figure 8)). Gain of 11q25, del(13q) and del(22q) were not associated with shorter OS or PFS. At least one of the lesions gain(1q), del(1p32) or del(17p) were present in 39.3% (192/488) of HRD cases, defining a risk population with significantly shorter PFS (*P*=4.9 × 10^−6^) and OS (*P*=2.7 × 10^−6^; Supplementary Figure 9) compared to HRD MM lacking any of these lesions. Interestingly, the 28.5% of all patients (296/1036) that had HRD without any demonstrable adverse lesion, had the longest survival of all sub-groups, indeed longer than those with t(11;14) MM (Figure 4).

## Discussion

Our analysis confirms the association with outcome in MM for the archetypical high-risk lesions del(17p), gain(1q) and adverse translocations and emphasises the importance of ‘double-hit’ as a risk biomarker. Importantly, we demonstrate that this information can be combined with the ISS to further refine risk prediction.^3, 18, 19, 20, 21^ To our knowledge, this study represents the largest analysis investigating the additive effect of multiple genetic lesions on outcome in NDMM. Importantly, our analysis has been based on trials that recruited between 2003 and 2016, a timeframe during which treatment for MM has undergone significant change.^22^ The consistent adverse impact of high-risk genetics on survival in Myeloma IX and XI is striking and highlights the need for intensified efforts to target the biology of high-risk disease. Although survival time increased for all risk groups in Myeloma XI vs IX, absolute improvement was smallest for the ‘double-hit’ high-risk group. Median PFS for ‘double-hit’ in Myeloma XI patients receiving intensive treatment was 19.7 months, meaning that about half of patients relapsed 12 months following autologous transplant.

Comprehensive assessment of the inter-relationship of CNAs and translocations in the Myeloma XI trial led to characterisation of genetic sub-groups with putative therapeutic relevance. We found that half of Myeloma XI tumors carried a deletion of NFκB-pathway genes, and 10% of tumors had two co-occuring deletions.^23, 24, 25^ Intriguingly, our data suggests NFκB-inducing kinase (NIK)-specific addiction of the t(4;14) group: *BIRC2/BIRC3* deletions, including homozygous deletions, were enriched in t(4;14) tumours. The t(4;14) MM without *BIRC2/BIRC3* deletions were frequently *TRAF3* deleted. BIRC2, BIRC3 and TRAF3 proteins all interact directly with NIK, suppressing NFκB-pathway activity.(25) MM cell lines with deletions of *BIRC2/BIRC3* or *TRAF3*, predominantly t(4;14), have high NIK levels and activated NFκB-pathway signalling, as demonstrated by Keats *et al.* Recently, specific NIK inhibitors have been developed which might be used to target high-risk t(4;14) MM.^26, 27, 28^ Virtually all *BIRC2/3* deletions were found in *FGFR3*-positive tumors. They were mutually exclusive of *TRAF3* deletions, which were present in *FGFR3*-negative tumors, a pattern which may indicate convergent evolution. Deletions of *FGFR3*, which often occur as loss of der14 that includes *TRAF3*, may constitute ‘collateral damage’ of NIK addiction in t(4;14).^15, 24, 25, 29, 30^

Although t(4;14) and del(17p) were not correlated with each other, both groups were strongly associated with hypodiploidy-associated lesions del(12p), del(13q) and del(22q).^16, 17, 31, 32^ This suggests the consequences of t(4;14) and del(17p) may share molecular mechanisms. Gain of chromosome 1q21 was strongly associated with t(4;14), but not with del(17p). Gain(1q21) was confirmed as a high-risk lesion that is independent of del(1p32).^33, 34, 35^

HRD MM constitutes the largest genetic sub-group of patients, with substantial heterogeneity.^17^ We describe two sub-groups of HRD with either gain(11q25) and *CCND1* biology or gain(1q21) and *CCND2* overexpression. These groups are similar to the D1 and D2 sub-groups of the TC classification, which pioneered biologic classification of HRD MM. Application of the TC classification in routine diagnostics has unfortunately been restricted due to access limitations to array-based gene expression profiling.^17^ Pragmatic classification of HRD based on gain(11q25) and gain(1q) may facilitate sub-grouping in clinical practice and open opportunities for improving therapy for these patients. Recently, activity of bcl-2 inhibitors has been reported in CCND1-driven t(11;14) MM, and CCND1-driven gain(11q25)-HRD may constitute another target group.^36^ We also found a high frequency of del(13q) in gain(1q)-HRD, in contrast to gain(11q25)-HRD. Interestingly, del(13q) and gain(1q) also frequently co-occur in t(4;14), suggesting similarities in the genetic sequelae of these pathogenetic groups.^37^ An inter-relationship between del(13q) and gain(1q)-HRD was suggested based on GEP in the TC classification by Bergsagel *et al.*, but has been demonstrated here for the first time on a DNA level.^17, 38^ Moreover, HRD MM without any of risk lesions gain(1q)-HRD, del(17p) and del(1p32) had longer remissions and survival than any other sub-group and may be sufficiently treated with single-novel agent/immunomodulatory drug-based approaches, potentially reducing additional side effects and costs of novel agent combinations.^39, 40, 41, 42^

In summary, we demonstrate the utility of profiling multiple molecular genetic lesions to identify patients most likely to benefit from molecularly targeted therapies. The molecular tools used for profiling Myeloma XI are readily applicable within diagnostic settings and should therefore help implementing stratified treatment approaches as part of routine patient care.

## Figures and Tables

**Table 1:** Clinical characteristics and frequency of genetic aberrations in myeloma IX and myeloma XI trial patients

	*Myeloma IX trial*		*Myeloma XI trial*		*P-value*
	*(Total* n=*869)*	*Missing information*	*(Total* n=*1036)*	*Missing information*	
*Clinical characteristics*					
Female	339 (39.0%)		398 (38.4%)		0.81
Male	530 (61.0%)		638 (61.6%)		0.81
Intensive treatment pathway	511 (58.8%)		598 (57.5%)		0.64
Non-intensive treatment pathway	358 (41.2%)		438 (42.3%)		0.64
ISS I	130 (20.7%)	240	225 (23.1%)	61	0.27
ISS II	253 (40.2%)	240	429 (44.0%)	61	0.15
ISS III	246 (39.1%)	240	321 (32.9%)	61	0.01
Median age (years)	65 (range 34–89)		67 (range 34–88)		1.0
					
*Primary lesions (translocations, HRD)*					
t(4;14)	104 (11.9%)		137 (13.2%)		0.45
t(4;14) FGFR3-negative	–		26 (2.5%)		
t(6;14)	8 (0.9%)	1	7 (0.7%)		0.61
t(11;14)	129 (14.8%)		175 (16.9%)		0.23
t(14;16)	27 (3.1%)		38 (3.7%)		0.53
t(14;20)	13 (1.5%)		13 (1.3%)		0.69
HRD	499 (58.9%)	22	488 (47.1%)		3 × 10^−7^
					
*Copy number abnormalities*					
Del(1p32)	87 (10.7%)	60	107 (10.3%)		0.82
Gain(1q) or Amp(1q)	340 (39.1%)		357 (34.5%)		0.04
Gain(1q)	–		277(26.7%)		
Amp(1q)	–		80 (7.7%)		
Gain(6p) or Amp(6p)	–		122 (12.1%)	29	
Gain(6q) or Amp(6q)	–		69 (6.9%)	29	
Del(6q)	–		157 (15.6%)	29	
Del(8p)	–		164 (16.3%)	29	
Gain(8q)	–		43 (4.3%)	29	
Gain(11q25)	–		418 (41.5%)	29	
Del(12p)	–		78 (7.5%)		
Del(13q)	389 (45.1%)	6	425 (41.0%)		0.07
Del(14q)	–		144 (13.9%)		
Del(16q)	153 (17.6%)	46	175 (16.9%)		0.36
Del(17p)	78 (8.9%)		96 (9.3%)		0.87
Del(22q)	100 (13.1%)	103	103 (10.2%)	29	0.04
					
*Focal copy number abnormalities/mutations*					
*CDKN2C* homozygous del	–		19 (1.8%)		
*BIRC2/BIRC3* homozygous del	–		22 (2.2%)	29	
*MYC* amplification	–		28 (2.8%)	29	
*CCND1* focal gain	–		46 (4.6%)	29	
*BRAF* V600E mutation	–		36 (3.6%)	29	

Abbreviations: HRD, hyperdiploid; ISS, International Staging System.

**Table 2:** Relationship between genetic abnormalities and patient survival

	*Myeloma IX* n=*869*		*Myeloma XI* n=*1036*		*Combined* n=*1905*		*Heterogeneity*
	*HR (95% CI)*	*P-value*	*HR (95% CI)*	*P-value*	*HR (95% CI)*	*P-value*	*P-value*
*(a) Progression-free survival*							
t(4;14)	1.88 (1.52–2.23)	5.31 × 10^−9^	1.51 (1.22–1.88)	0.0001	1.69 (1.45–1.96)	9.30 × 10^−12^	0.16
t(14;16)	1.50 (1.01–2.22)	0.0425	1.51 (1.05–2.17)	0.0256	1.50 (1.15–1.96)	0.0026	0.98
t(14;20)	1.13 (0.64–1.99)	0.6852	1.54 (0.80–2.97)	0.1987	1.29 (0.84–1.98)	0.2509	0.48
Adverse translocations	1.77 (1.47–2.13)	1.88 × 10^−9^	1.58 (1.31–1.91)	2.05 × 10^−6^	1.67 (1.46–1.91)	2.69 × 10^−14^	0.41
Del(17p)	1.54 (1.21–1.95)	0.0003	1.61 (1.26–2.06)	0.0002	1.57 (1.33–1.87)	2.07 × 10^−7^	0.79
Gain(1q)	1.53 (1.33–1.77)	6.70 × 10^−9^	1.53 (1.31–1.80)	1.34 × 10^−7^	1.53 (1.38–1.71)	4.61 × 10^−15^	1.00
Del(1p32)	0.99 (0.78–1.25)	0.9202	1.30 (1.02–1.66)	0.0331	1.13 (0.95–1.34)	0.1571	0.11
ISS II	1.40 (1.12–1.76)	0.0036	1.54 (1.23–1.92)	0.0002	1.47 (1.25–1.72)	2.50 × 10^−6^	0.58
ISS III	1.64 (1.30–2.06)	2.34 × 10^−5^	2.46 (1.96–3.09)	6.88 × 10^−16^	2.02 (1.71–2.37)	1.73 × 10^−17^	0.01
1 Adverse lesion	1.41 (1.21–1.65)	1.73 × 10^−5^	1.46 (1.23–1.74)	1.44 × 10^−5^	1.44 (1.28–1.61)	1.07 × 10^−9^	0.76
‘Double hit’ >1 adverse lesion	2.24 (1.83–2.76)	1.11 × 10^−14^	2.22 (1.78–2.77)	1.05 × 10^−12^	2.23 (1.92–2.59)	7.92 × 10^−26^	0.94
Intermediate risk-ISS	1.50 (1.25–1.79)	1.48 × 10^−5^	1.95 (1.63–2.33)	1.56 × 10^−13^	1.71 (1.51–1.95)	9.48 × 10^−17^	0.04
‘Double hit’-ISS	2.76 (2.13–3.57)	1.54 × 10^−14^	2.93 (2.29–3.09)	2 × 10^−16^	2.85 (2.38–3.40)	8.32 × 10^−31^	0.74

	*Myeloma IX* *n=869*		*Myeloma XI* *n=1036*		*Combined* *n=1905*		*Heterogeneity*
	*HR (95% CI)*	*P-value*	*HR (95% CI)*	*P-value*	*HR (95% CI)*	*P-value*	*P-value*
*(b) OS*							
t(4;14)	1.72 (1.36–2.17)	5.12 × 10^−6^	1.42 (1.06–1.91)	0.0188	1.60 (1.33–1.92)	4.77 × 10^−7^	0.33
t(14;16)	1.52 (0.98–2.35)	0.0607	2.00 (1.28–3.11)	0.0021	1.74 (1.27–2.37)	0.0005	0.39
t(14;20)	1.64 (0.88–3.07)	0.1213	2.35 (1.11–4.97)	0.0259	1.90 (1.17–3.07)	0.0089	0.47
Adverse translocations	1.74 (1.42–2.14)	1.15 × 10^−7^	1.71 (1.33–2.20)	3.23 × 10^−5^	1.73 (1.47–2.03)	1.63 × 10^−11^	0.90
Del(17p)	1.92 (1.49–2.48)	6.07 × 10^−7^	2.40 (1.77–3.24)	1.61 × 10^−8^	2.10 (1.73–2.56)	8.86 × 10^−14^	0.27
Gain(1q)	1.61 (1.37–1.91)	1.81 × 10^−8^	1.80 (1.44–2.24)	1.76 × 10^−7^	1.68 (1.47–1.92)	2.18 × 10^−14^	0.44
Del(1p32)	1.23 (0.94–1.61)	0.1170	1.83 (1.35–2.48)	0.0001	1.46 (1.20–1.78)	0.0002	0.06
ISS II	1.98 (1.47–2.68)	8.11 × 10^−6^	1.90 (1.30–2.77)	0.0009	1.95 (1.54–2.47)	2.76 × 10^−8^	0.86
ISS III	2.62 (1.94–3.53)	2.69 × 10^−10^	3.85 (2.66–5.56)	7.41 × 10^−14^	3.05 (2.42–3.85)	4.38 × 10^−21^	0.11
1 Adverse lesion	1.42 (1.18–1.71)	0.0002	1.81 (1.41–2.32)	3.57 × 10^−6^	1.55 (1.33–1.79)	9.97 × 10^−9^	0.13
‘Double hit’ >1 Adverse lesion	2.54 (2.02–3.18)	7.77 × 10^−16^	2.91 (2.17–3.89)	1.11 × 10^−12^	2.67 (2.23–3.19)	8.13 × 10^−27^	0.47
Intermediate risk-ISS	1.96 (1.57–2.45)	4.26 × 10^−9^	2.59 (1.96–3.41)	1.62 × 10^−11^	2.19 (1.84–2.61)	1.3 × 10^−18^	0.13
‘Double hit’-ISS	3.93 (2.93–5.27)	2 × 10^−16^	4.37 (3.13–6.12)	2 × 10^−16^	4.12 (3.30–5.14)	2.85 × 10^−36^	0.64

Abbreviations: CI, confidence interval; HR, hazard ratio; ISS, International Staging System; OS, overall survival.

**Table 3:** Relationship between genetic abnormalities and patient survival for intensively treated patients

	*Myeloma IX* *n=511*		*Myeloma XI* *n=598*		*Combined* *n=1109*		*Heterogeneity*
	*HR (95% CI)*	*P-value*	*HR (95% CI)*	*P-value*	*HR (95% CI)*	*P-value*	*P-value*
*(a) Progression-free survival*							
t(4;14)	1.96 (1.49–2.59)	1.80 × 10^−6^	2.03 (1.56–2.64)	2.18 × 10^−7^	2.00 (1.65–2.42)	1.85 × 10^−12^	0.88
t(14;16)	1.60 (0.96–2.69)	0.0729	2.03 (1.19–3.47)	0.0099	1.80 (1.23–2.60)	0.0021	0.54
t(14;20)	0.96 (0.46–2.03)	0.9192	0.64 (0.09–4.54)	0.6524	0.91 (0.45–1.84)	0.7987	0.70
Adverse translocations	1.81 (1.42–2.31)	1.79 × 10^−6^	2.09 (1.62–2.68)	8.88 × 10^−9^	1.94 (1.63–2.31)	1.07 × 10^−13^	0.42
Del(17p)	1.81 (1.30–2.51)	0.0004	1.81 (1.29–2.52)	0.0005	1.81 (1.43–2.28)	7.25 × 10^−7^	1.00
Gain(1q)	1.48 (1.22–1.80)	7.44 × 10^−5^	1.65 (1.31–2.07)	2.03 × 10^−5^	1.55 (1.34–1.80)	7.59 × 10^−9^	0.49
Del(1p32)	1.05 (0.76–1.47)	0.7556	1.48 (1.04–2.09)	0.0286	1.23 (0.97–1.57)	0.0833	0.17
ISS II	1.34 (1.01–1.77)	0.0409	1.48 (1.11–1.99)	0.0085	1.40 (1.15–1.72)	0.0009	0.61
ISS III	1.43 (1.07–1.91)	0.0168	2.20 (1.61–3.01)	7.88 × 10^−7^	1.74 (1.40–2.16)	3.11 × 10^−7^	0.04
1 Adverse lesion	1.50 (1.21–1.85)	0.0002	1.49 (1.15–1.93)	0.0024	1.50 (1.27–1.76)	1.36 × 10^−6^	0.99
‘Double hit’ >1 adverse lesion	2.31 (1.75–3.05)	3.67 × 10^−14^	3.00 (2.24–4.02)	2.17 × 10^−13^	2.61 (2.13–3.20)	1.07 × 10^−20^	0.21
Intermediate risk-ISS	1.47 (1.16–1.86)	0.0015	1.87 (1.45–2.41)	1.45 × 10^−6^	1.64 (1.38–1.95)	2.10 × 10^−8^	0.17
‘Double hit’-ISS	2.78 (1.96–3.95)	9.85 × 10^−9^	3.42 (2.47–4.75)	1.92 × 10^−13^	3.11 (2.45–3.95)	1.59 × 10^−20^	0.40

	*Myeloma IX* *n=511*		*Myeloma XI* *n=598*		*Combined* *n=1109*		*Heterogeneity*
	*HR (95% CI)*	*P-value*	*HR (95% CI)*	*P-value*	*HR (95% CI)*	*P-value*	*P-value*
*(b) OS*							
t(4;14)	1.74 (1.26–2.41)	0.0008	2.09 (1.41–3.07)	0.0002	1.87 (1.46–2.40)	7.62 × 10^−7^	0.49
t(14;16)	1.51 (0.79–2.84)	0.2059	2.31 (1.13–4.73)	0.0218	1.82 (1.13–2.92)	0.01359	0.38
t(14;20)	1.44 (0.59–3.49)	0.4181	1.91 (0.26–13.70)	0.5219	1.51 (0.67–3.39)	0.3169	0.80
Adverse translocations	1.74 (1.30–2.33)	0.0002	2.30 (1.60–3.31)	6.99 × 10^−6^	1.94 (1.55–2.43)	9.91 × 10^−9^	0.24
Del(17p)	2.31 (1.61–3.31)	5.87 × 10^−6^	3.19 (2.10–4.85)	5.74 × 10^−8^	2.65 (2.01–3.48)	3.04 × 10^−12^	0.25
Gain(1q)	1.79 (1.40–2.27)	2.33 × 10^−6^	1.72 (1.22–2.43)	0.0020	1.77 (1.45–2.15)	1.65 × 10^−8^	0.86
Del(1p32)	1.84 (1.28–2.64)	0.0010	1.99 (1.25–3.18)	0.0037	1.89 (1.42–2.52)	1.23 × 10^−5^	0.78
ISS II	1.96 (1.32–2.90)	0.0008	1.88 (1.14–3.11)	0.0140	1.92 (1.41–2.63)	3.27 × 10^−5^	0.90
ISS III	2.56 (1.72–3.81)	3.59 × 10^−6^	3.22 (1.94–5.35)	6.56 × 10^−6^	2.79 (2.04–3.81)	1.30 × 10^−10^	0.49
1 Adverse lesion	1.73 (1.32–2.26)	6.91 × 10^−5^	1.62 (1.08–2.44)	0.0207	1.69 (1.35–2.12)	4.30 × 10^−6^	0.79
‘Double hit’ >1 Adverse lesion	2.84 (2.05–3.94)	3.70 × 10^−10^	3.88 (2.55–5.92)	2.98 × 10^−10^	3.19 (2.47–4.14)	1.23 × 10^−18^	0.25
Intermediate risk-ISS	2.36 (1.71–3.25)	1.44 × 10^−7^	2.26 (1.49–3.43)	0.0001	2.32 (1.80–3.00)	7.15 × 10^−11^	0.87
‘Double hit’-ISS	4.51 (2.97–6.83)	1.26 × 10^−12^	5.18 (3.24–8.27)	5.78 × 10^−12^	4.79 (3.51–6.54)	5.10 × 10^−23^	0.66

Abbreviations: CI, confidence interval; HR, hazard ratio; ISS, International Staging System; OS, overall survival.
