# Supplementary material for: Prediction of outcome in newly diagnosed myeloma: a meta-analysis of the molecular profiles of 1905 trial patients
Source: Leukemia. 2017 Jun 30;32(1):102–10. doi: 10.1038/leu.2017.179 (PMC5590713; doi:10.1038/leu.2017.179)
Supplement: Supplementary Information [file leu2017179x1.docx]

**SUPPLEMENTARY MATERIAL AND METHODS AND TABLES**

THE MYELOMA XI TRIAL

Patients were randomised between May 2010 and February 2016 to either cyclophosphamide, dexamethasone with thalidomide (CTD) or revlimid (CRD) IMiD induction therapy. Patients not responding to IMiDs (progressive or stable disease) received velcade-based therapy (CVD) and patients with suboptimal response (partial response) were randomised to either CVD or no further treatment prior to consolidation/maintenance. Young and fit patients received consolidation with high-dose melphalan (HD-MEL) and autologous stem cell transplant (ASCT). Patients underwent a second randomisation to receive revlimid, revlimid and vorinostat or no maintenance therapy (Supplementary CONSORT diagram).

THE MYELOMA IX TRIAL

Briefly, the trial recruited newly diagnosed patients of all ages between May 2003 and May 2008 who were randomised to receive either alkylating therapy (CVAD or MP) or thalidomide-based induction therapy (CTD). Younger and fitter patients received HD-MEL+ASCT (‘intensive’ treatment arm). Patients were subsequently randomised to thalidomide maintenance or no maintenance.

MULTIPLEX LIGATION PROBE AMPLIFICATION (MLPA)

Interrogated chromosomal areas with frequent CNAs are shown in Supplementary Table 1. MLPA PCR products were analysed on an ABI 3730 DNA analyser (Life Technologies, Paisley, UK) and raw data was analysed with Coffalyser.net software (MRC Holland, Amsterdam, The Netherlands).

Multiplexed real-time quantitative RT-PCR was used to assess expression of *IGH* translocation partner genes *MMSET*, *FGFR3*, *CCND1*, *CCND3*, *MAF*, *MAFB* as well as downstream effectors *CCND2*, *CX3CR1*, *ITGB7* with *GAPDH* normalisation. A FISH-validated TC classification-based hierarchical algorithm was applied to determine *IGH* translocation status, as previously described.

Supplementary Table 1: MLPA probe mix used in the Myeloma XI trial

Supplementary Table 2

Supplementary Table 3.

Multivariate analysis of ‘double-hit’ genetic high risk and ISS in 981 cases with complete information in Myeloma XI.

1. PFS

| **Variable** | **HR** | **95% Cl** | ***P*** |
| --- | --- | --- | --- |
| 1 adverse lesion | 1.41 | 1.18-1.68 | 0.000143 |
| ‘double-hit’ >1 Adverse lesion | 2.05 | 1.63-2.56 | 4.56x10^-10^ |
| ISS 2 | 1.48 | 1.18-1.85 | 0.000719 |
| ISS 3 | 2.30 | 1.83-2.89 | 8.26x10^-13^ |

1. OS

| **Variable** | **HR** | **95% Cl** | ***P*** |
| --- | --- | --- | --- |
| 1 adverse lesion | 1.709 | 1.32-2.22 | 5.25x10^-5^ |
| ‘double-hit’ >1 Adverse lesion | 2.577 | 1.90-3.49 | 1.02x10^-9^ |
| ISS 2 | 1.763 | 1.21-2.58 | 0.00346 |
| ISS 3 | 3.425 | 2.37-4.96 | 7.24x10^-11^ |

Supplementary Table 4.

Multivariate analysis of ‘double-hit’ genetic high risk and ISS in 567 cases treated on the intensive therapy arm with complete information in Myeloma XI.

1. PFS

| **Variable** | **HR** | **95% Cl** | ***P*** |
| --- | --- | --- | --- |
| 1 adverse lesion | 1.460 | 1.123-1.899 | 0.00468 |
| ‘double-hit’ >1 Adverse lesion | 2.678 | 1.978-3.627 | 1.88x10^-10^ |
| ISS 2 | 1.414 | 1.054-1.897 | 0.02100 |
| ISS 3 | 1.978 | 1.443-2.712 | 2.21x10^-5^ |

1. OS

| **Variable** | **HR** | **95% Cl** | ***P*** |
| --- | --- | --- | --- |
| 1 adverse lesion | 1.514 | 1.000-2.292 | 0.0497 |
| ‘double-hit’ >1 Adverse lesion | 3.166 | 2.045-4.901 | 2.35x10^-7^ |
| ISS 2 | 1.759 | 1.062-2.912 | 0.0282 |
| ISS 3 | 2.783 | 1.667-4.647 | 9.13x10^-5^ |

Supplementary Table 5.

Inference C-values using tau=24 months for PFS and tau=36 months for OS in Myeloma IX and Myeloma XI.

**PFS**

|  | **Myeloma IX**  n=869 | | | | **Myeloma XI**  n=1,036 | | | |
| --- | --- | --- | --- | --- | --- | --- | --- | --- |
|  | Estimate | Standard Error | Lower 95% CI | Upper 95% CI | Estimate | Standard Error | Lower 95% CI | Upper 95% CI |
| Adverse Translocations | 0.5456 | 0.0080 | 0.5299 | 0.5613 | 0.5387 | 0.0093 | 0.5206 | 0.5568 |
| t(4;14) | 0.5379 | 0.0069 | 0.5245 | 0.5513 | 0.5300 | 0.0086 | 0.5131 | 0.5468 |
| t(14;16) | 0.5058 | 0.0043 | 0.4974 | 0.5142 | 0.5062 | 0.0047 | 0.4971 | 0.5154 |
| t(14;20) | 0.5019 | 0.0035 | 0.4950 | 0.5088 | 0.5025 | 0.0033 | 0.4961 | 0.5089 |
| Del(17p) | 0.5149 | 0.0063 | 0.5026 | 0.5272 | 0.5287 | 0.0076 | 0.5139 | 0.5436 |
| Gain(1q) | 0.5576 | 0.0108 | 0.5364 | 0.5788 | 0.5415 | 0.0111 | 0.5198 | 0.5633 |
| Del(1p) | 0.5000 | 0.0072 | 0.4859 | 0.5141 | 0.5170 | 0.0082 | 0.5010 | 0.5330 |
| ISS | 0.5719 | 0.0140 | 0.5445 | 0.5992 | 0.6036 | 0.0125 | 0.5791 | 0.6282 |
| ‘double-hit’ | 0.5746 | 0.0118 | 0.5515 | 0.5978 | 0.5734 | 0.0117 | 0.5504 | 0.5963 |
| ‘double-hit’ - ISS | 0.5988 | 0.0131 | 0.5730 | 0.6245 | 0.6127 | 0.0123 | 0.5886 | 0.6369 |

**OS**

|  | **Myeloma IX**  n=869 | | | | **Myeloma XI**  n=1,036 | | | |
| --- | --- | --- | --- | --- | --- | --- | --- | --- |
|  | Estimate | Standard Error | Lower 95% CI | Upper 95% CI | Estimate | Standard Error | Lower 95% CI | Upper 95% CI |
| Adverse Translocations | 0.5501 | 0.0110 | 0.5285 | 0.5716 | 0.5418 | 0.0120 | 0.5184 | 0.5653 |
| t(4;14) | 0.5343 | 0.0094 | 0.5158 | 0.5528 | 0.5194 | 0.0116 | 0.4968 | 0.5421 |
| t(14;16) | 0.5096 | 0.0061 | 0.4977 | 0.5215 | 0.5163 | 0.0068 | 0.5029 | 0.5297 |
| t(14;20) | 0.5062 | 0.0046 | 0.4972 | 0.5152 | 0.5061 | 0.0038 | 0.4986 | 0.5136 |
| Del(17p) | 0.5289 | 0.0084 | 0.5125 | 0.5453 | 0.5462 | 0.0104 | 0.5259 | 0.5665 |
| Gain(1q) | 0.5711 | 0.0131 | 0.5455 | 0.5967 | 0.5658 | 0.0147 | 0.5369 | 0.5946 |
| Del(1p) | 0.5124 | 0.0108 | 0.4913 | 0.5335 | 0.5324 | 0.0101 | 0.5127 | 0.5521 |
| ISS | 0.5938 | 0.0160 | 0.5625 | 0.6251 | 0.6373 | 0.0155 | 0.6069 | 0.6678 |
| ‘double-hit’ | 0.5942 | 0.0146 | 0.5655 | 0.6229 | 0.6024 | 0.0156 | 0.5718 | 0.6330 |
| ‘double-hit - ISS | 0.6326 | 0.0160 | 0.6013 | 0.6639 | 0.6463 | 0.0151 | 0.6167 | 0.6759 |

Supplementary Table 6.

Inference C-values for intensively treated patients using tau=24 months for PFS and tau=36 months for OS in Myeloma IX and Myeloma XI.

**PFS**

|  | **Myeloma IX**  n=511 | | | | **Myeloma XI**  n=598 | | | |
| --- | --- | --- | --- | --- | --- | --- | --- | --- |
|  | Estimate | Standard Error | Lower 95% CI | Upper 95% CI | Estimate | Standard Error | Lower 95% CI | Upper 95% CI |
| Adverse Translocations | 0.5540 | 0.0122 | 0.5301 | 0.5778 | 0.5742 | 0.0159 | 0.5431 | 0.6052 |
| t(4;14) | 0.5458 | 0.0106 | 0.5252 | 0.5665 | 0.5653 | 0.0146 | 0.5367 | 0.5938 |
| t(14;16) | 0.5085 | 0.0065 | 0.4957 | 0.5213 | 0.5101 | 0.0067 | 0.4969 | 0.5232 |
| t(14;20) | 0.5004 | 0.0037 | 0.4931 | 0.5077 | 0.5012 | 0.0031 | 0.4952 | 0.5072 |
| Del(17p) | 0.5190 | 0.0083 | 0.5028 | 0.5352 | 0.5389 | 0.0123 | 0.5149 | 0.5630 |
| Gain(1q) | 0.5567 | 0.0152 | 0.5269 | 0.5865 | 0.5472 | 0.0160 | 0.5158 | 0.5785 |
| Del(1p) | 0.5016 | 0.0101 | 0.4819 | 0.5213 | 0.5239 | 0.0137 | 0.4971 | 0.5508 |
| ISS | 0.5575 | 0.0229 | 0.5128 | 0.6023 | 0.5969 | 0.0170 | 0.5636 | 0.6302 |
| ‘double-hit’ | 0.5873 | 0.0166 | 0.5547 | 0.6199 | 0.6003 | 0.0182 | 0.5647 | 0.6359 |
| ‘double-hit’ - ISS | 0.6039 | 0.0180 | 0.5686 | 0.6392 | 0.6300 | 0.0176 | 0.5955 | 0.6644 |

**OS**

|  | **Myeloma IX**  n=511 | | | | **Myeloma XI**  n=598 | | | |
| --- | --- | --- | --- | --- | --- | --- | --- | --- |
|  | Estimate | Standard Error | Lower 95% CI | Upper 95% CI | Estimate | Standard Error | Lower 95% CI | Upper 95% CI |
| Adverse Translocations | 0.5546 | 0.0172 | 0.5208 | 0.5884 | 0.5855 | 0.0227 | 0.5410 | 0.6301 |
| t(4;14) | 0.5352 | 0.0152 | 0.5054 | 0.5649 | 0.5595 | 0.0203 | 0.5198 | 0.5993 |
| t(14;16) | 0.5139 | 0.0131 | 0.4883 | 0.5394 | 0.5242 | 0.0123 | 0.5001 | 0.5482 |
| t(14;20) | 0.5056 | 0.0085 | 0.4889 | 0.5223 | 0.5018 | 0.0047 | 0.4927 | 0.5109 |
| Del(17p) | 0.5303 | 0.0136 | 0.5037 | 0.5570 | 0.5743 | 0.0186 | 0.5378 | 0.6108 |
| Gain(1q) | 0.5814 | 0.0204 | 0.5416 | 0.6213 | 0.5636 | 0.0258 | 0.5131 | 0.6141 |
| Del(1p) | 0.5399 | 0.0154 | 0.5098 | 0.5700 | 0.5420 | 0.0176 | 0.5075 | 0.5766 |
| ISS | 0.6107 | 0.0248 | 0.5621 | 0.6592 | 0.6280 | 0.0245 | 0.5800 | 0.6759 |
| ‘double-hit’ | 0.6163 | 0.0223 | 0.5726 | 0.6601 | 0.6338 | 0.0264 | 0.5821 | 0.6854 |
| ‘double-hit’ - ISS | 0.6600 | 0.0230 | 0.6149 | 0.7051 | 0.6682 | 0.0248 | 0.6196 | 0.7168 |

Supplementary Table 7

Relationship between copy number status of chromosome 1q and patient survival in Myeloma XI.

|  | **PFS**  **n=1036** | | | | **OS**  **n=1036** | | | |
| --- | --- | --- | --- | --- | --- | --- | --- | --- |
|  | **HR** | **p-value** | **Lower 95% CI** | **Upper 95% CI** | **HR** | **p-value** | **Lower 95% CI** | **Upper 95% CI** |
| **Gain(1q)** | 1.56 | 3.53x10^-7^ | 1.31 | 1.85 | 1.67 | 3.30x10^-5^ | 1.31 | 2.12 |
| **Amp(1q)** | 1.44 | 0.01 | 1.09 | 1.91 | 2.28 | 2.32x10^-6^ | 1.62 | 3.21 |
| **Gain(1q) vs Amp(1q)** | 0.91 | 0.54 | 0.68 | 1.23 | 1.36 | 0.09 | 0.95 | 1.95 |

Supplementary Table 8

Relationship between genetic abnormalities and patient survival for the hyperdiploid sub-group in Myeloma XI.

|  | **PFS**  **n=488** | | | | **OS**  **n=488** | | | |
| --- | --- | --- | --- | --- | --- | --- | --- | --- |
|  | **HR** | **p-value** | **Lower 95% CI** | **Upper 95% CI** | **HR** | **p-value** | **Lower 95% CI** | **Upper 95% CI** |
| **Gain(1q)** | 1.56 | 0.0003 | 1.22 | 1.99 | 1.81 | 0.001 | 1.27 | 2.59 |
| **Del(1p32)** | 1.66 | 0.006 | 1.16 | 2.37 | 2.33 | 0.0004 | 1.46 | 3.73 |
| **Del(17p)** | 1.30 | 0.23 | 0.85 | 1.99 | 1.89 | 0.02 | 1.08 | 3.30 |

**Supplementary Figure 1: Genetic risk markers and survival**

Kaplan-Meier curves and log-rank p-values for 1,036 NCRI Myeloma XI patients in the context of presence of absence of recurrent genetic aberrations.

1. Adverse Translocation
2. t(4;14)
3. t(14;16)
4. t(14;20)
5. del(17p)
6. gain(1q)
7. ‘double-hit’
8. ‘double-hit’-ISS

|  | PFS | OS |  | PFS | OS |
| --- | --- | --- | --- | --- | --- |
| a | 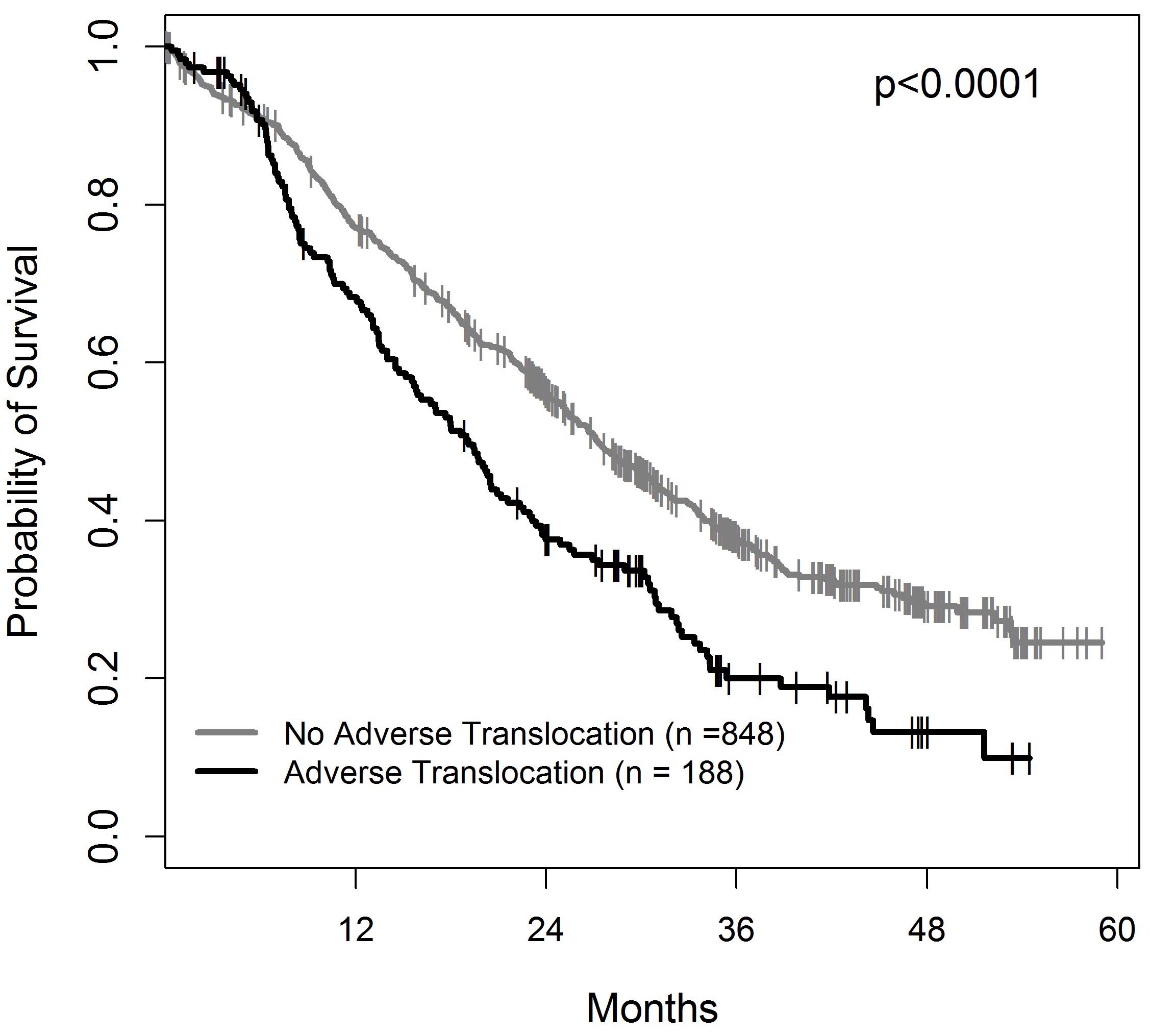 | 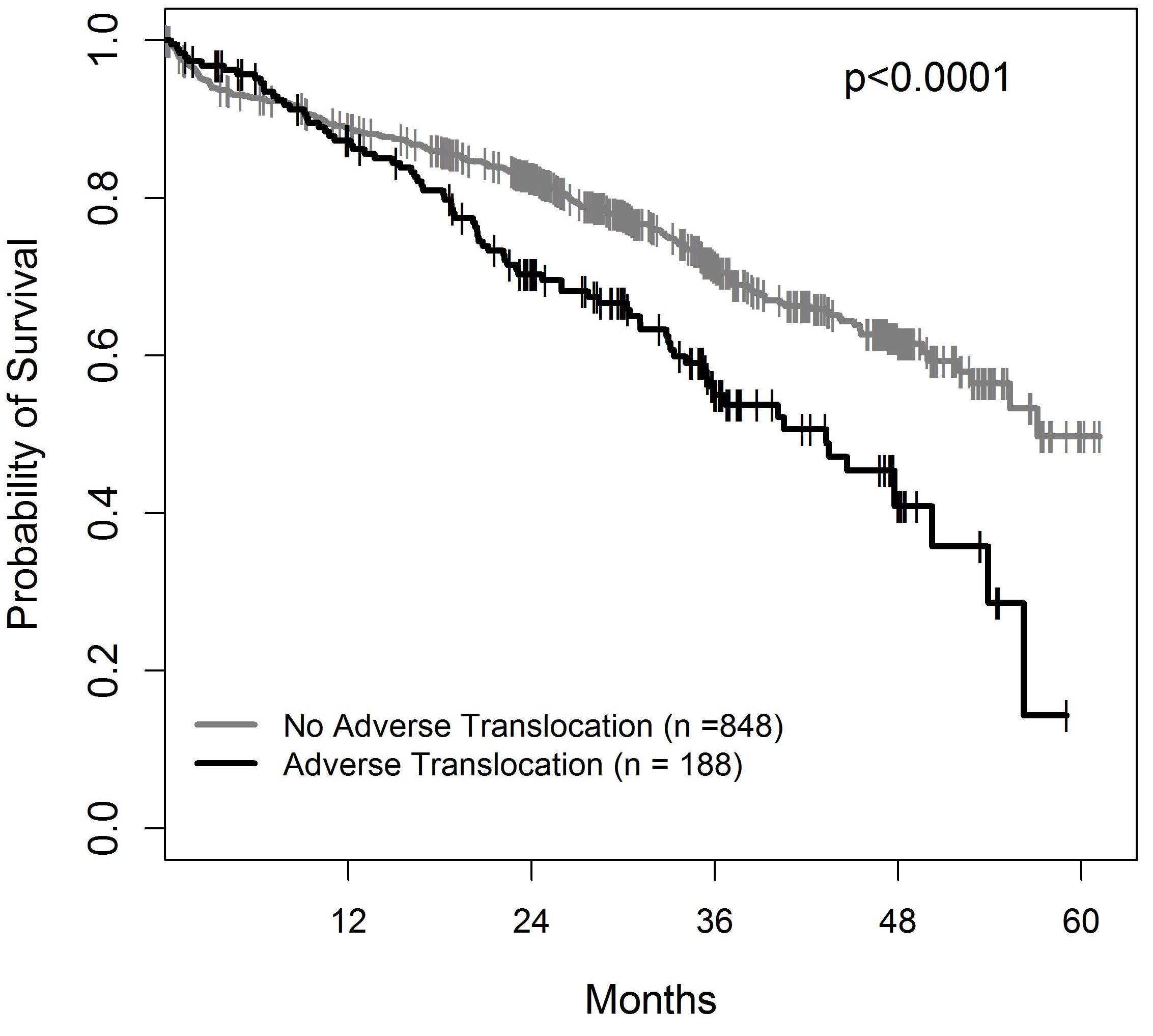 | e | 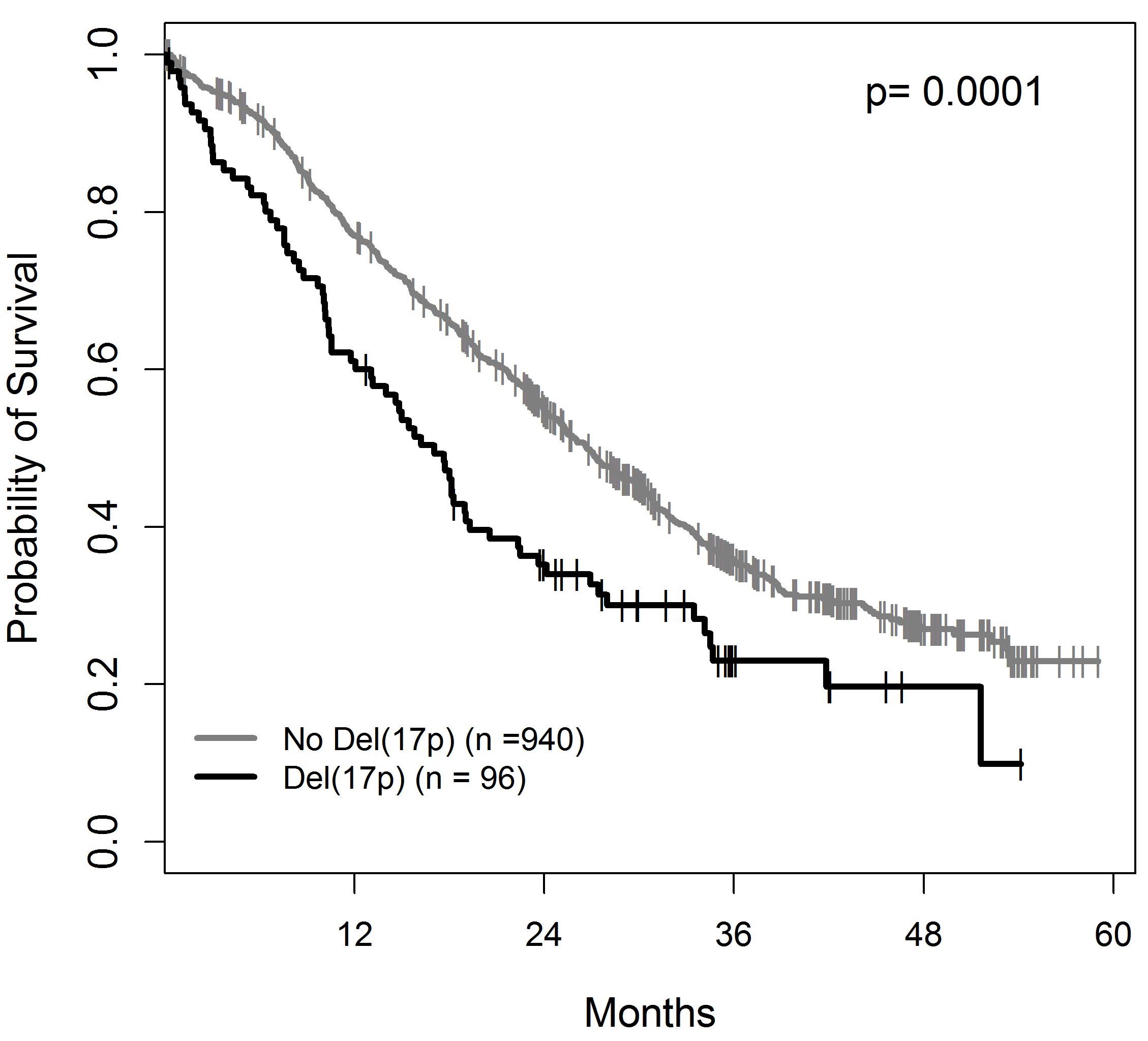 | 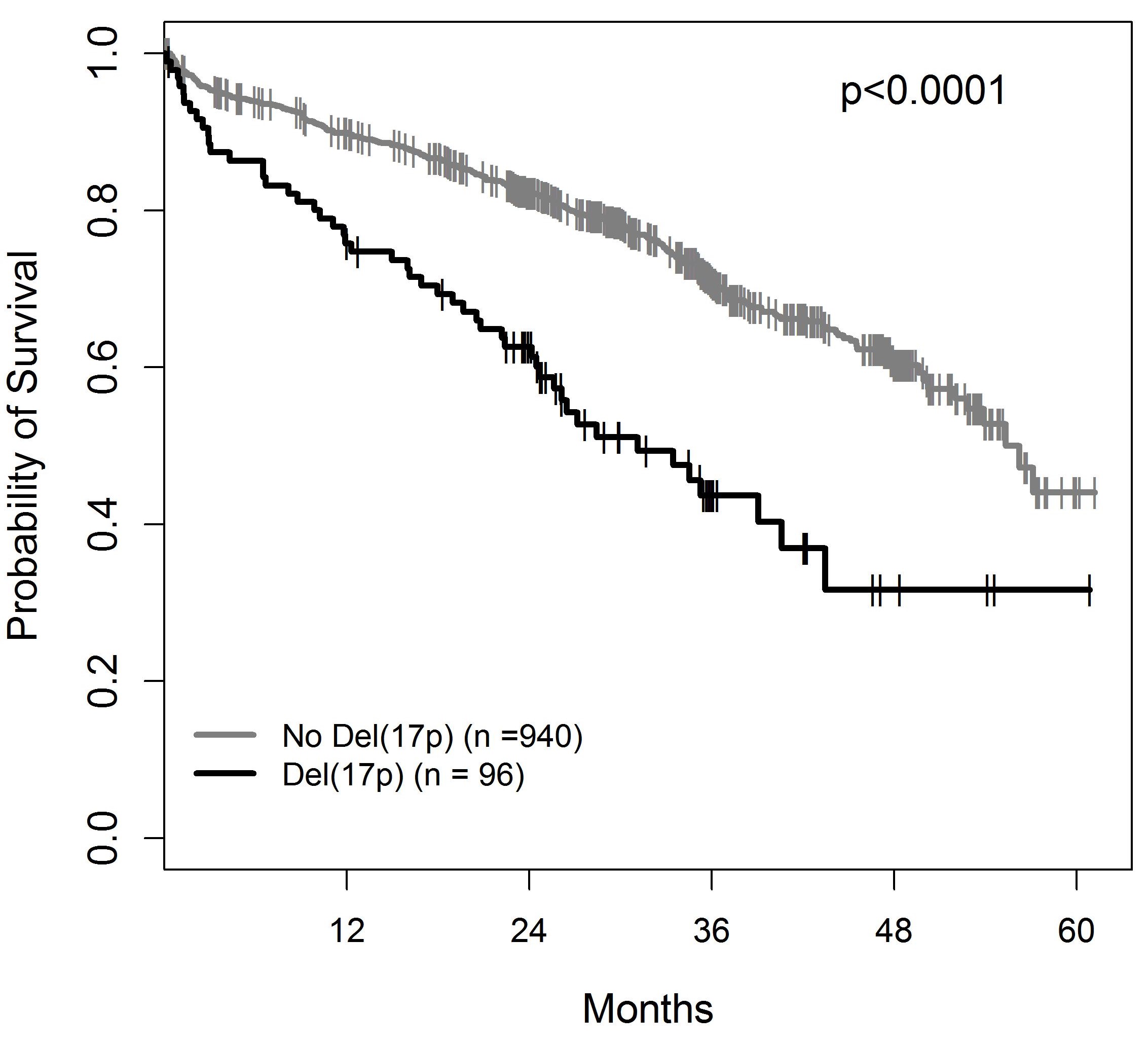 |
| b | 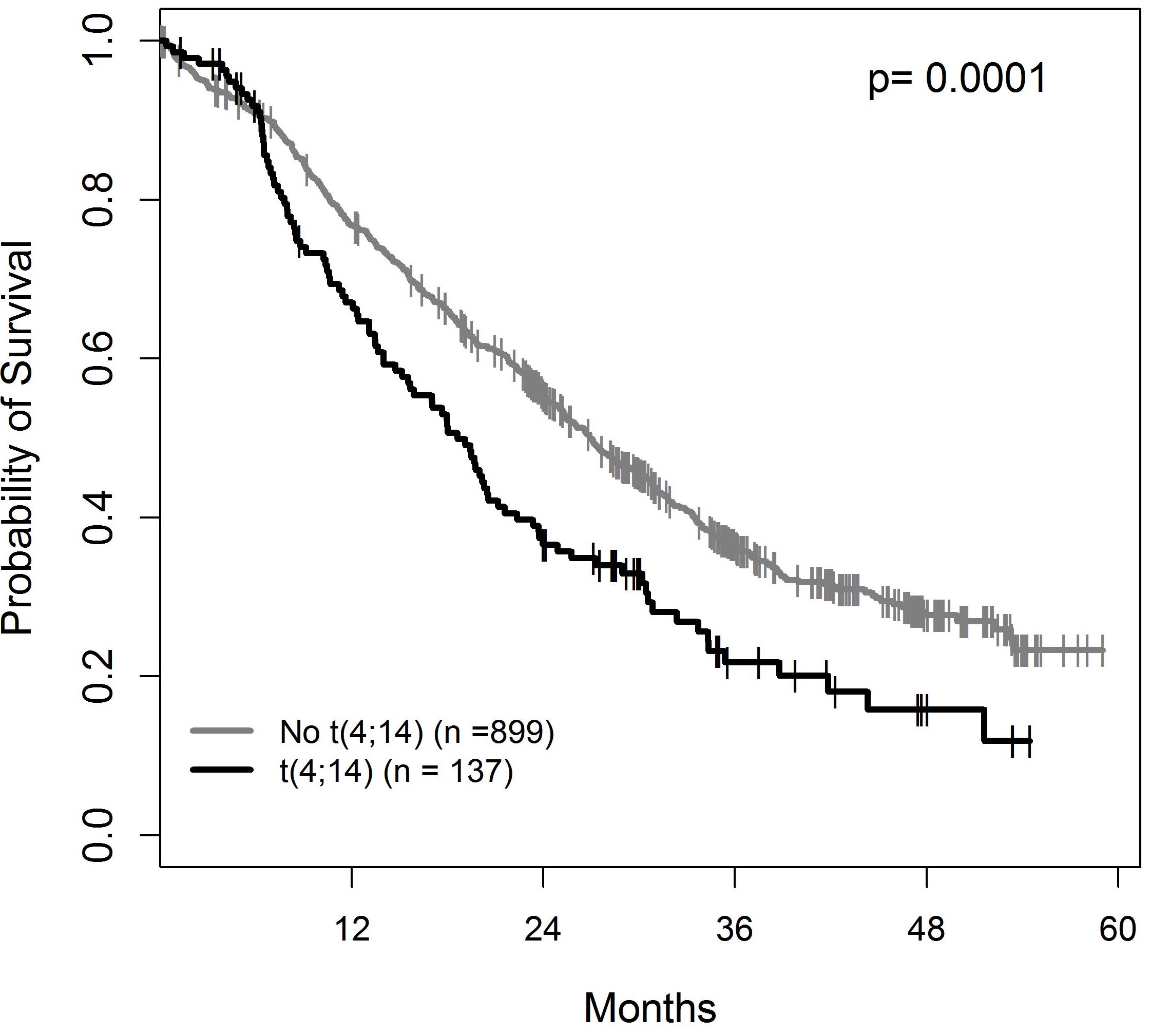 | 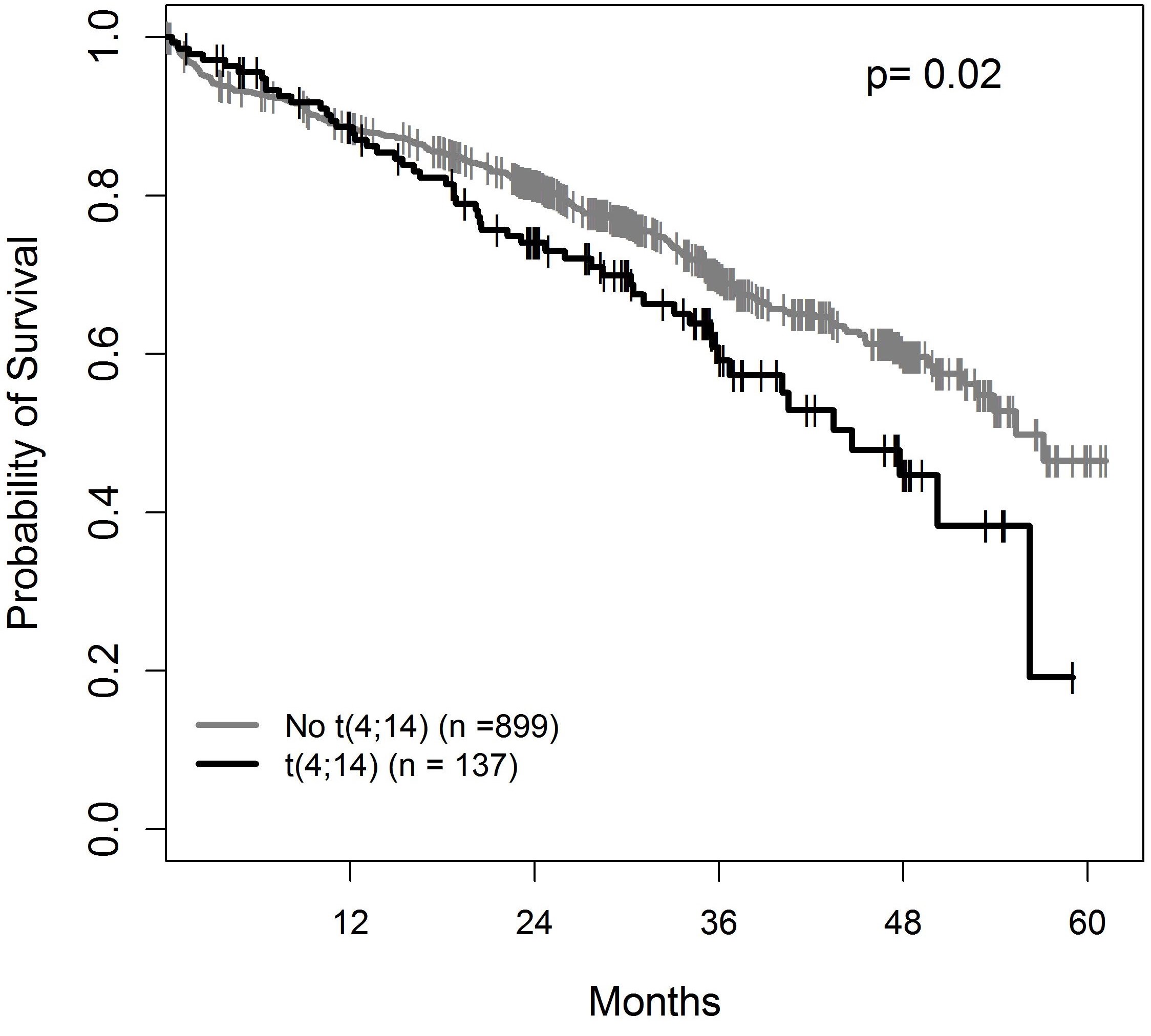 | f | 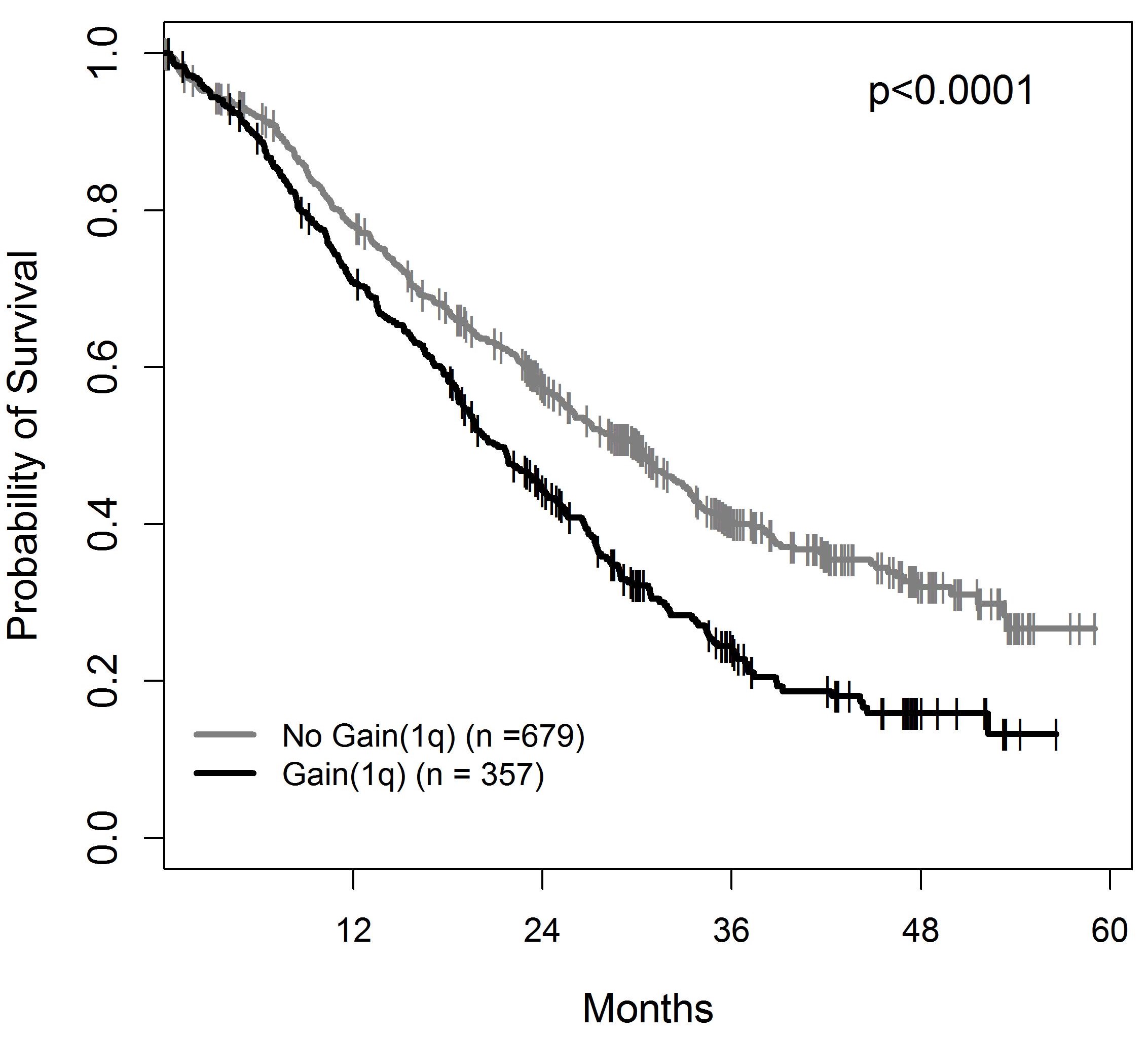 | 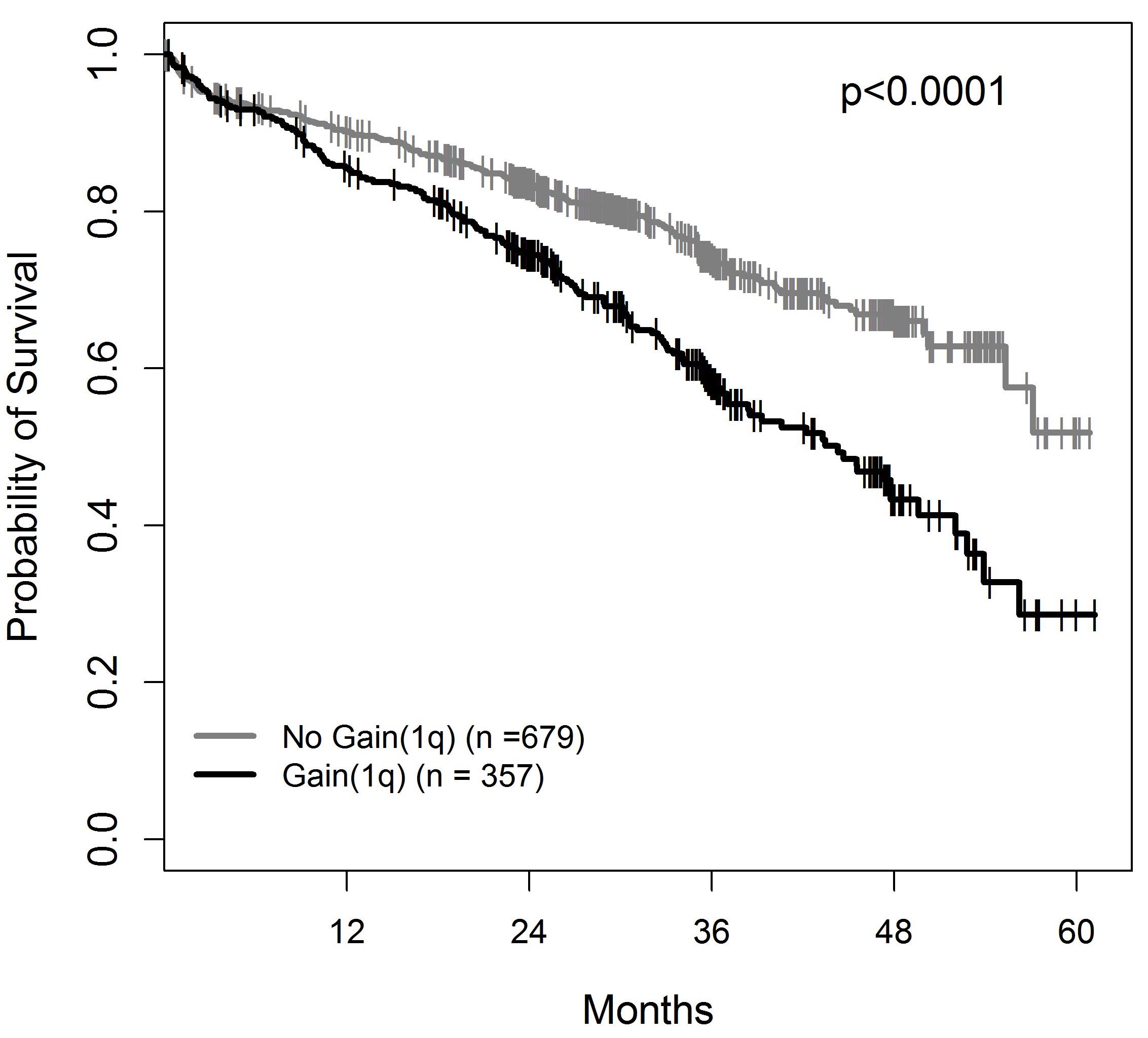 |
| c | 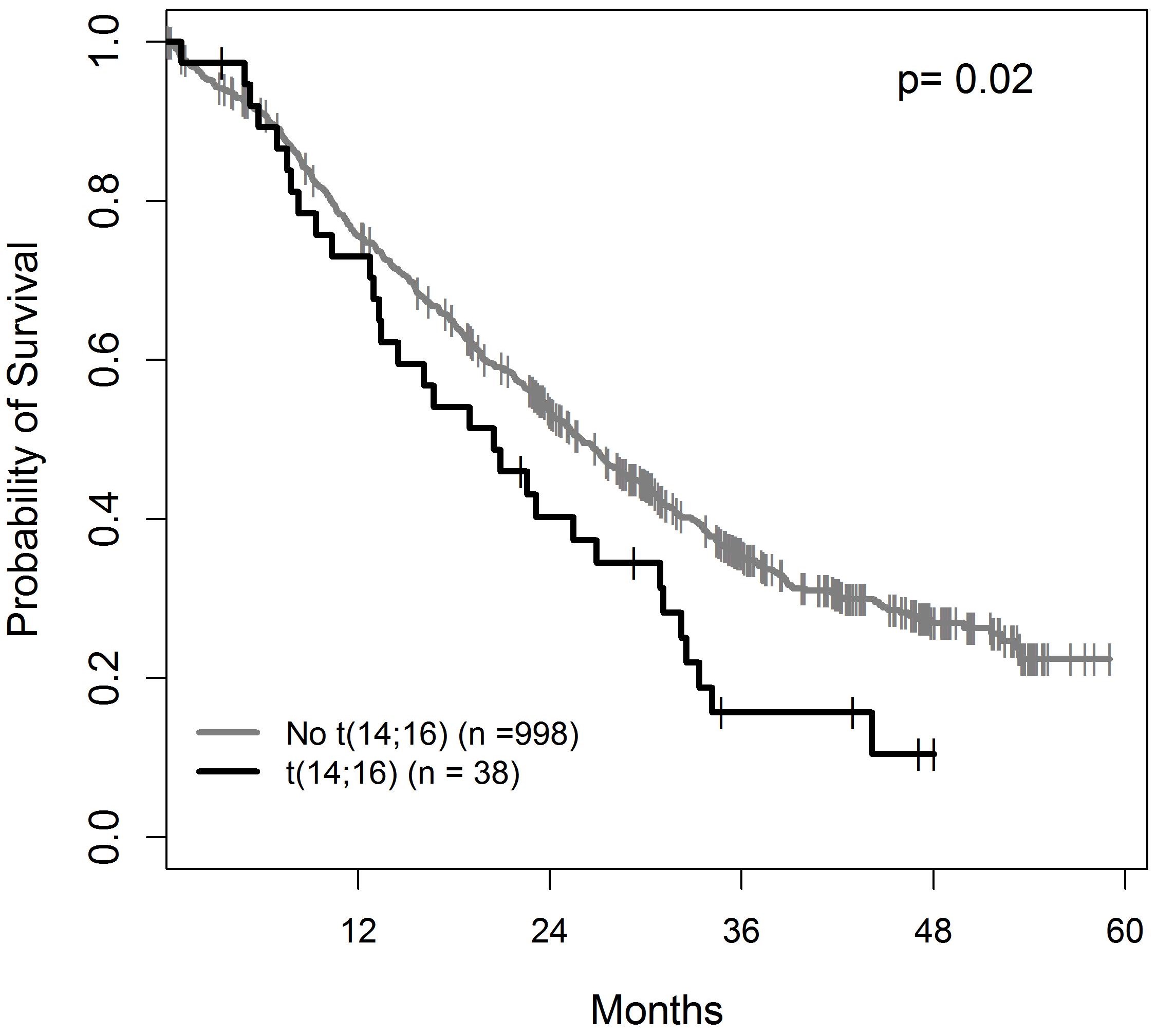 | 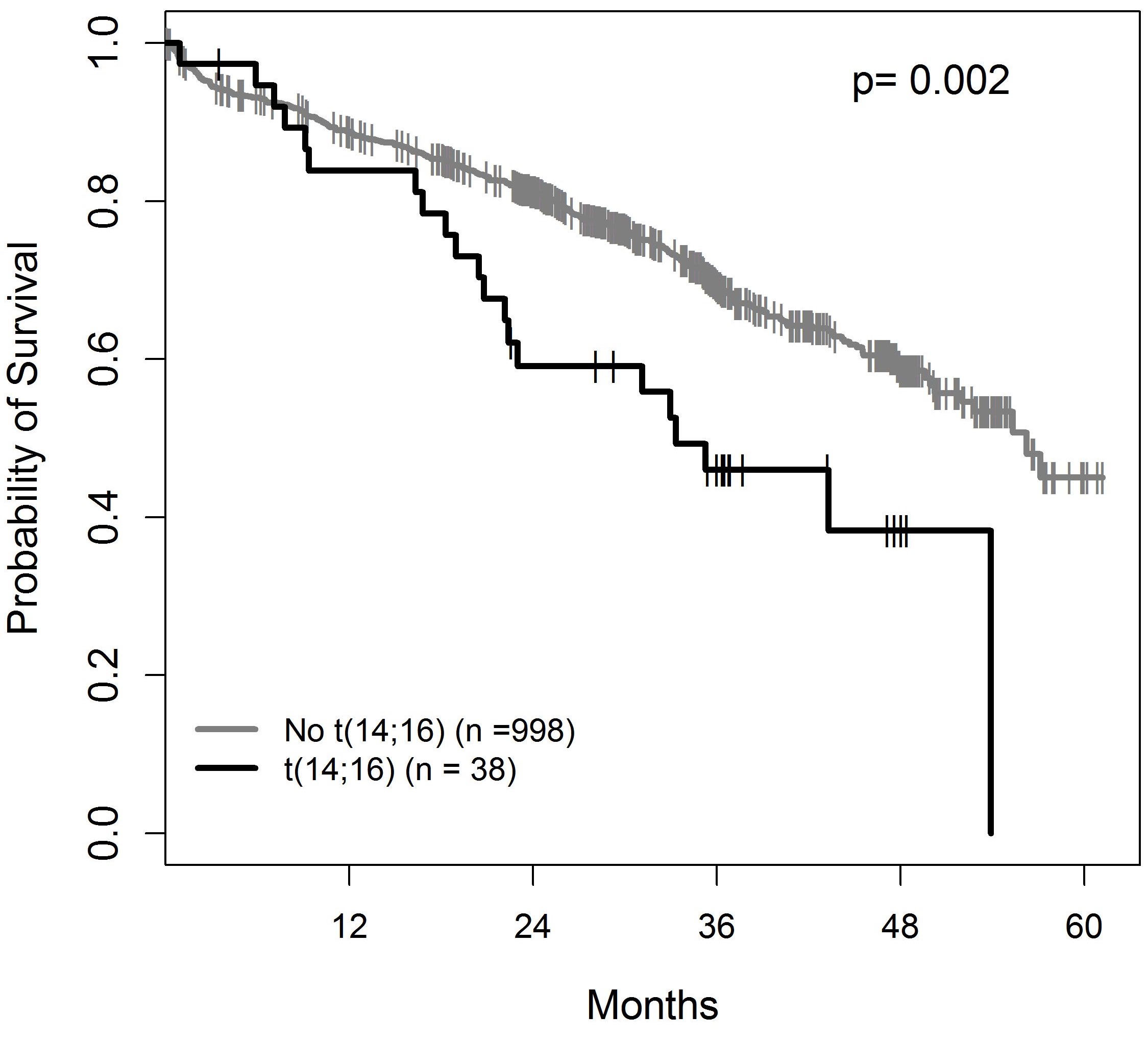 | g | 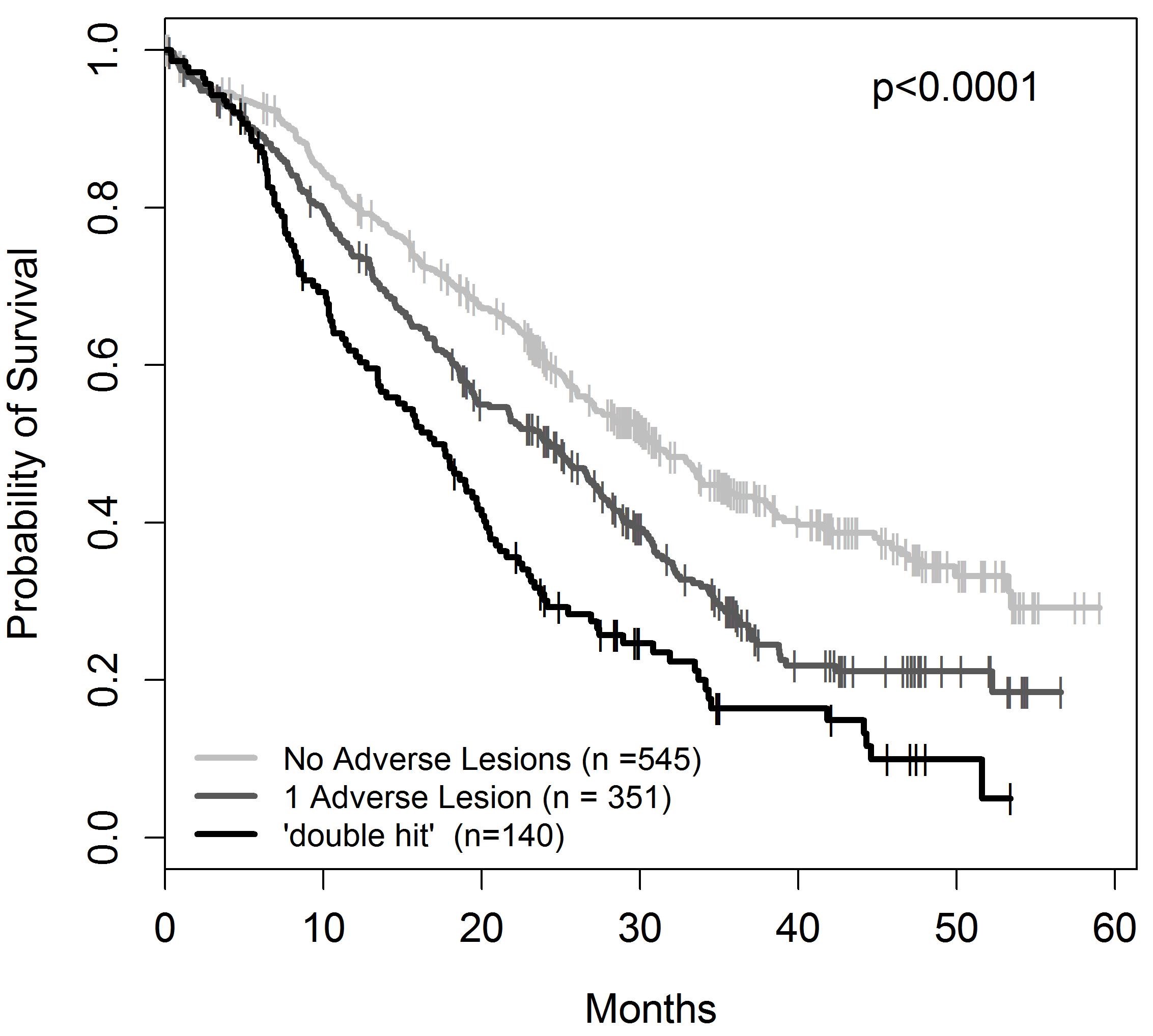 | 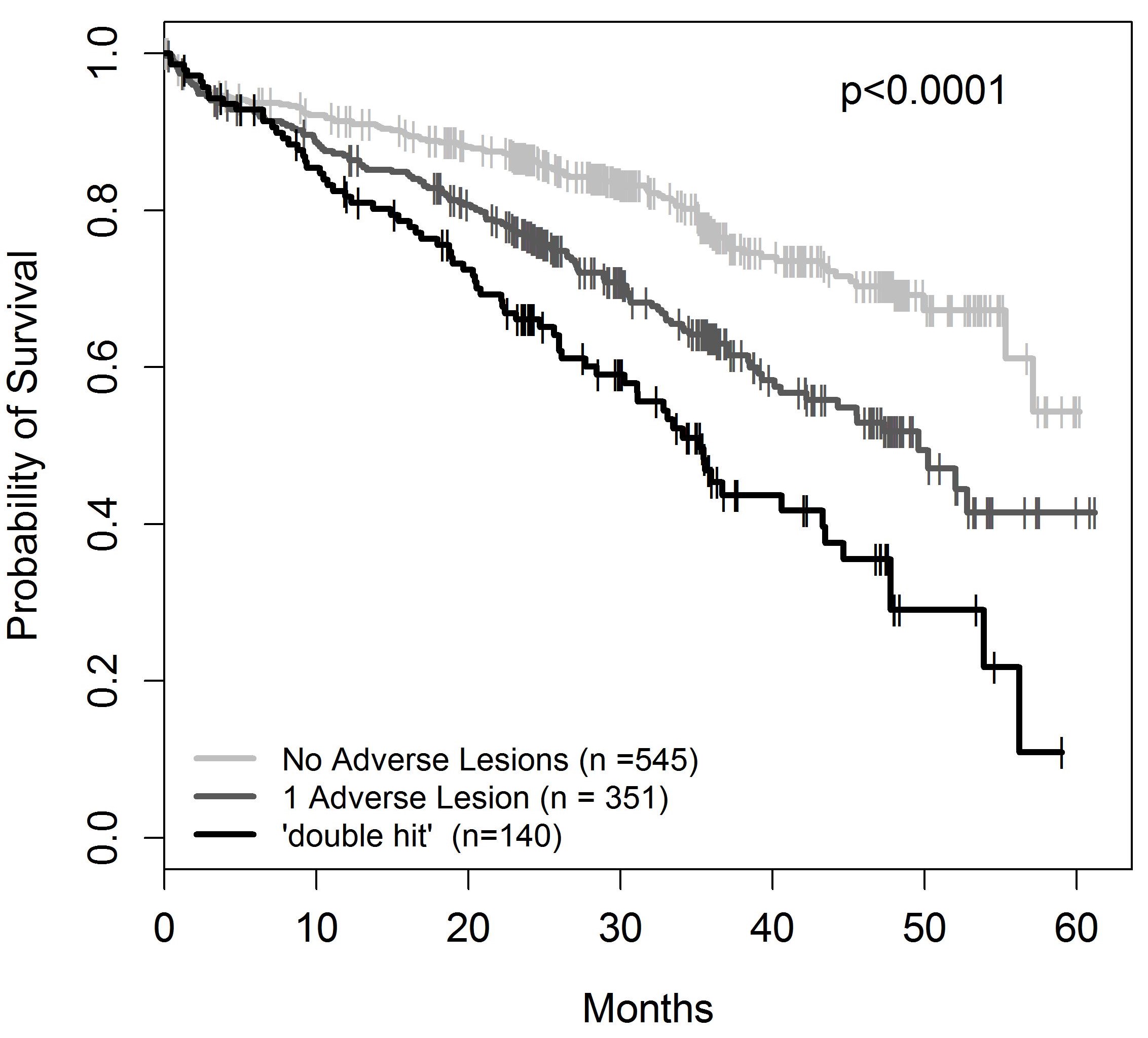 |
| d | 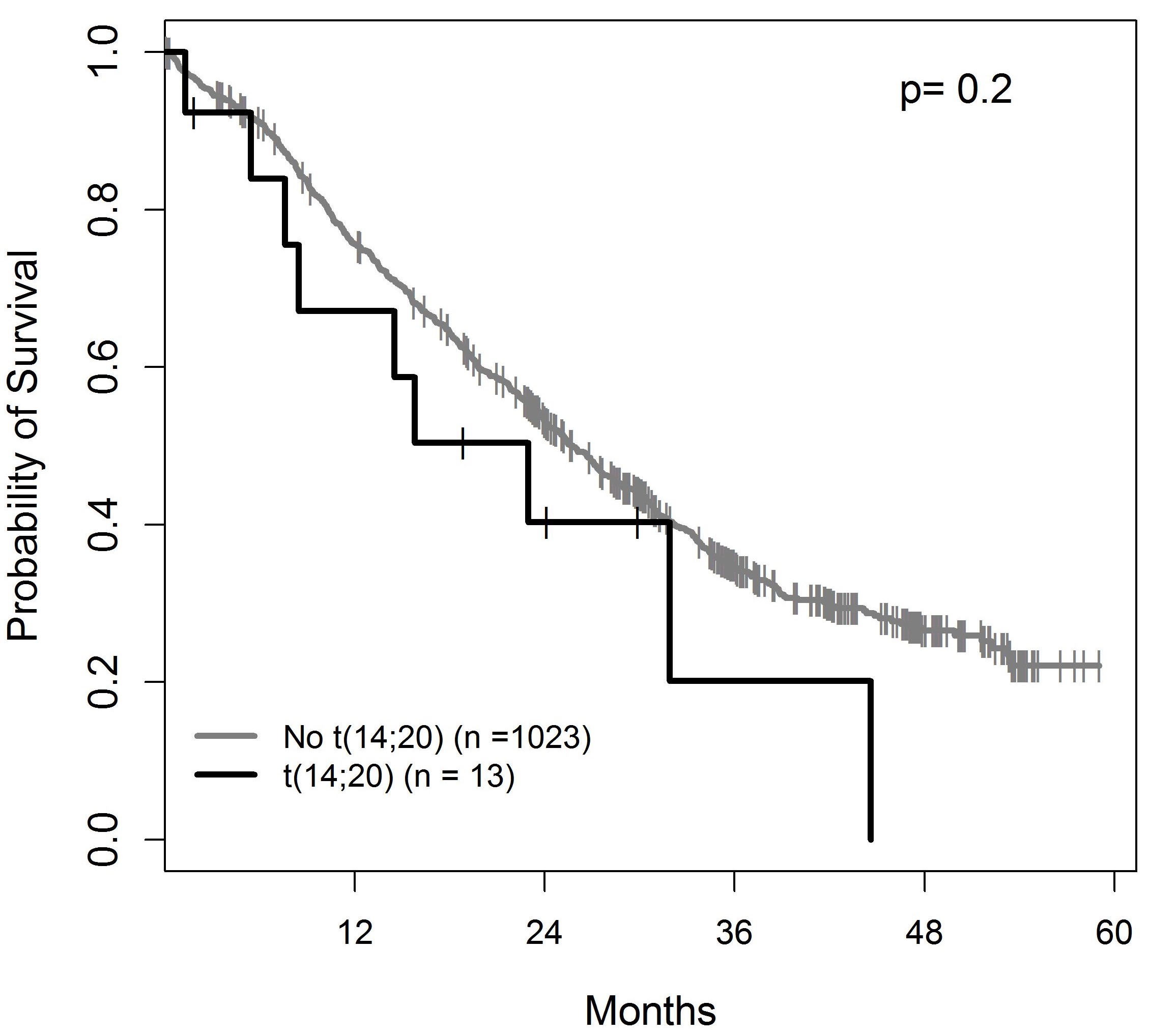 | 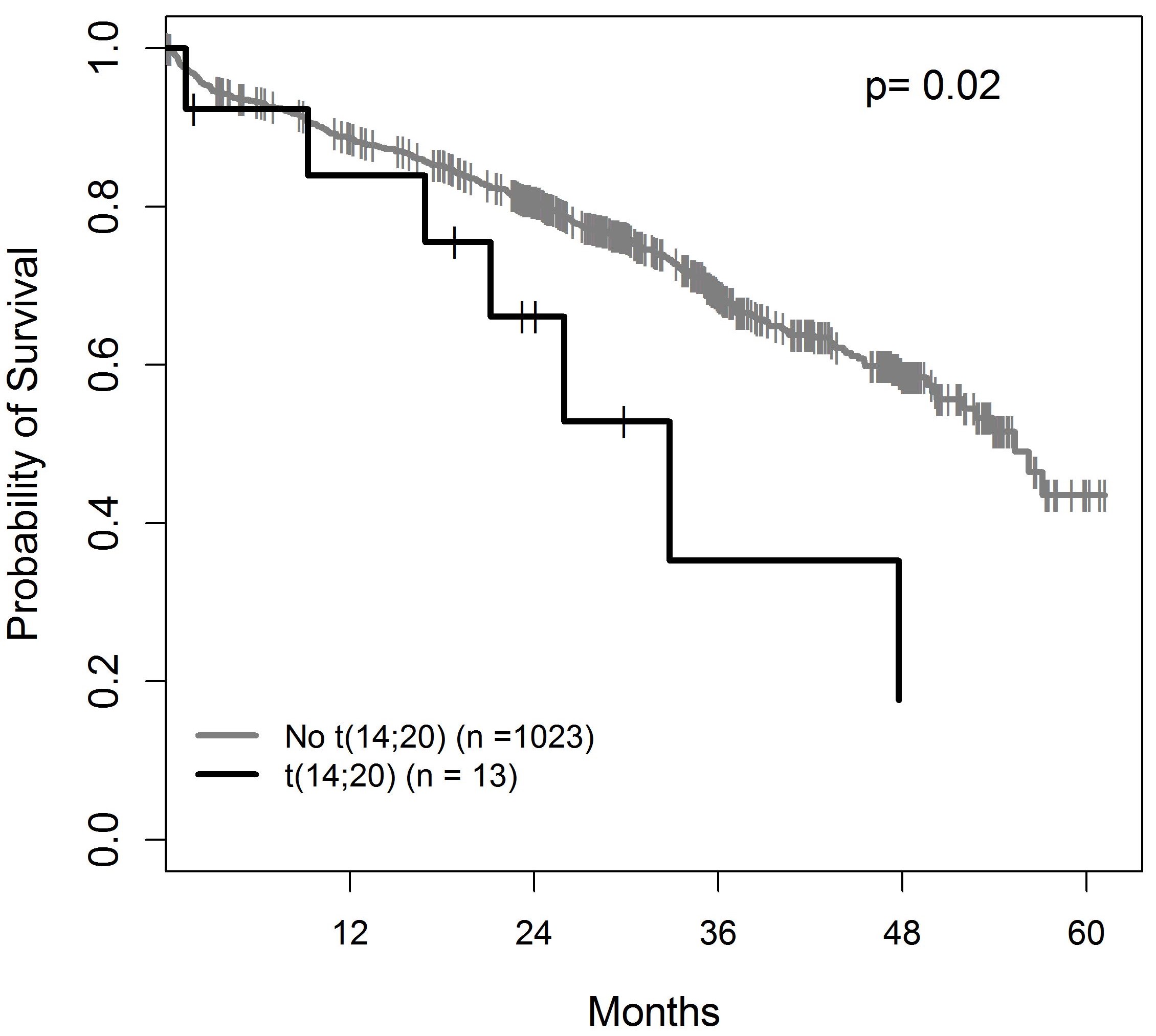 | h | 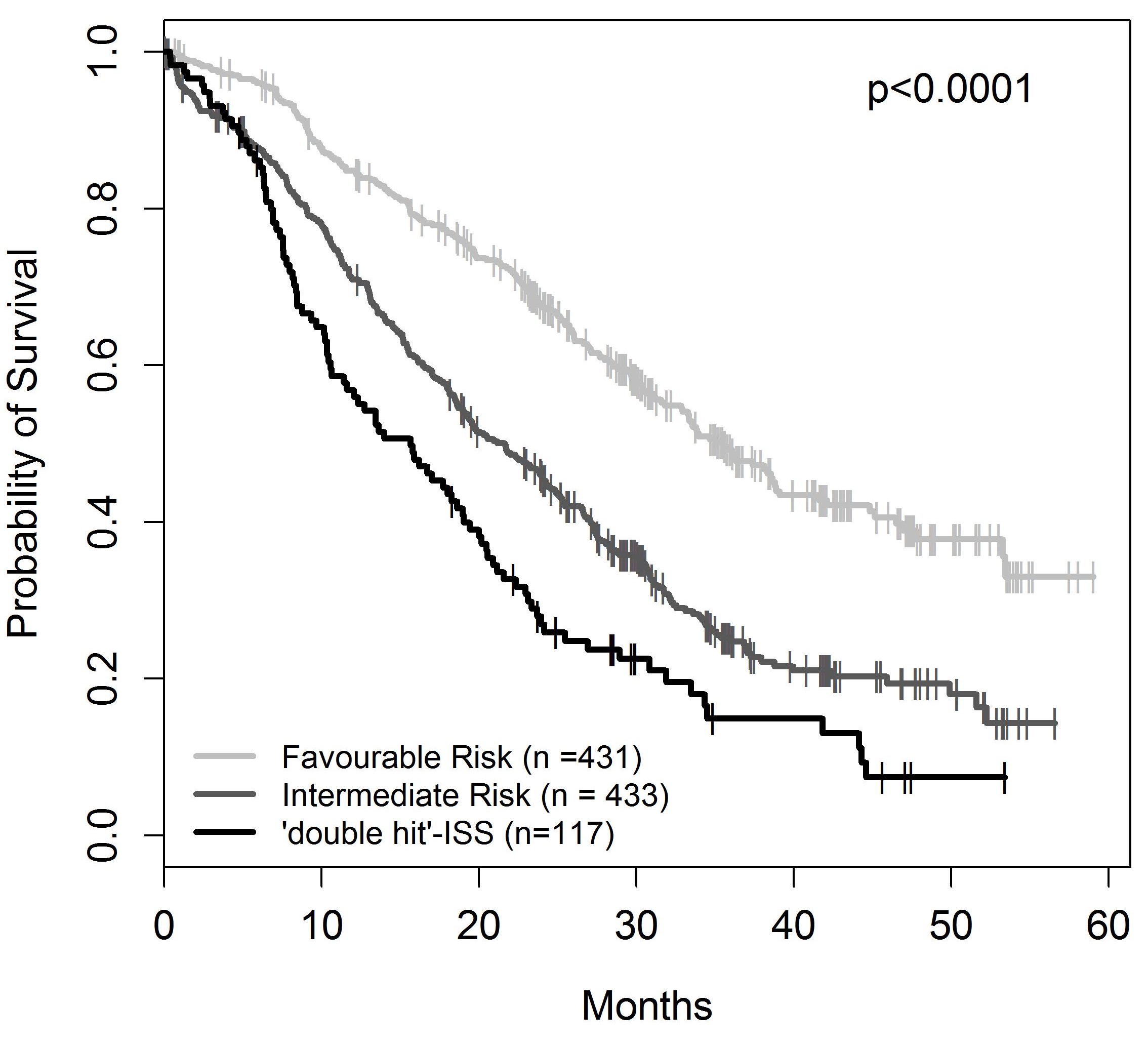 | 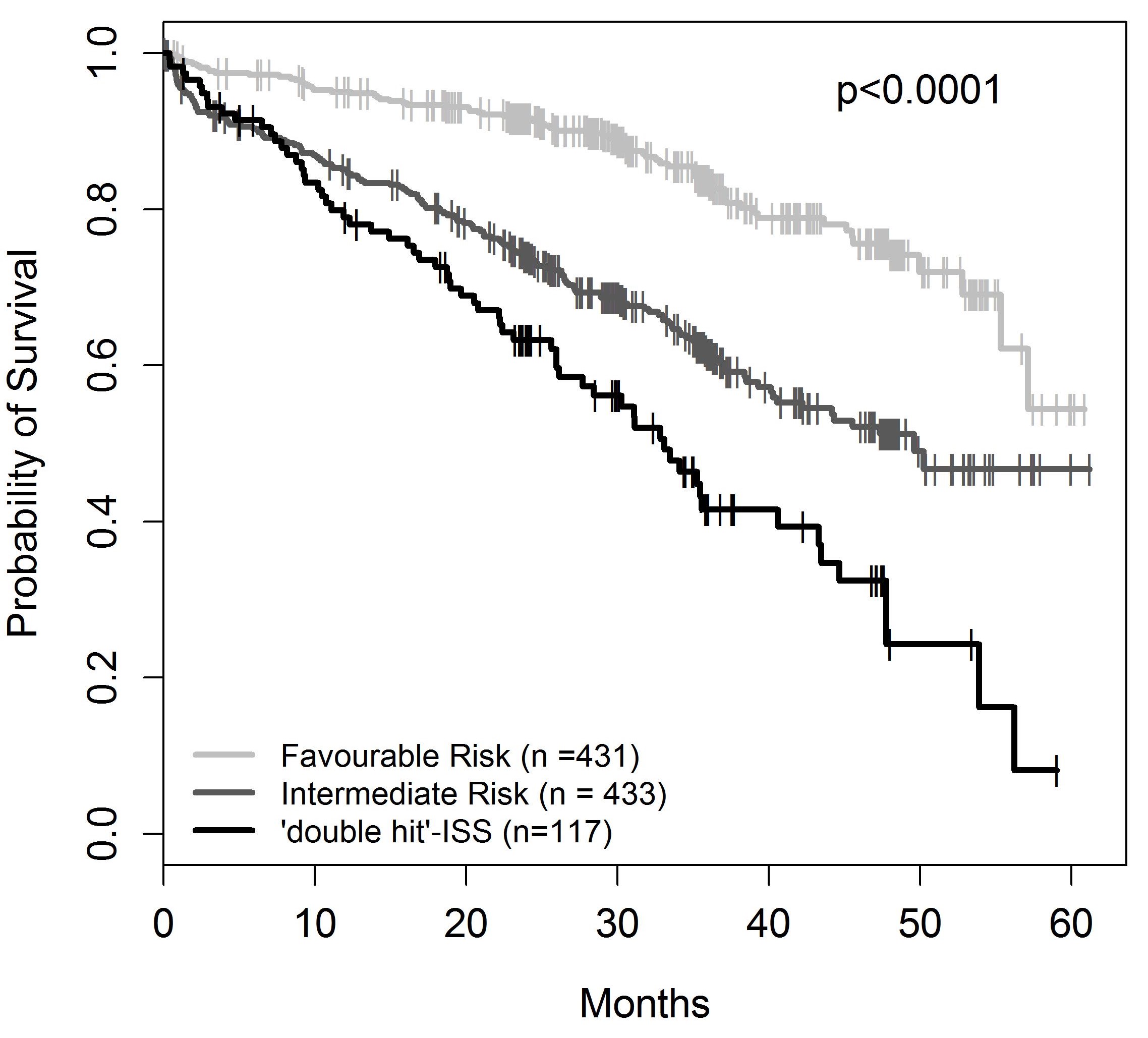 |

**Supplementary Figure 2: Genetic risk markers and survival**

Kaplan-Meier curves and log-rank p-values for 869 MRC Myeloma IX patients in the context of presence of absence of recurrent genetic aberrations.

1. Adverse Translocation
2. t(4;14)
3. t(14;16)
4. t(14;20)
5. del(17p)
6. gain(1q)
7. ‘double-hit’
8. ‘double-hit’-ISS

|  | PFS | OS |  | PFS | OS |
| --- | --- | --- | --- | --- | --- |
| a | 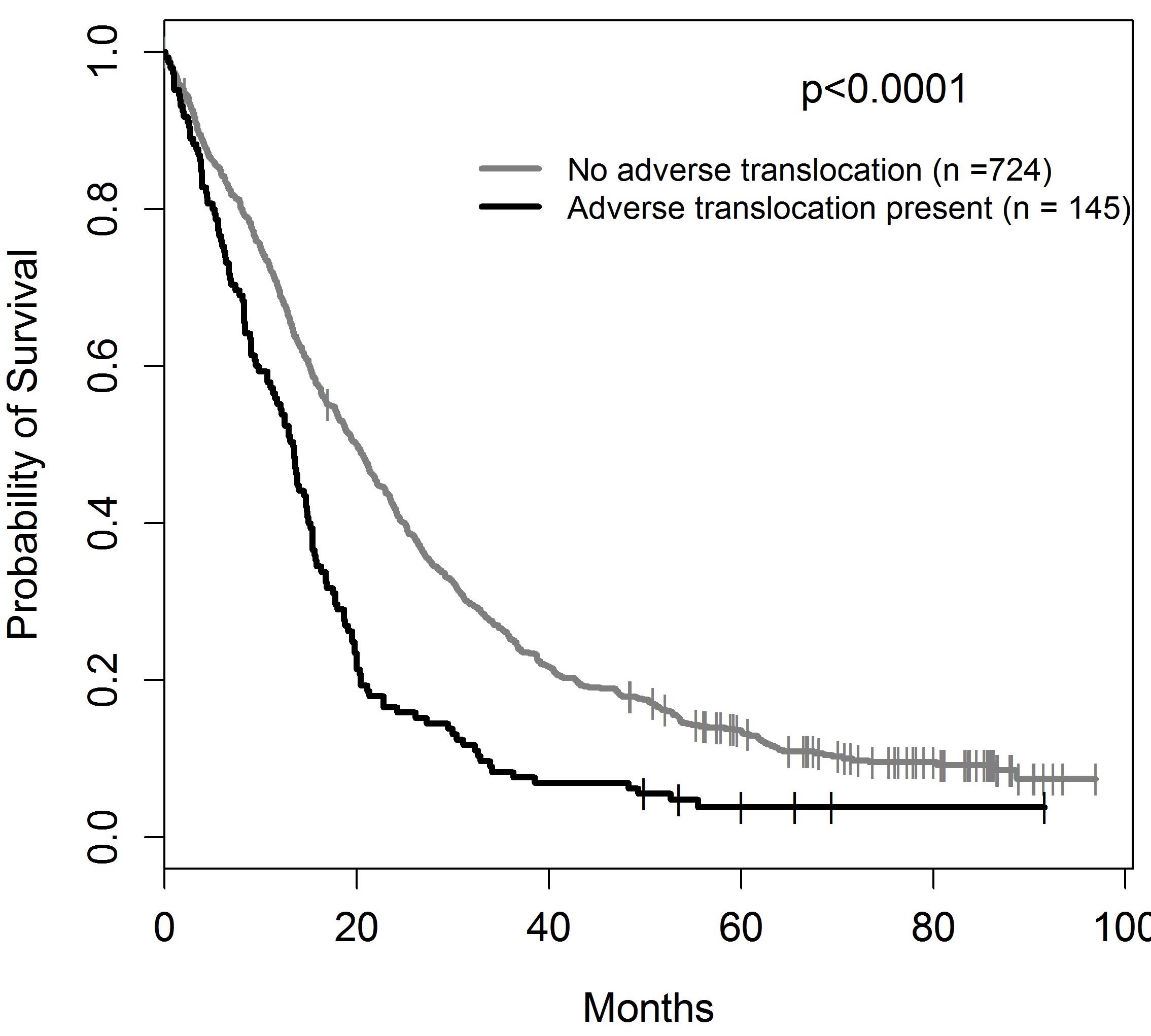 | 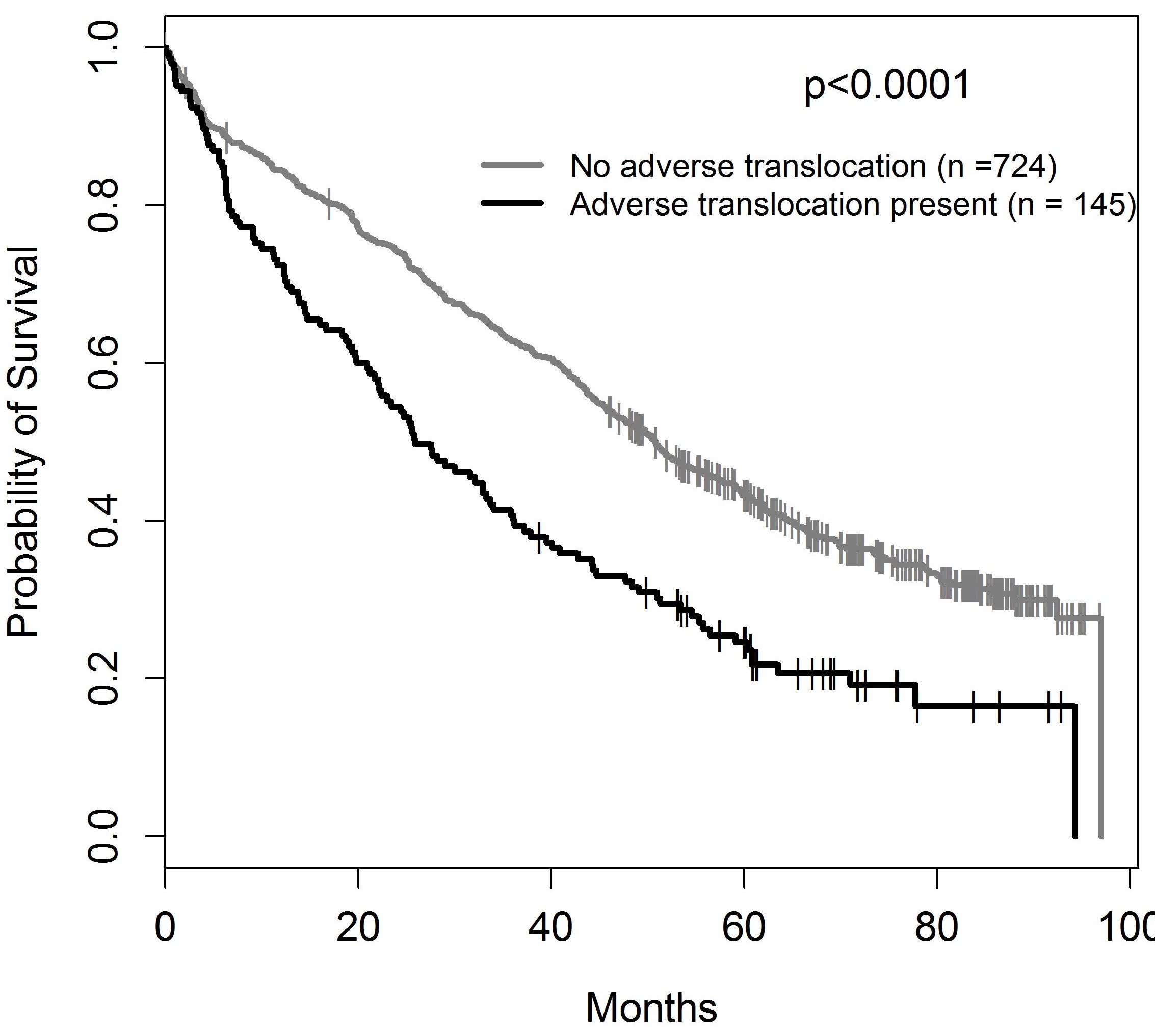 | e | 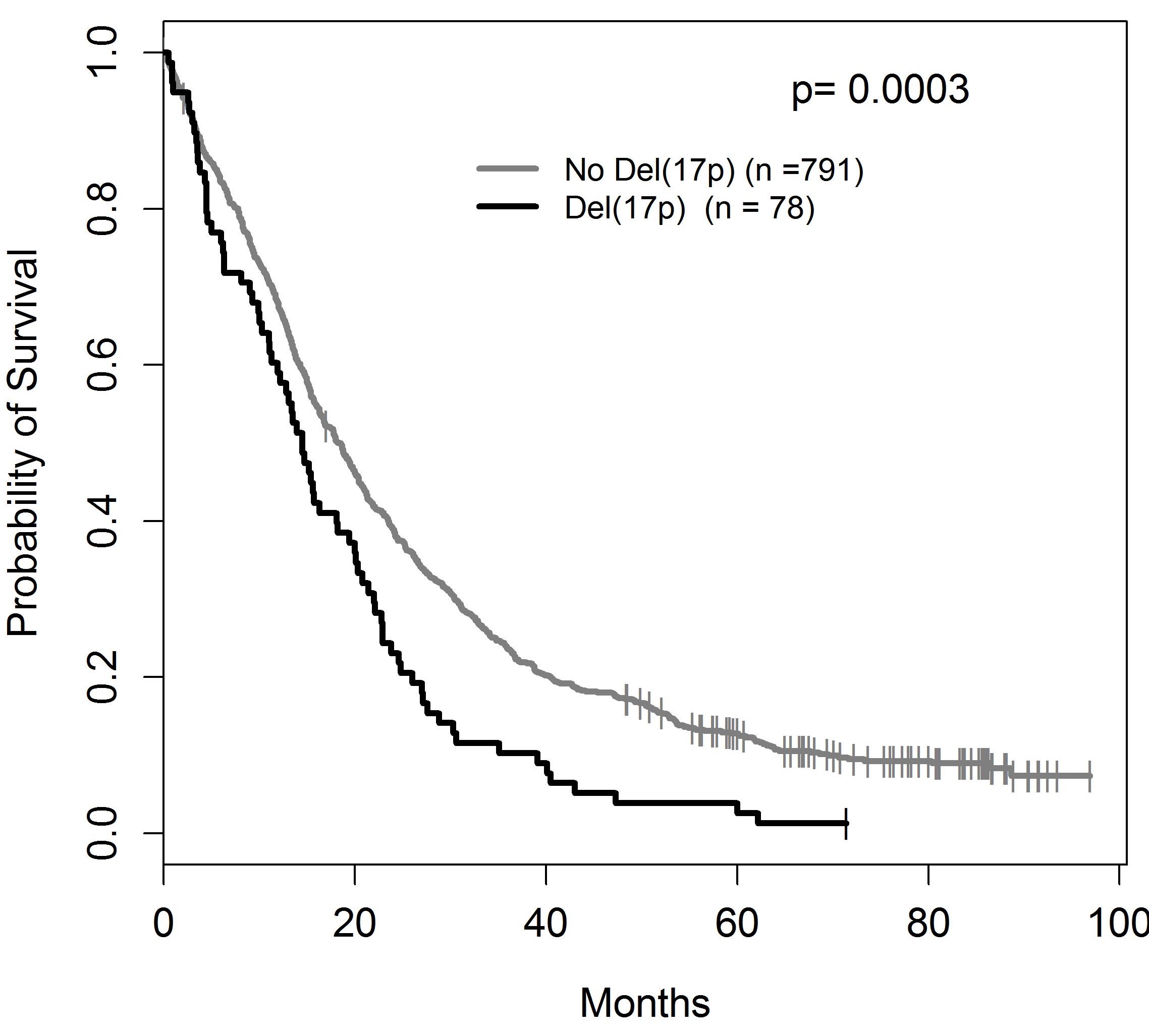 | 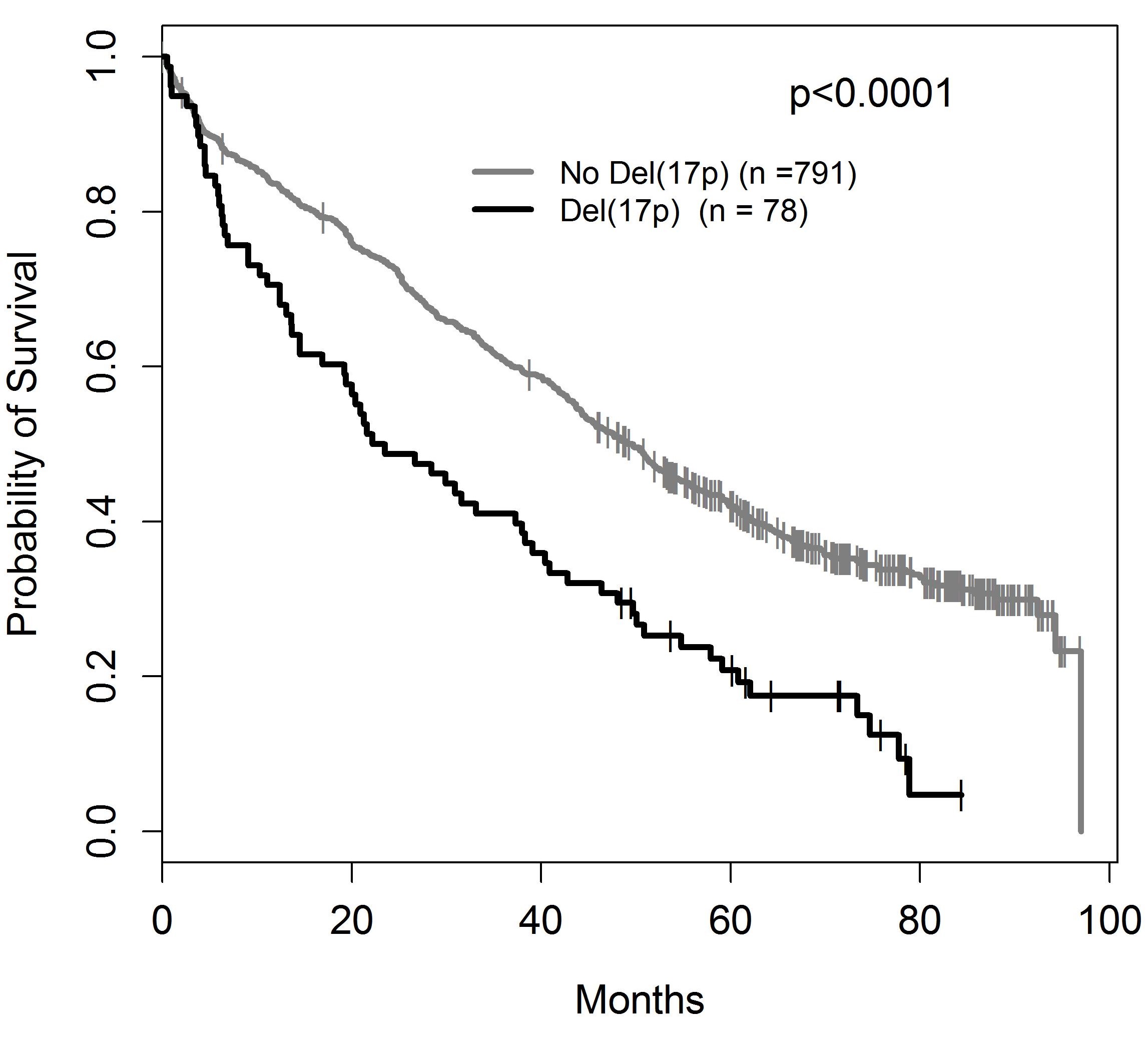 |
| b | 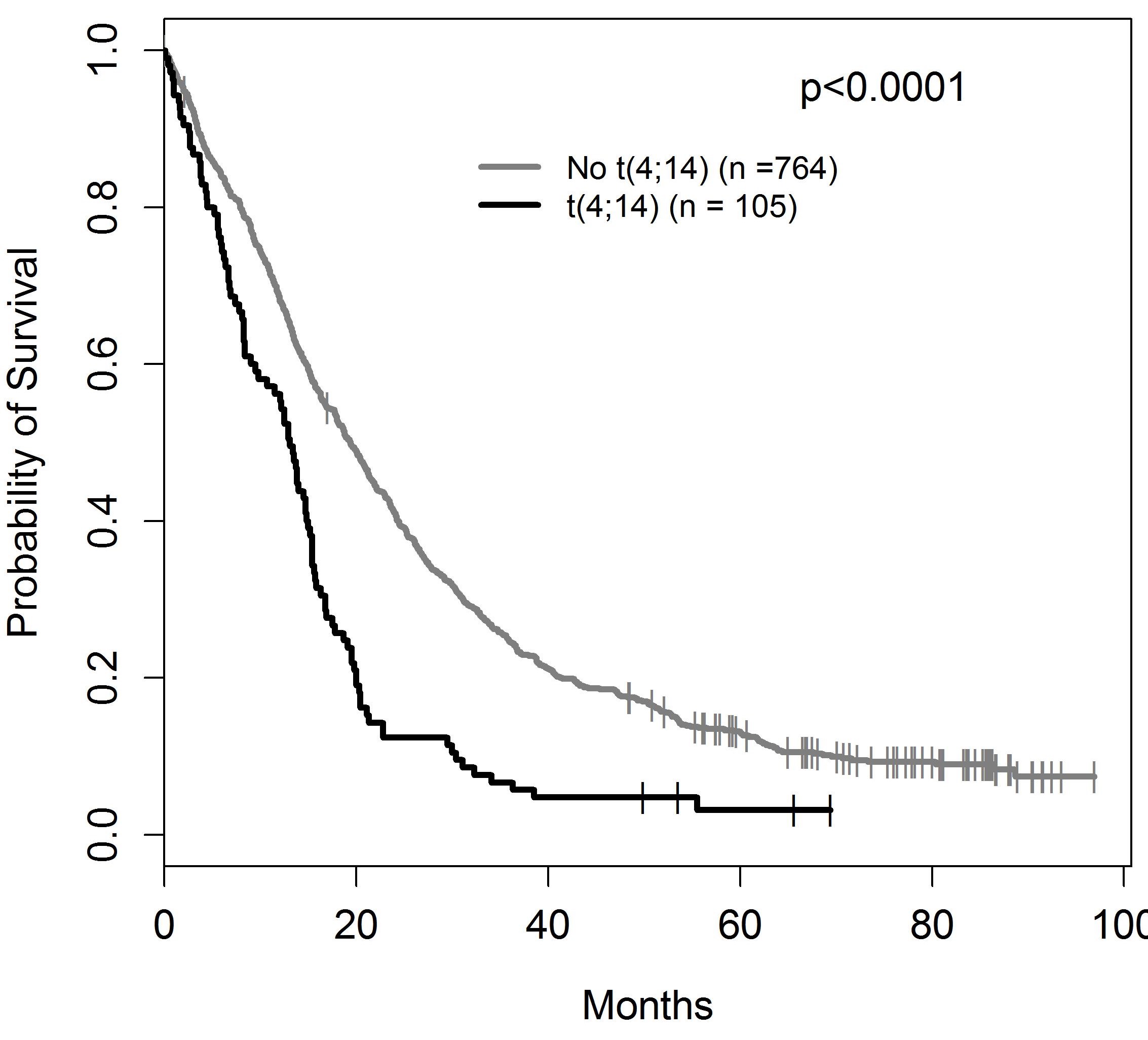 | 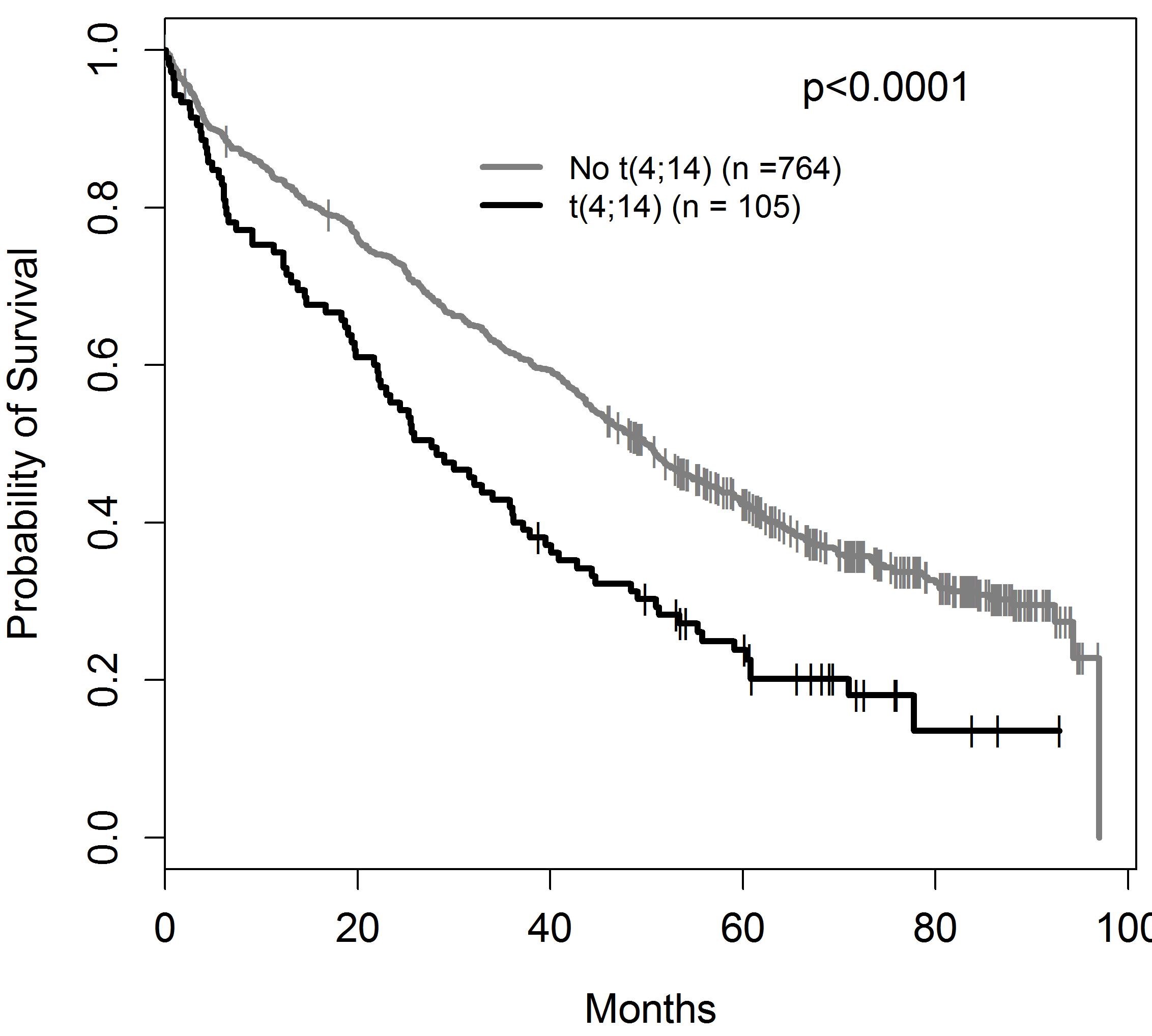 | f | 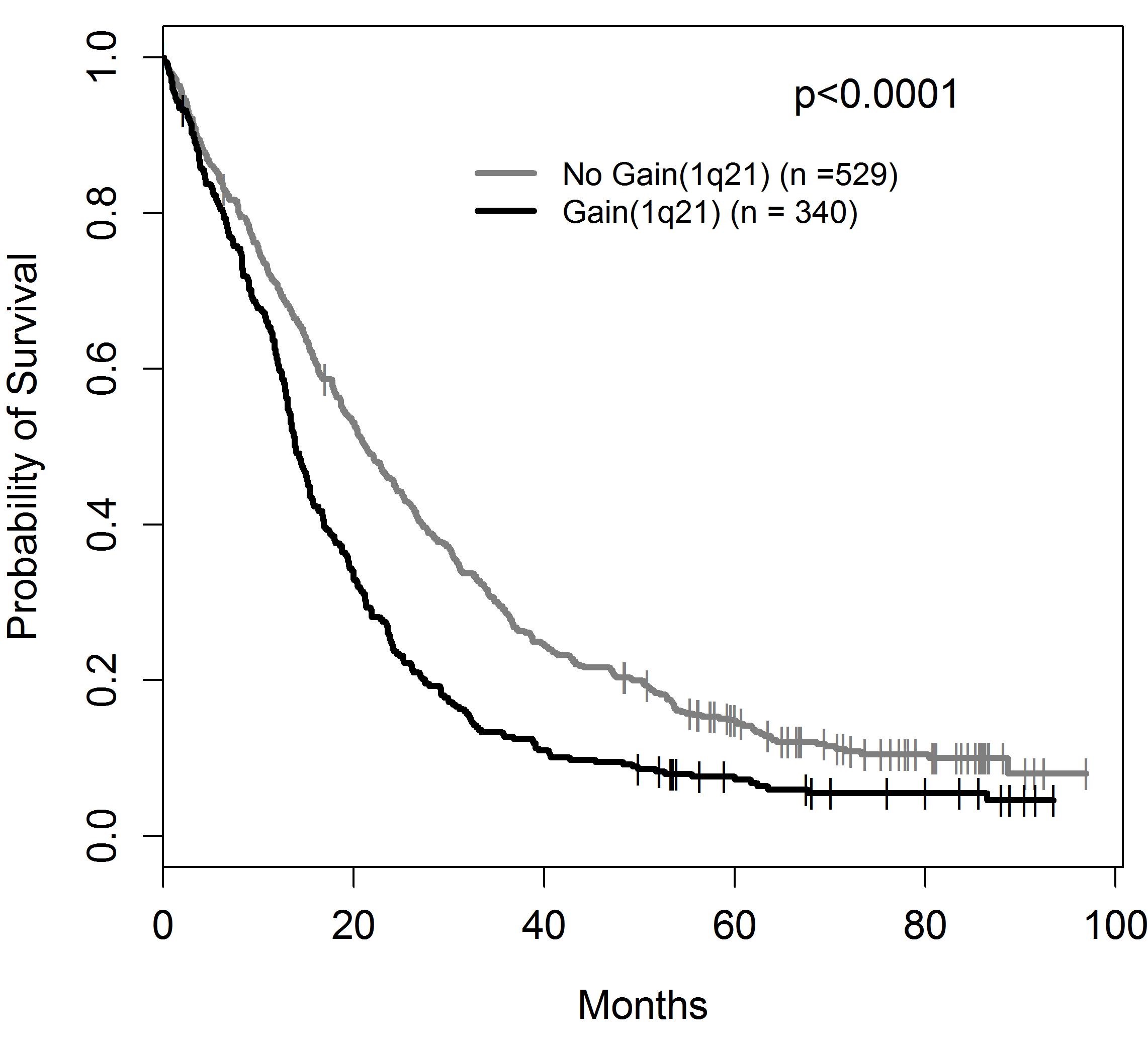 | 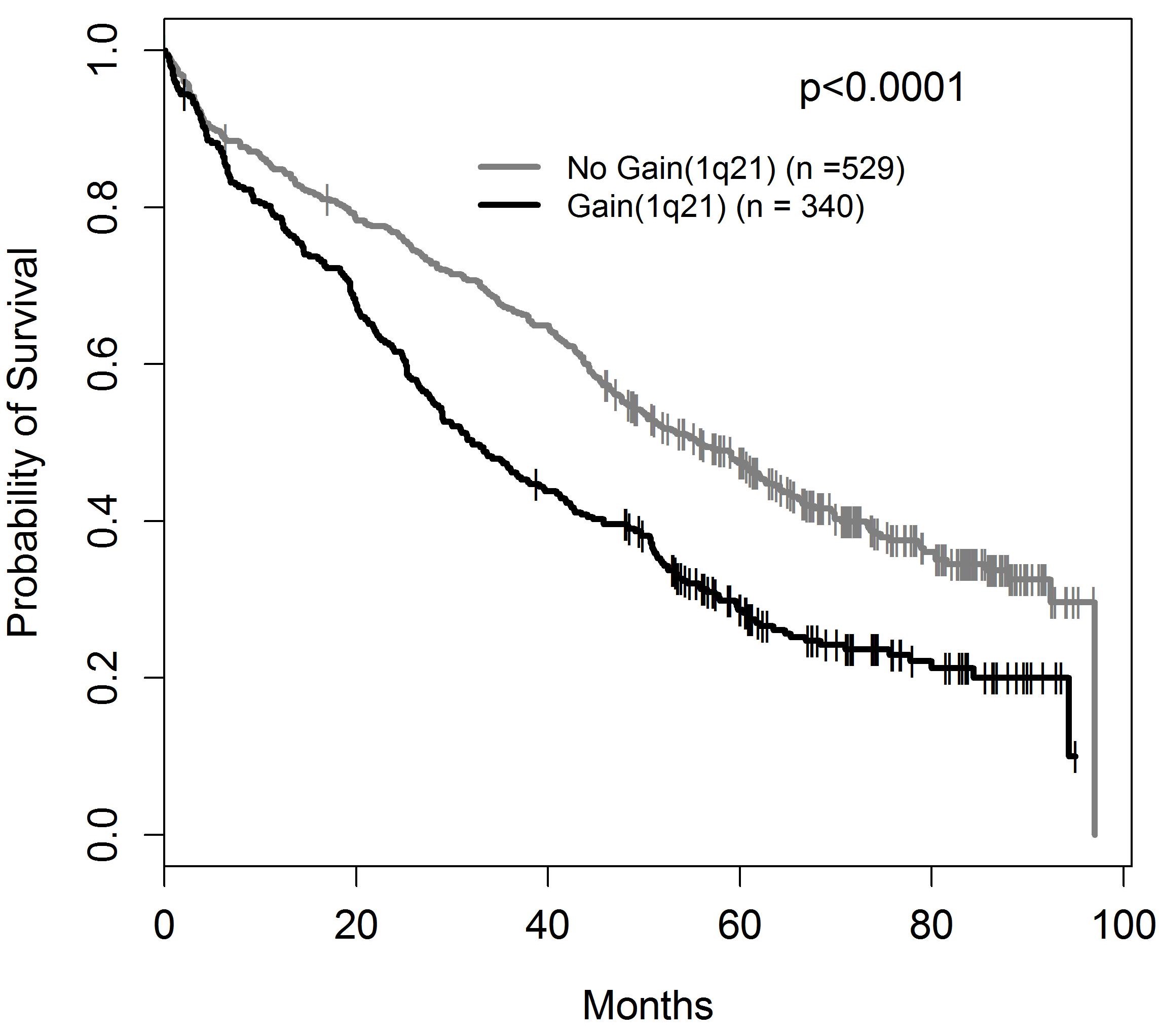 |
| c | 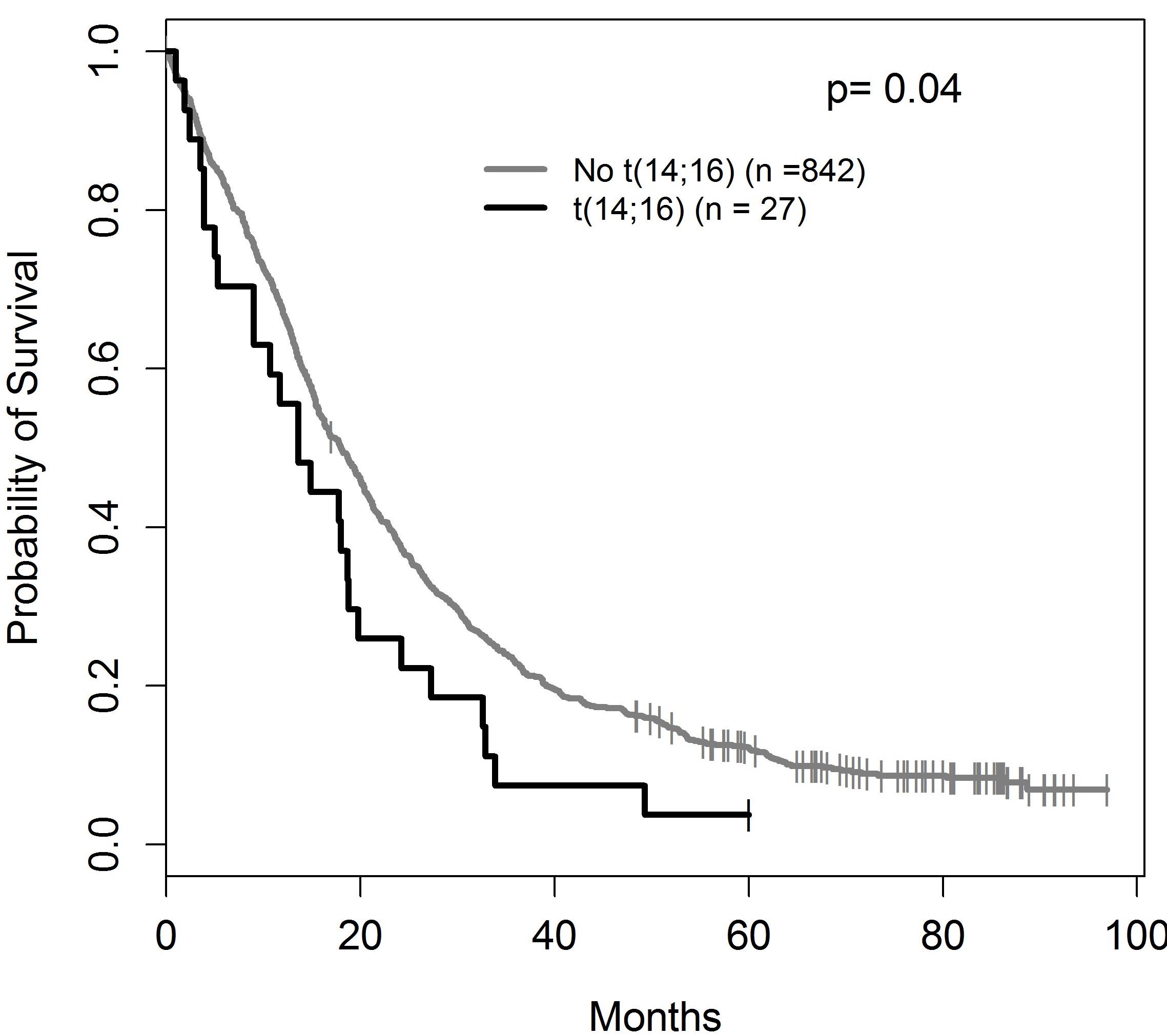 | 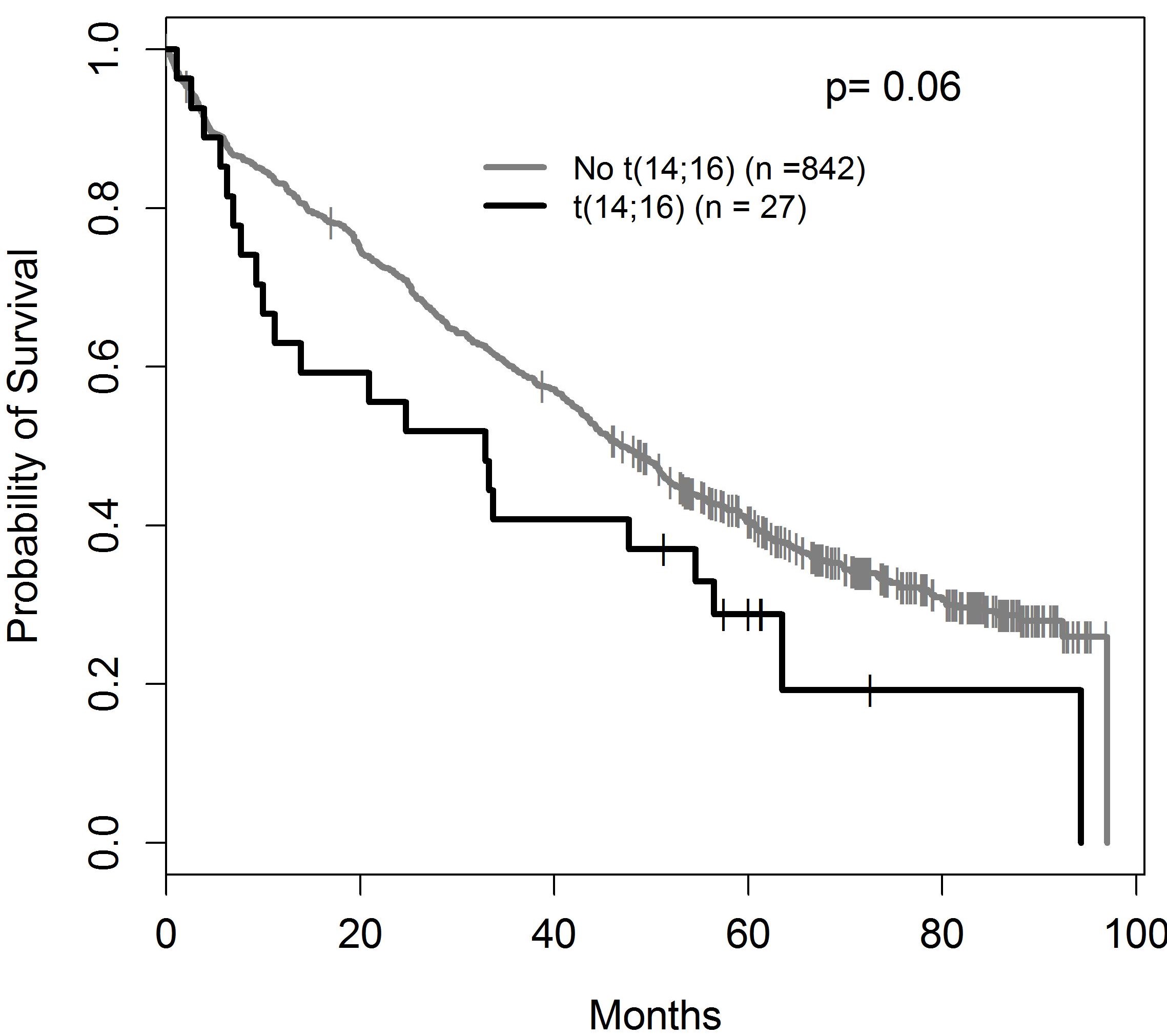 | g | 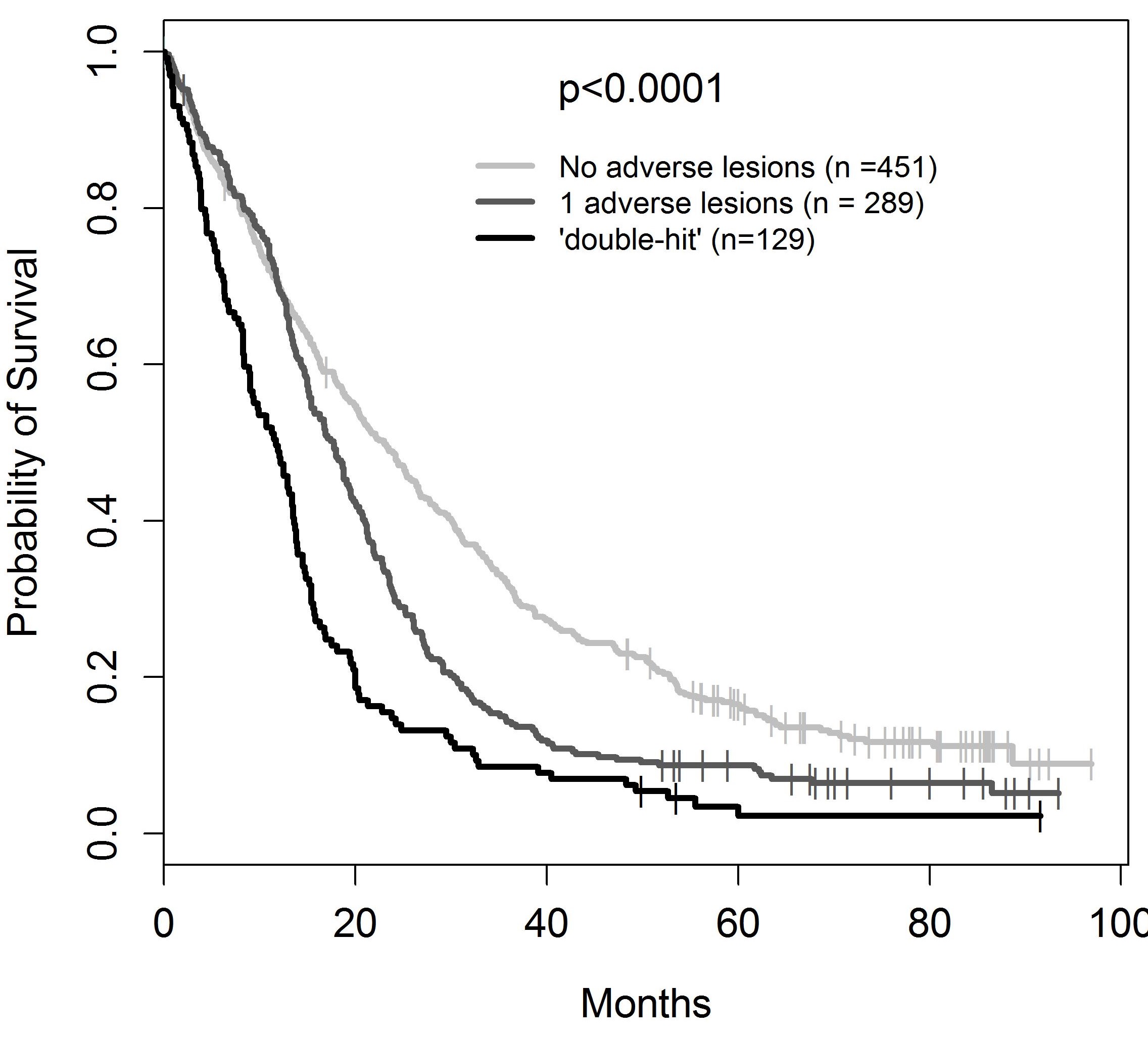 | 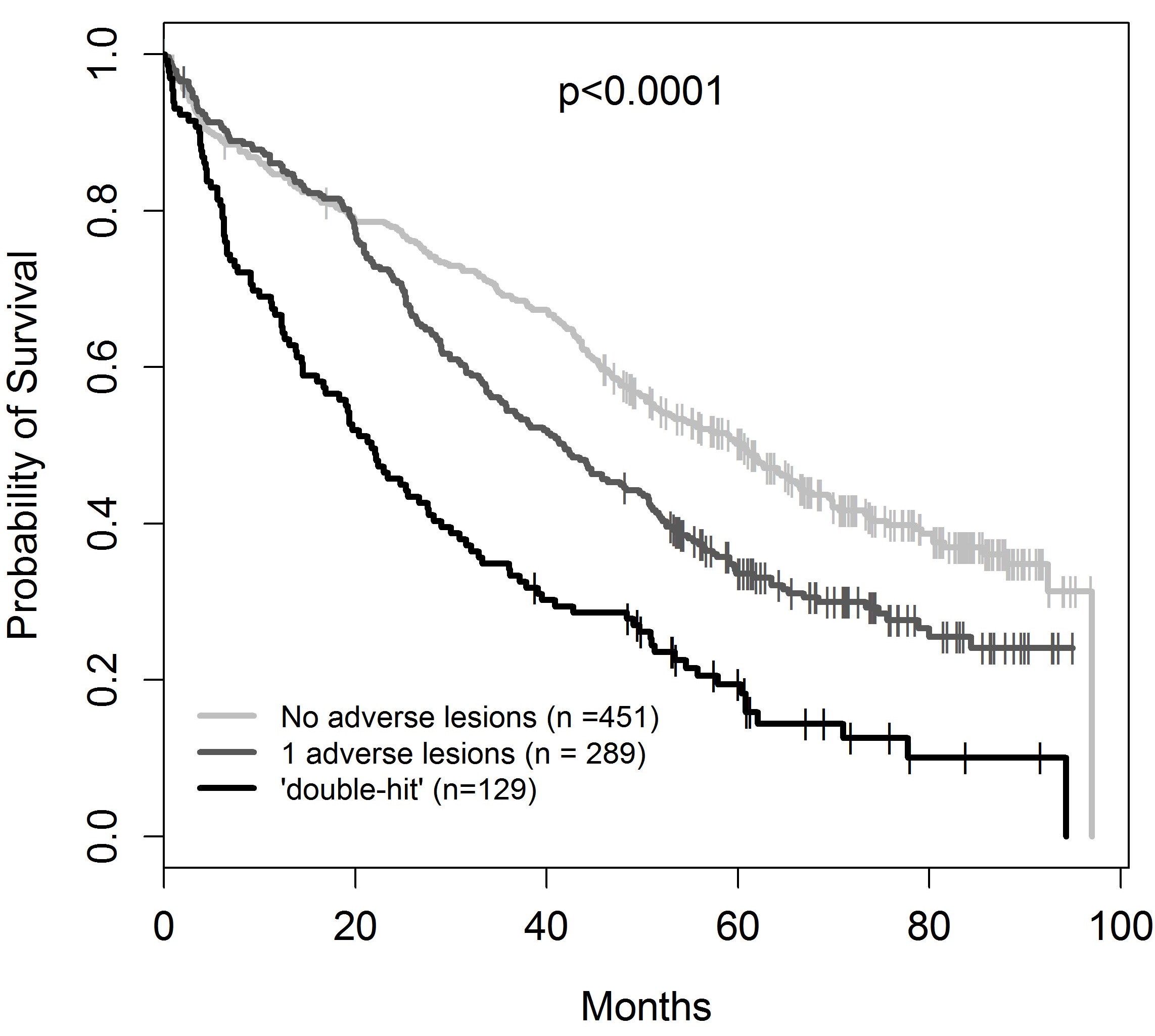 |
| d | 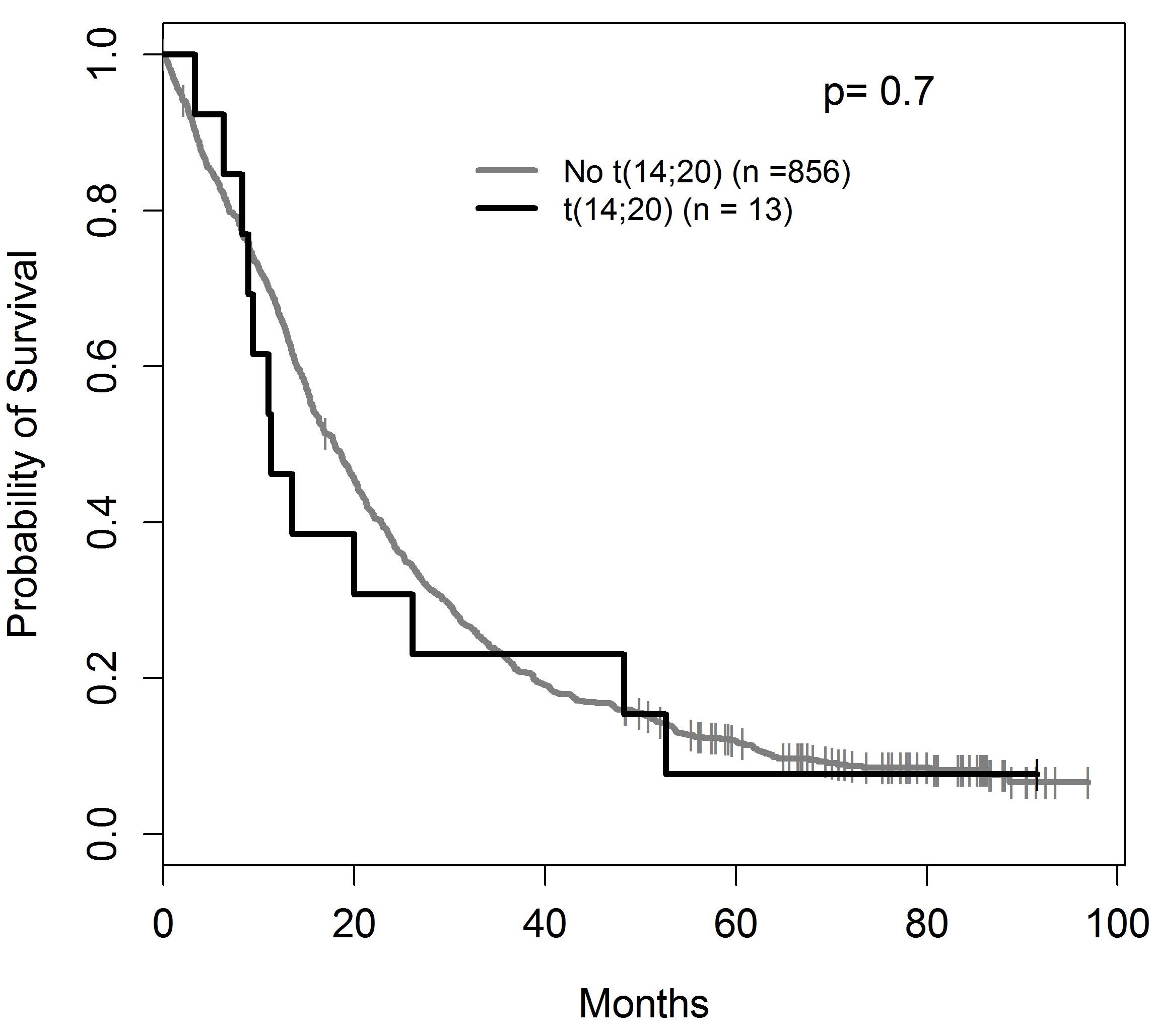 | 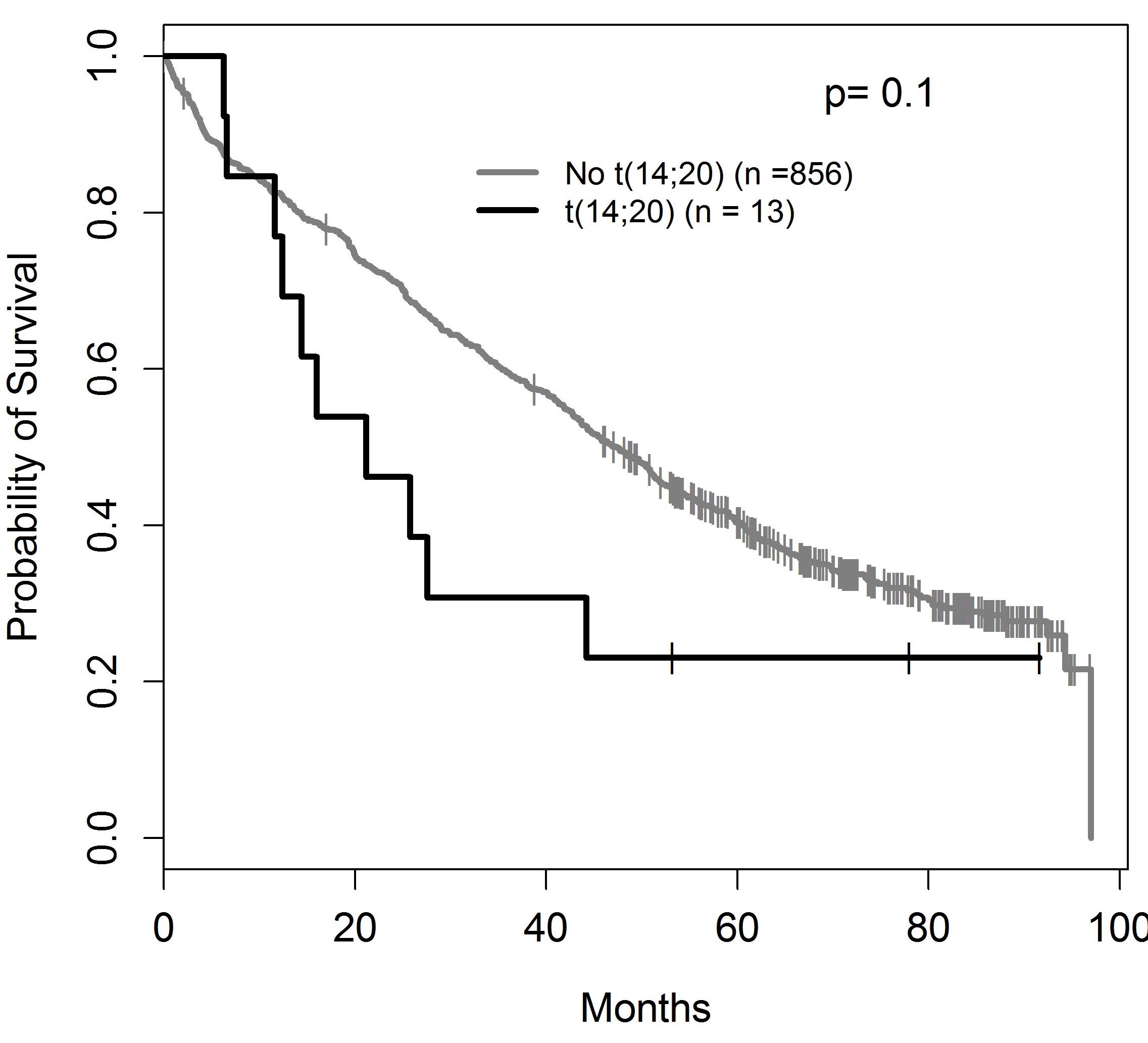 | h | 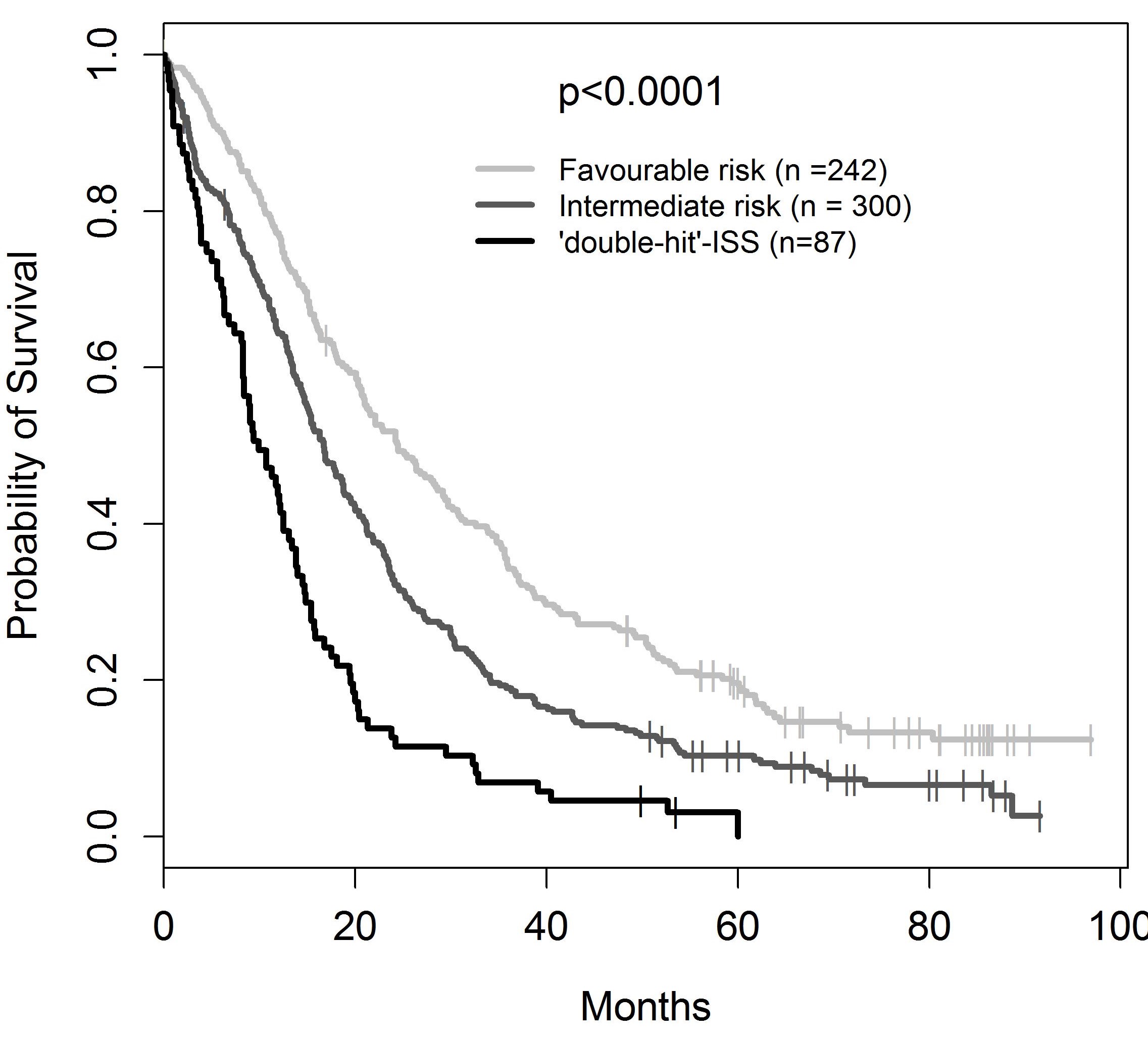 | 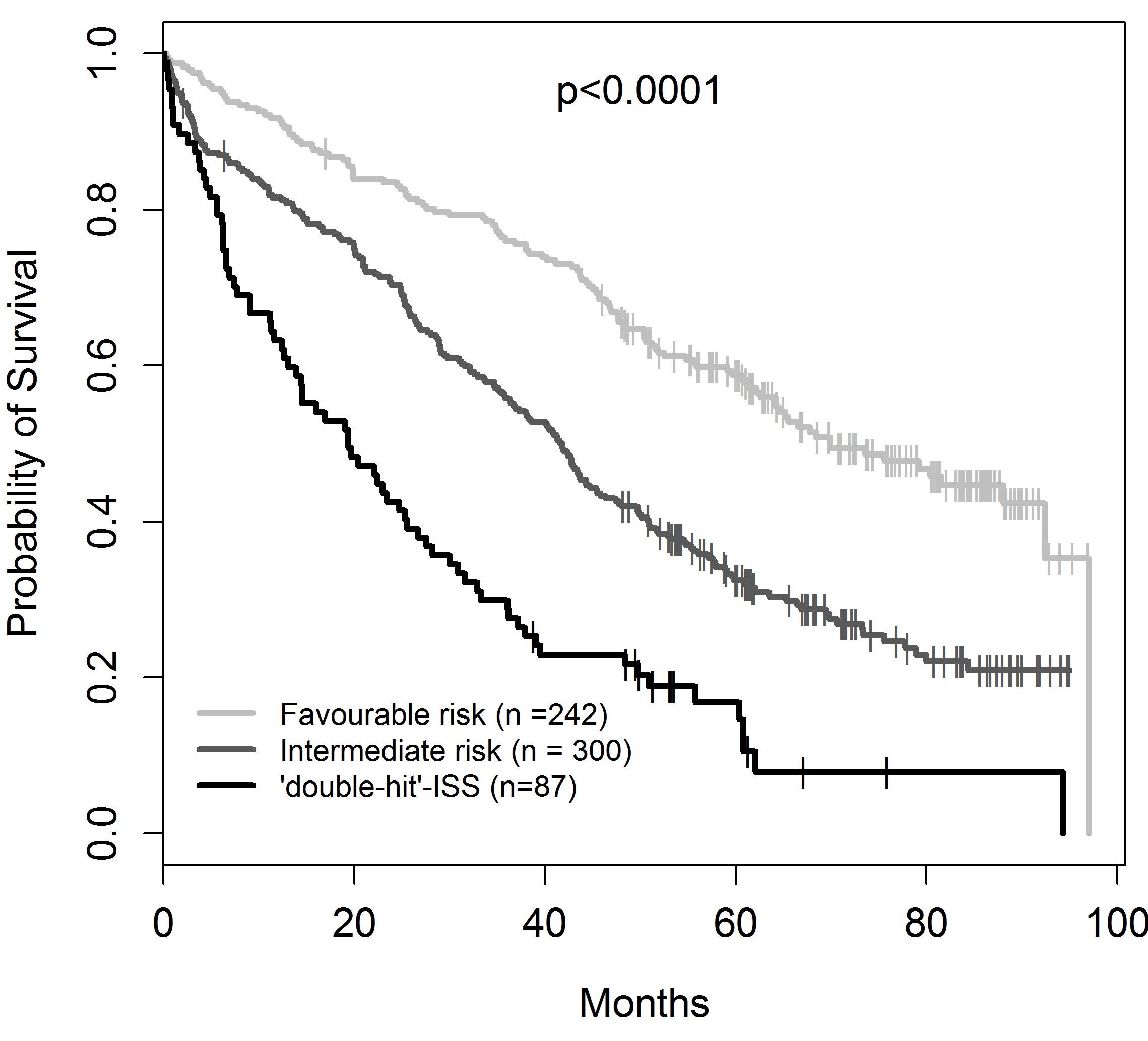 |

**Supplementary Figure 3: Genetic risk markers and survival**

Kaplan-Meier curves and log-rank p-values for 598 intensively treated NCRI Myeloma XI patients in the context of presence of absence of recurrent genetic aberrations.

1. Adverse Translocation
2. t(4;14)
3. t(14;16)
4. t(14;20)
5. del(17p)
6. gain(1q)
7. ‘double-hit’
8. ‘double-hit’-ISS

|  | PFS | OS |  | PFS | OS |
| --- | --- | --- | --- | --- | --- |
| a | 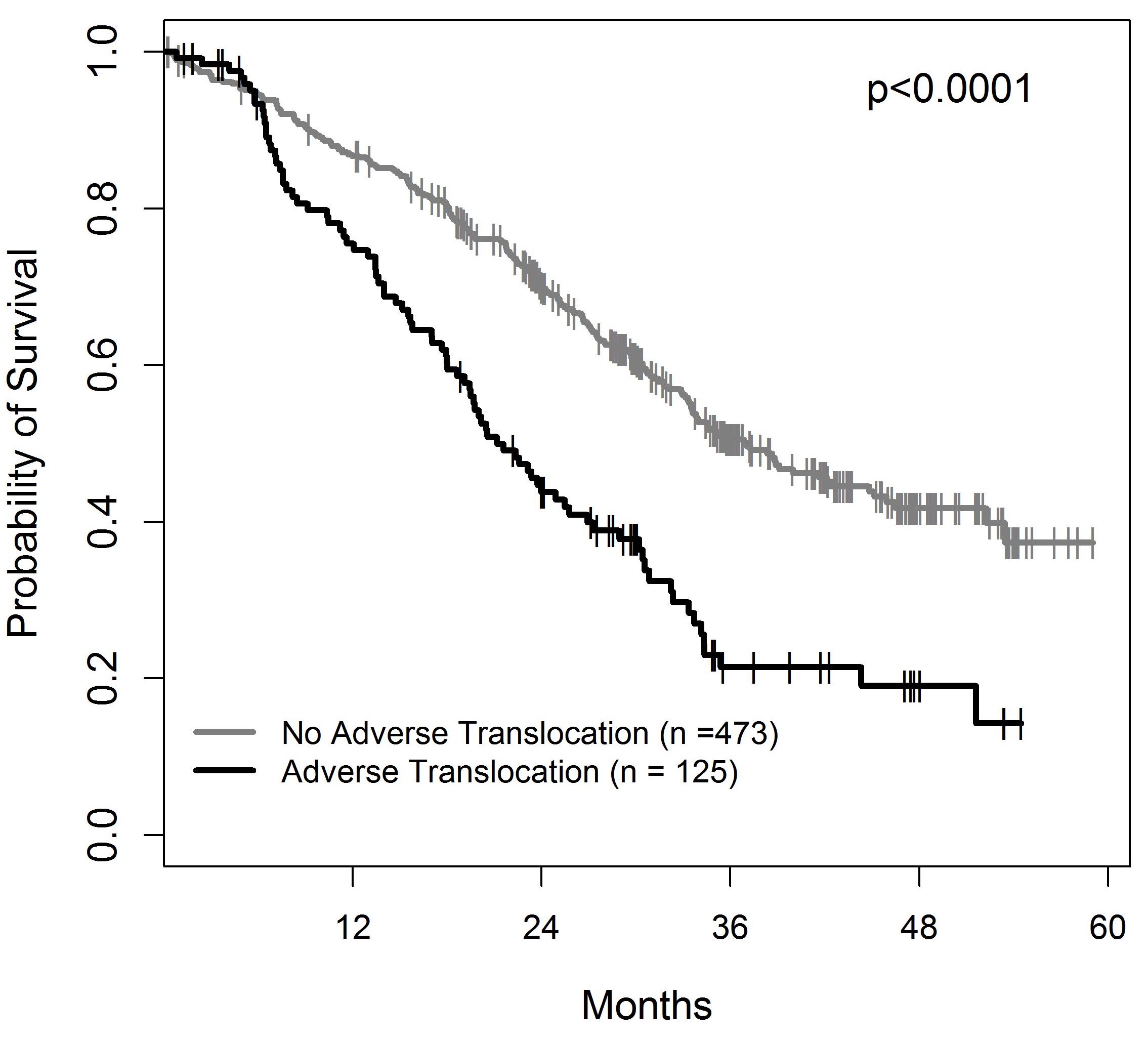 | 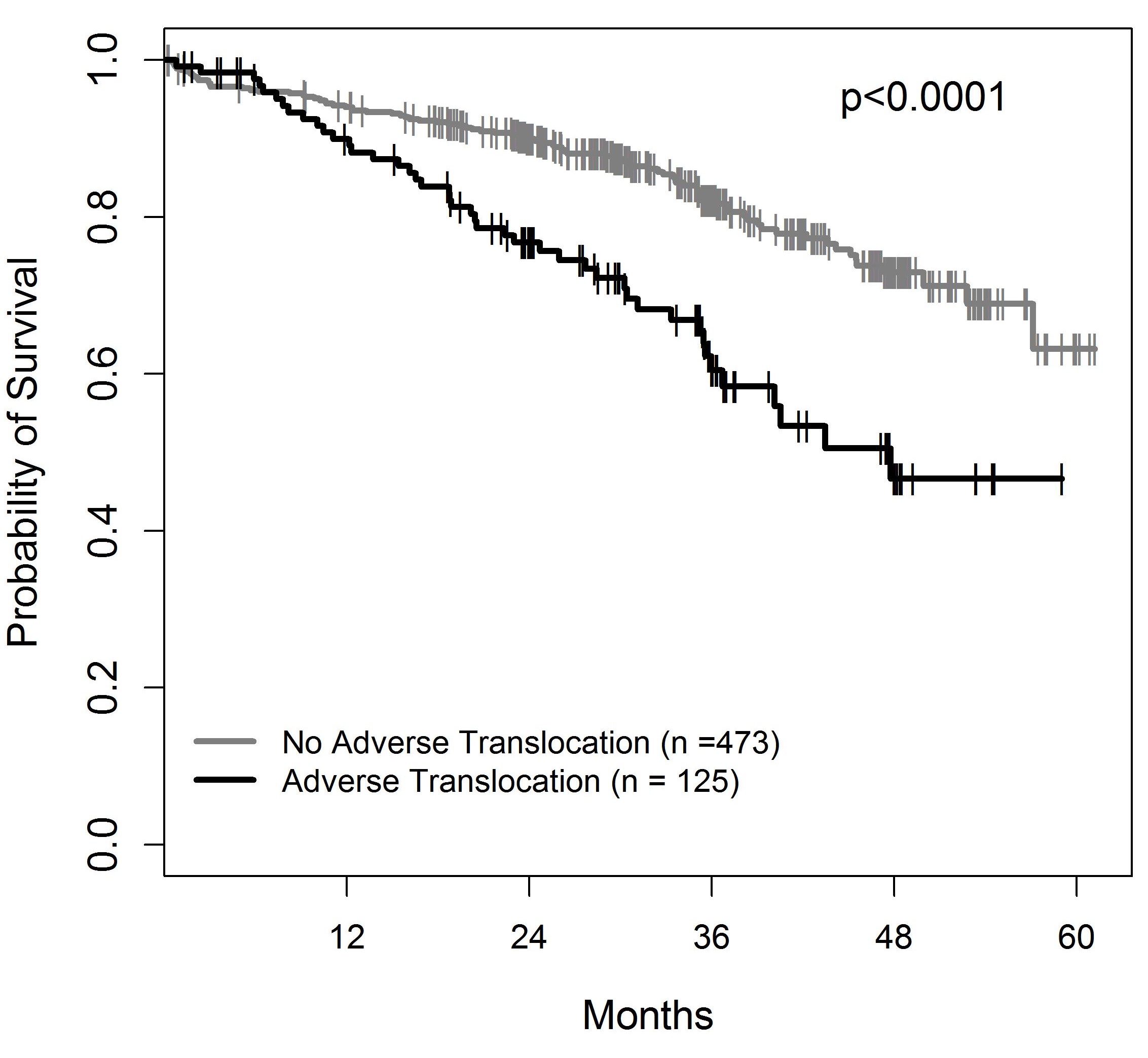 | e | 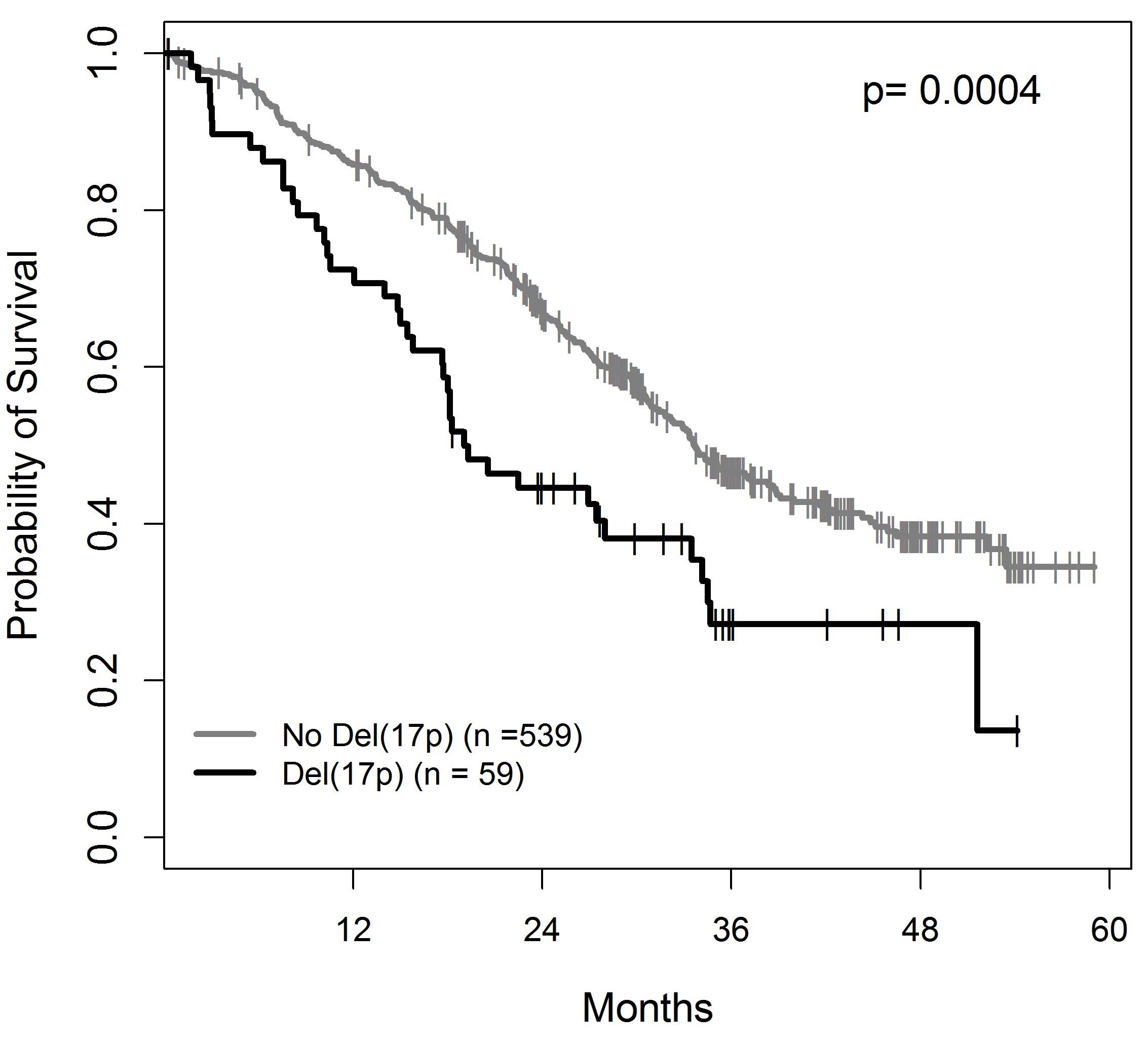 | 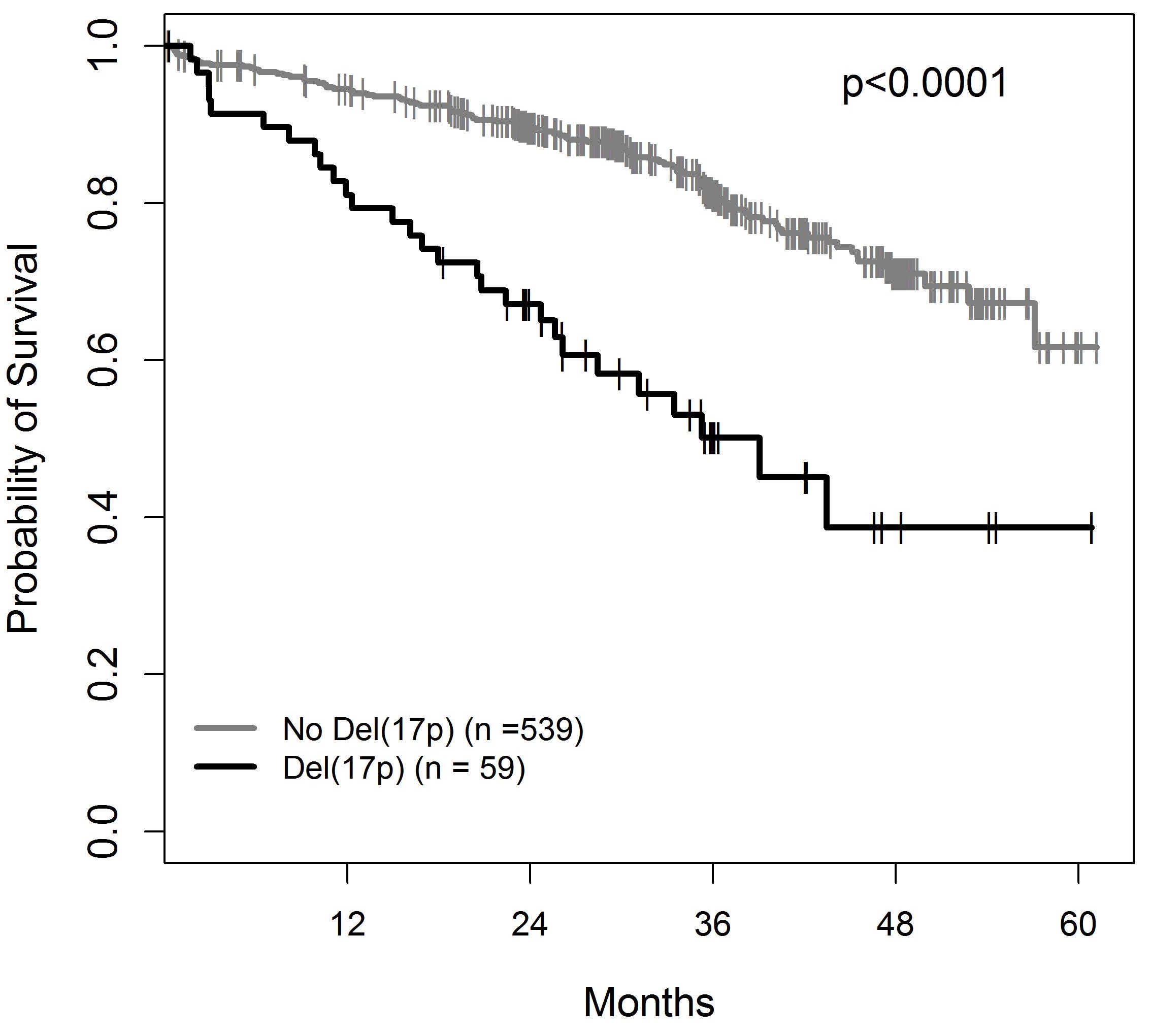 |
| b | 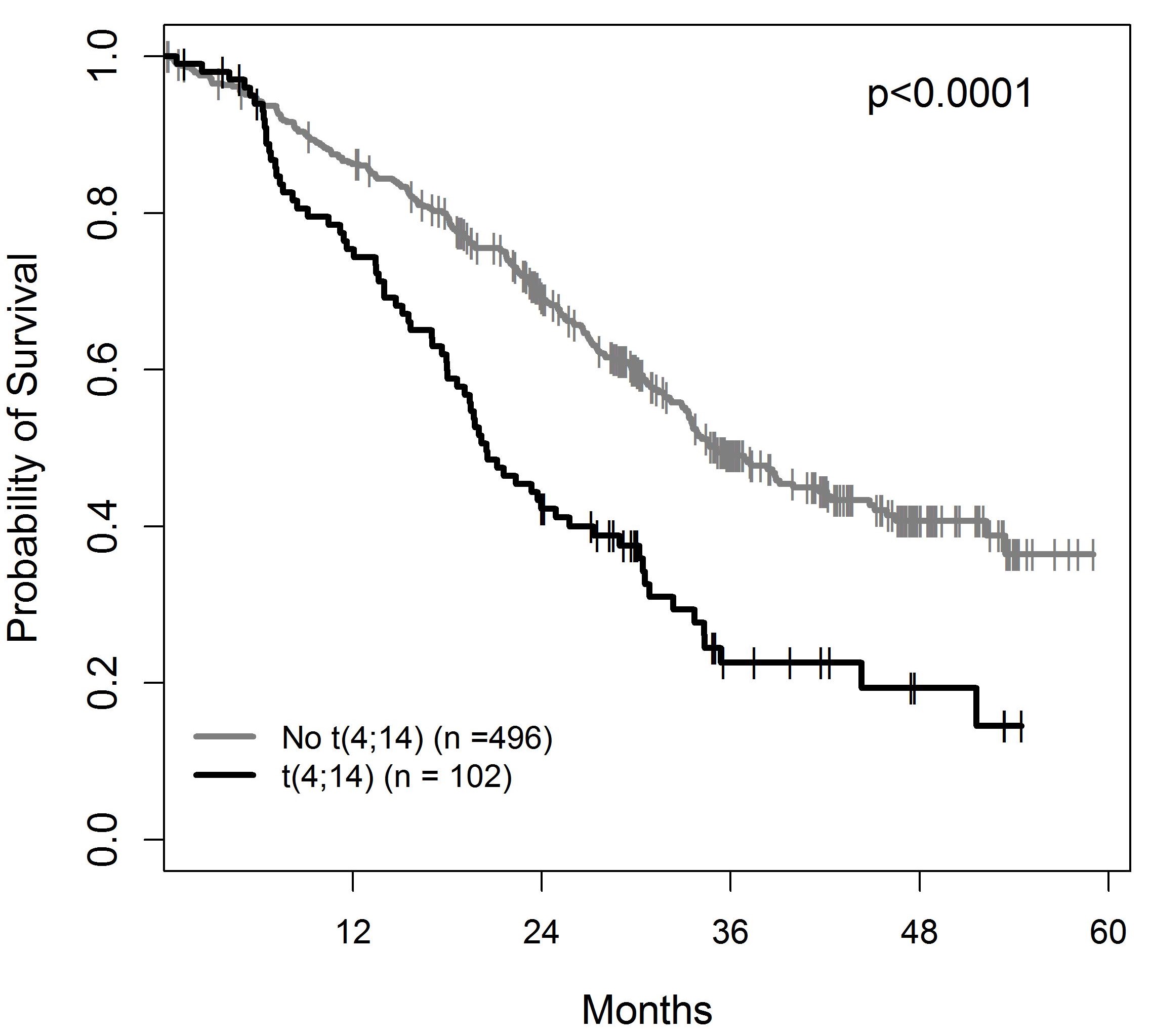 | 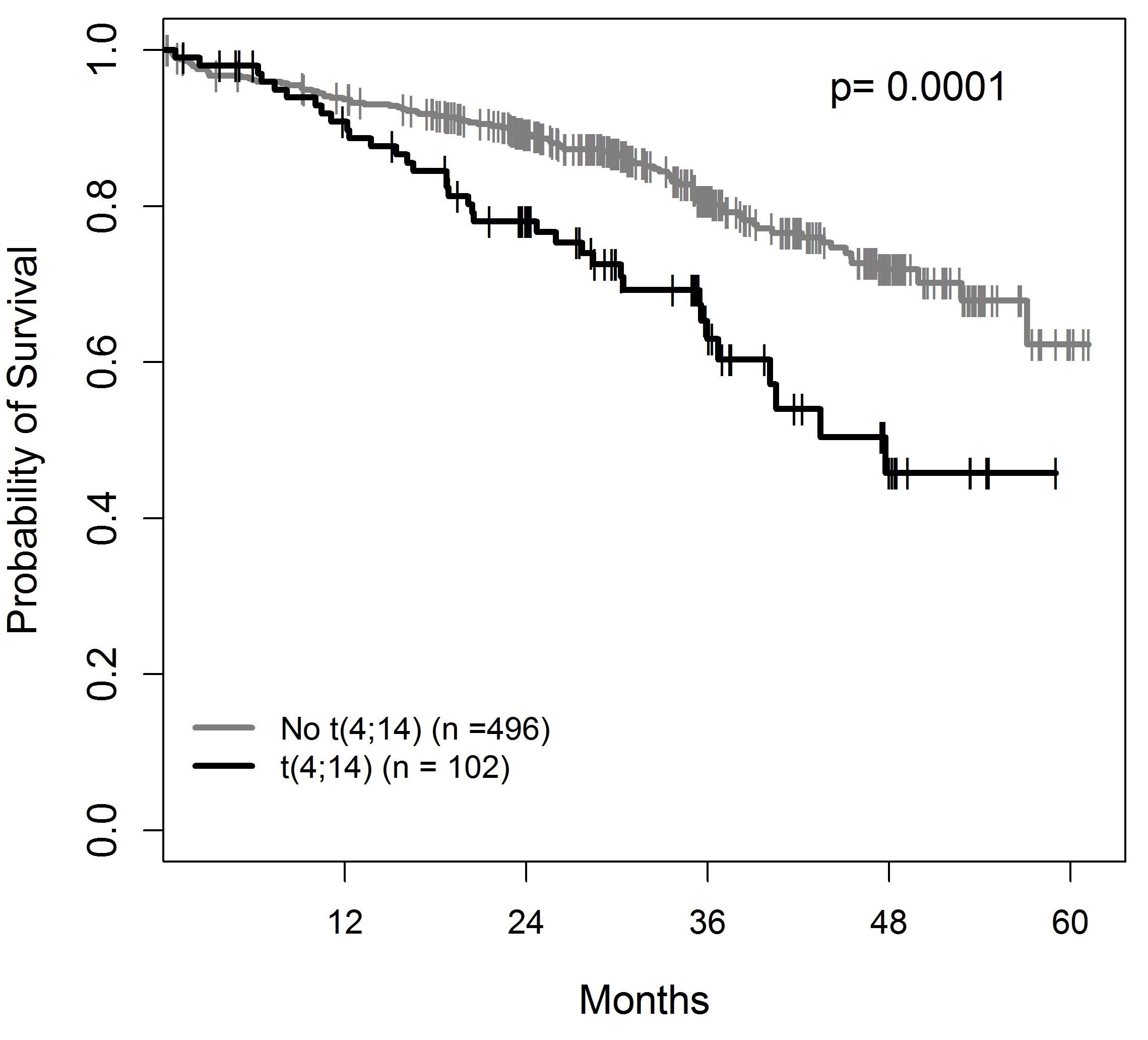 | f | 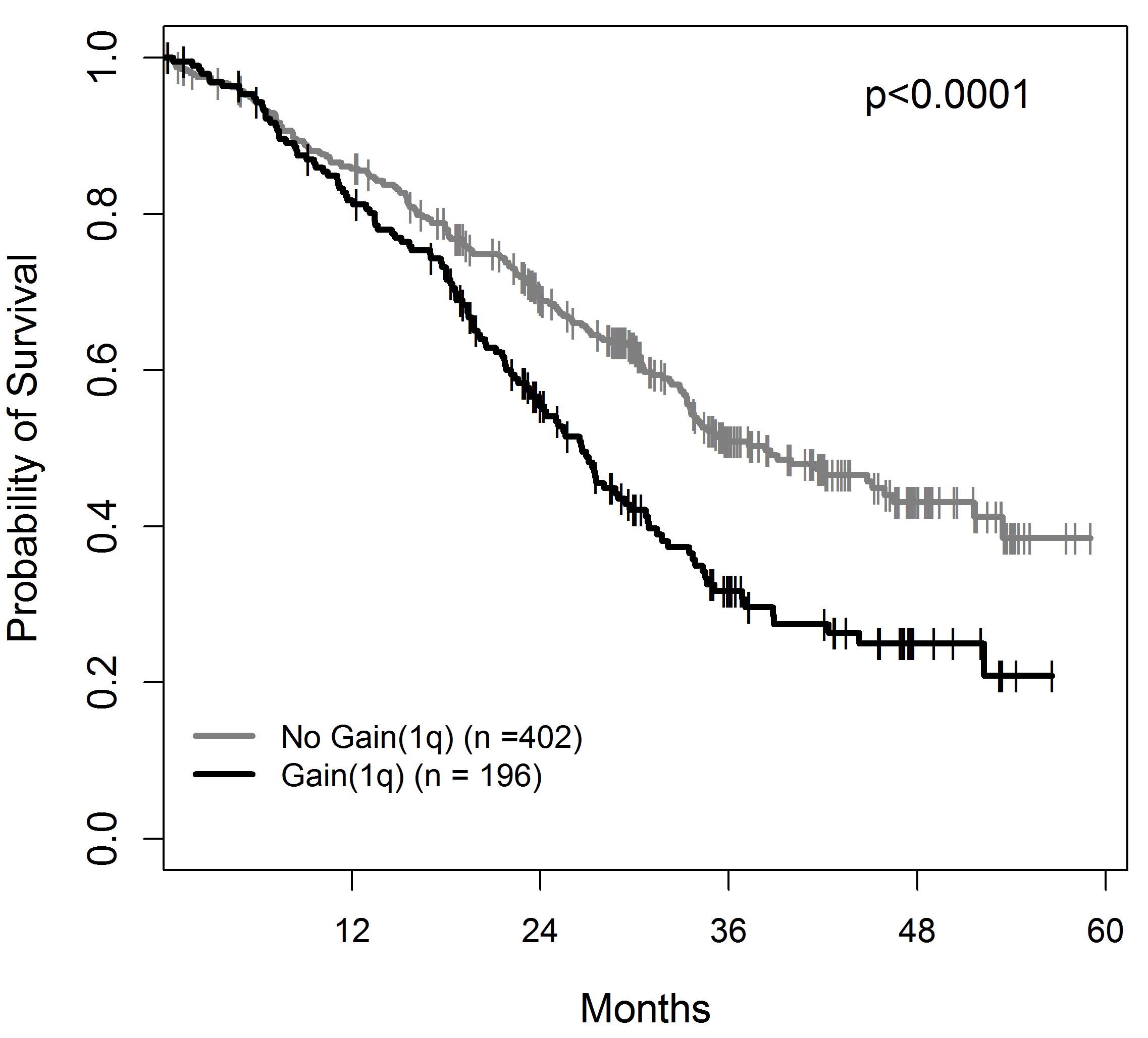 | 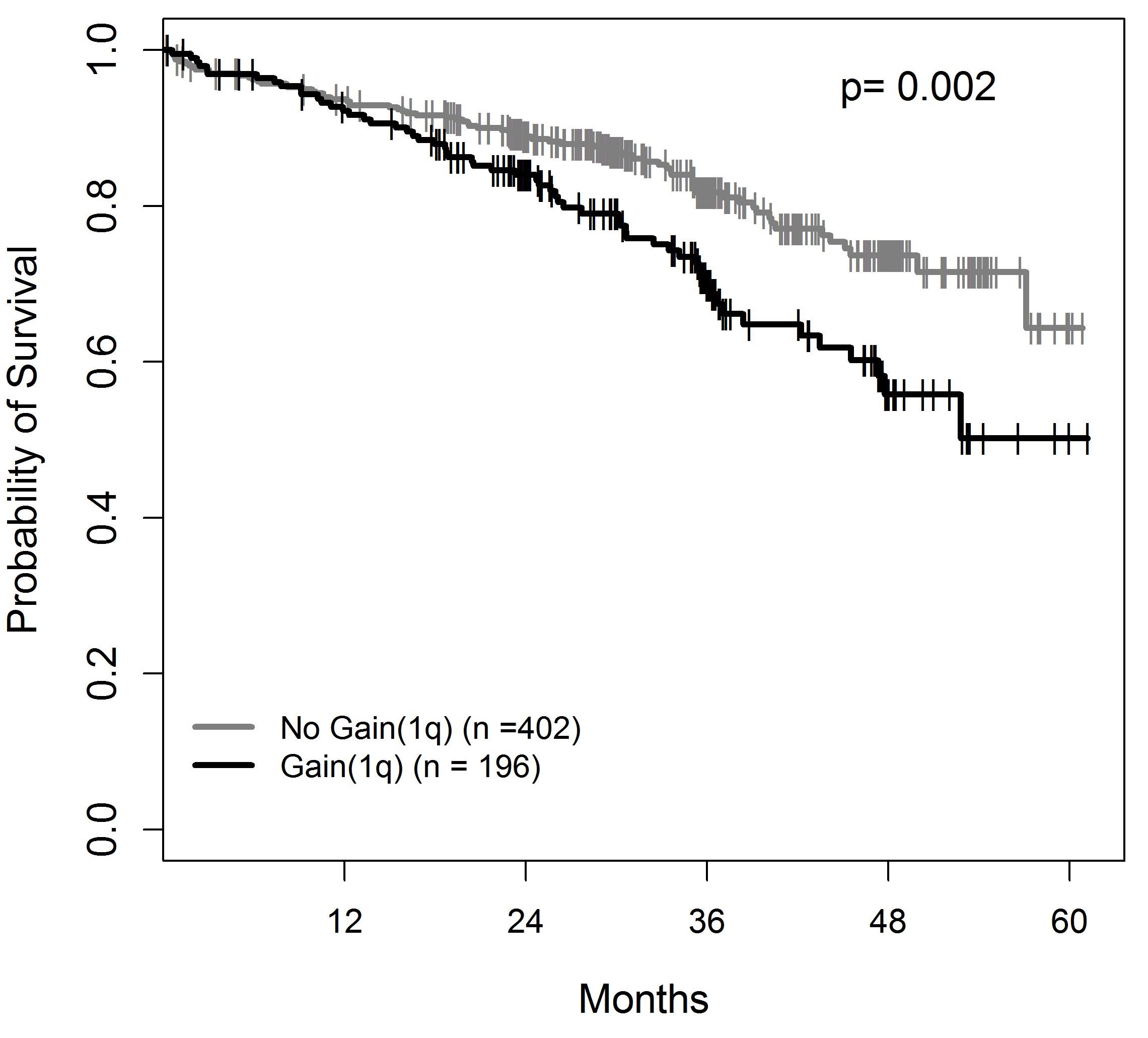 |
| c | 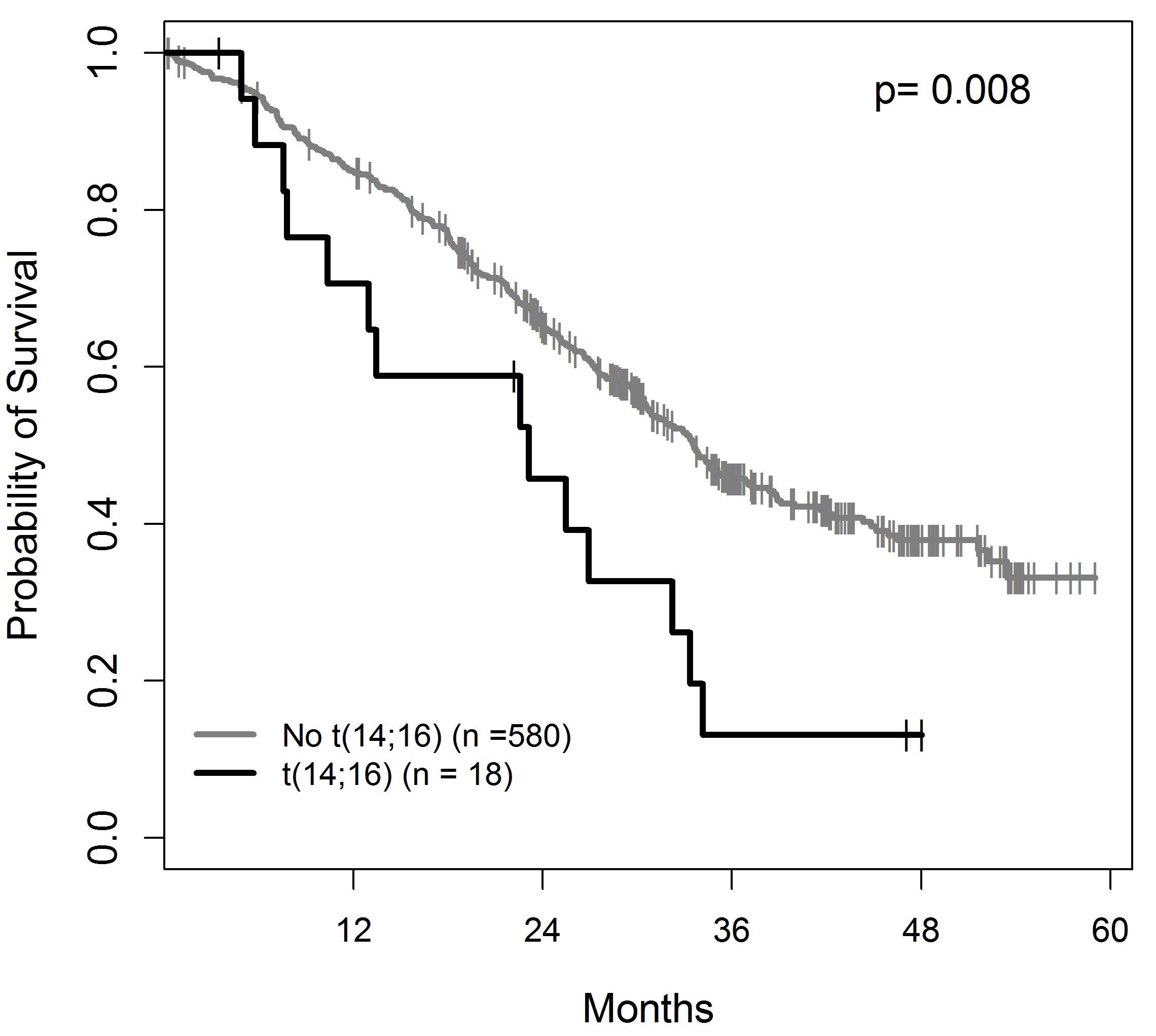 | 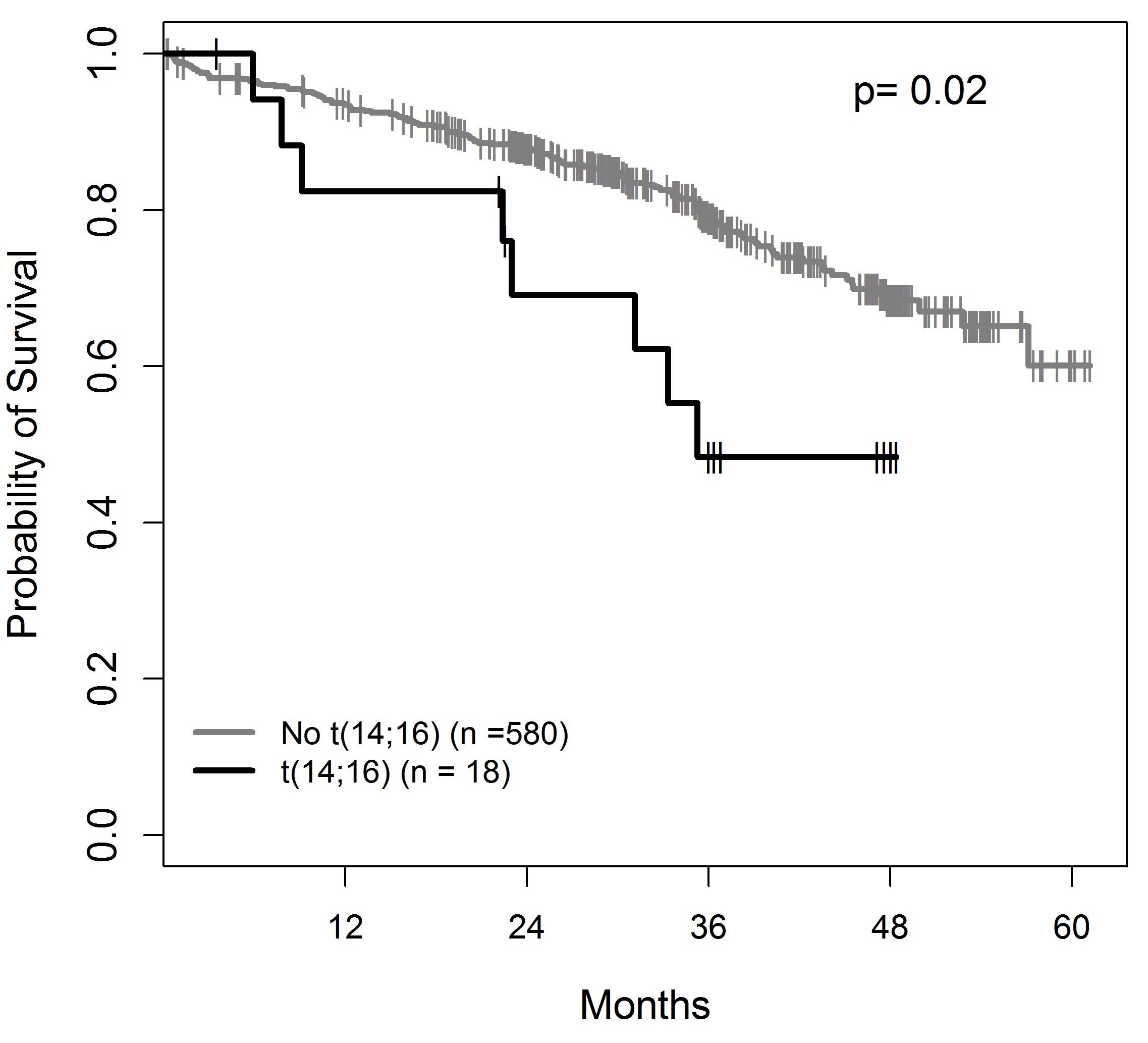 | g | 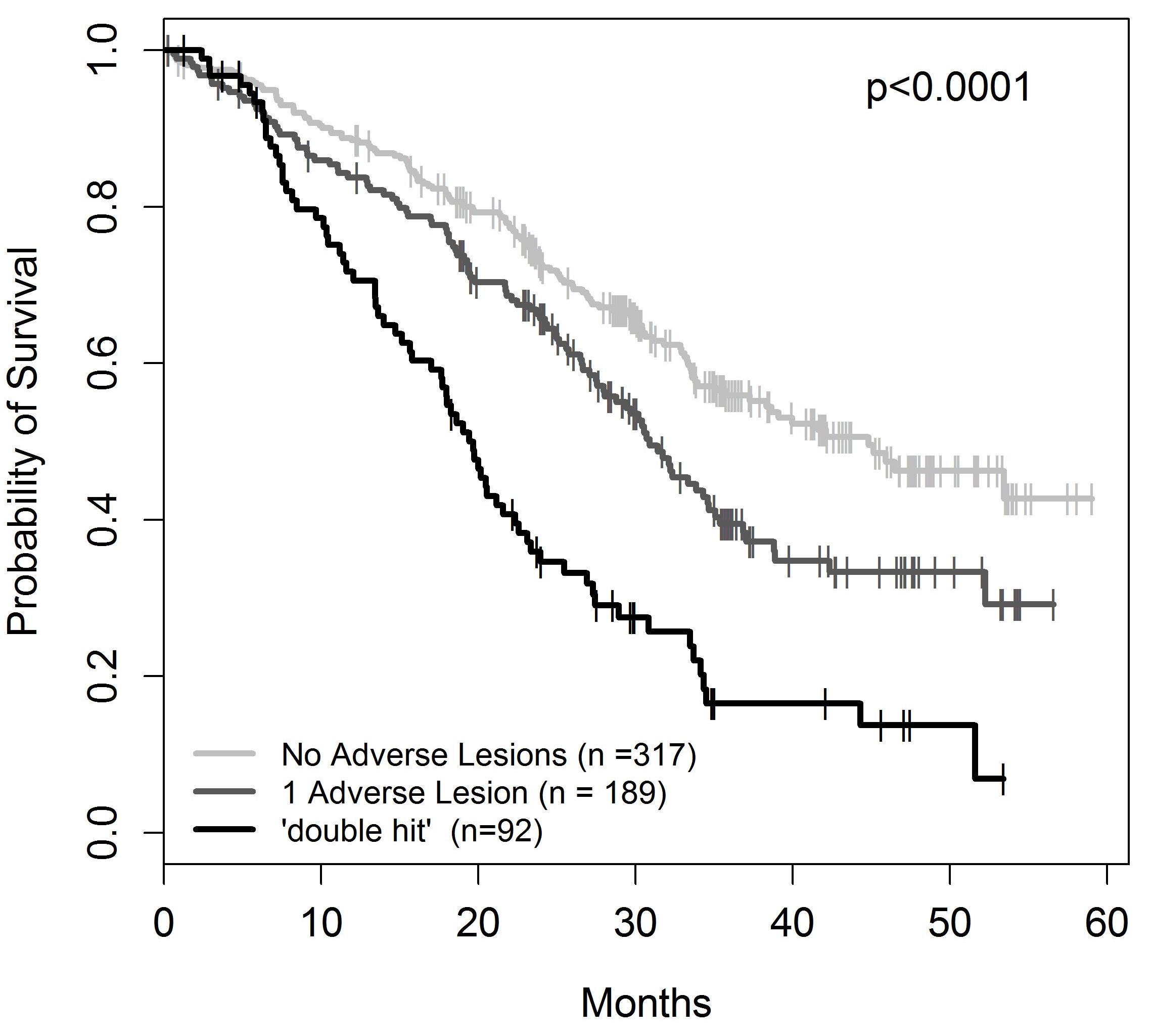 | 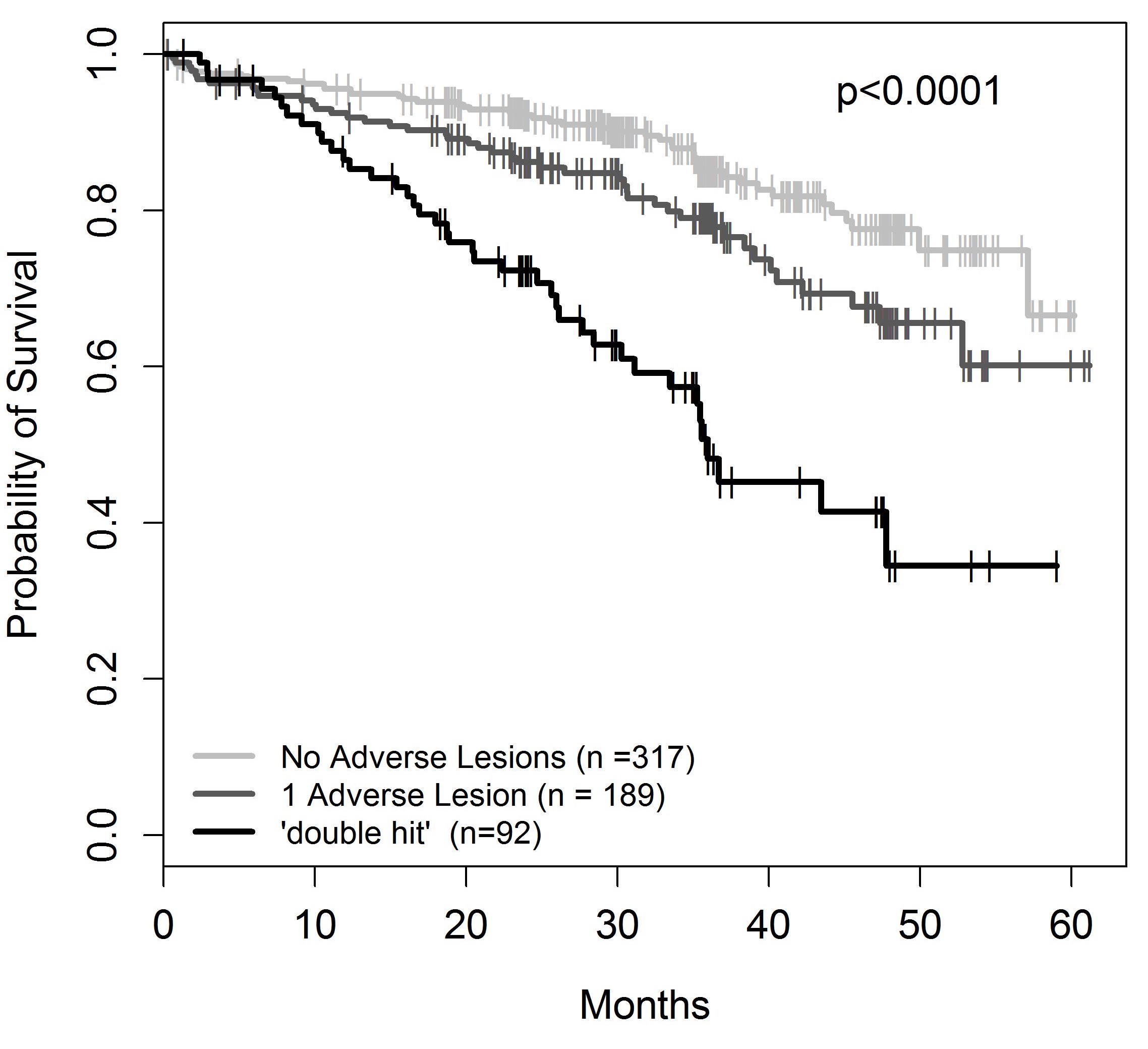 |
| d | 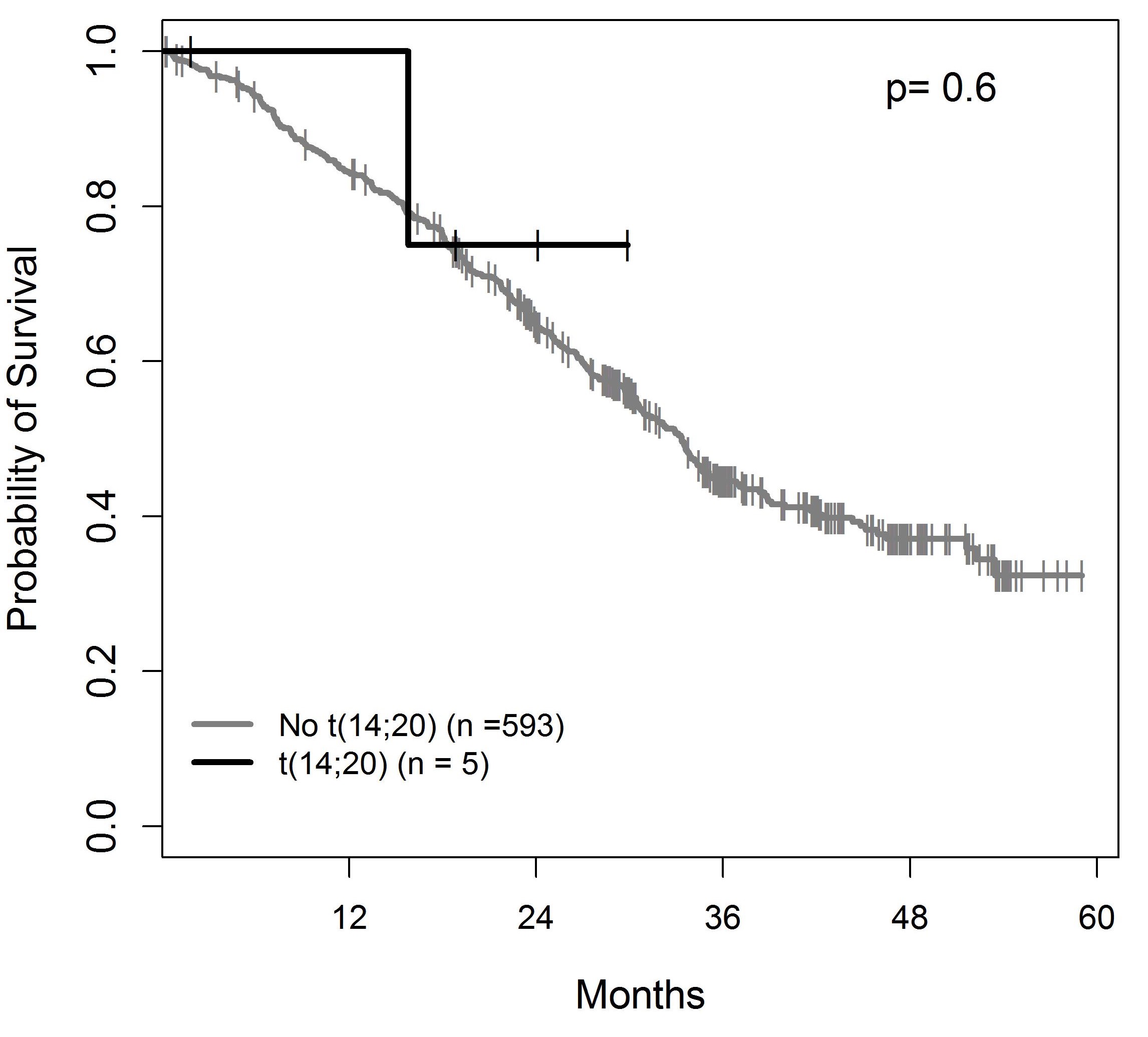 | 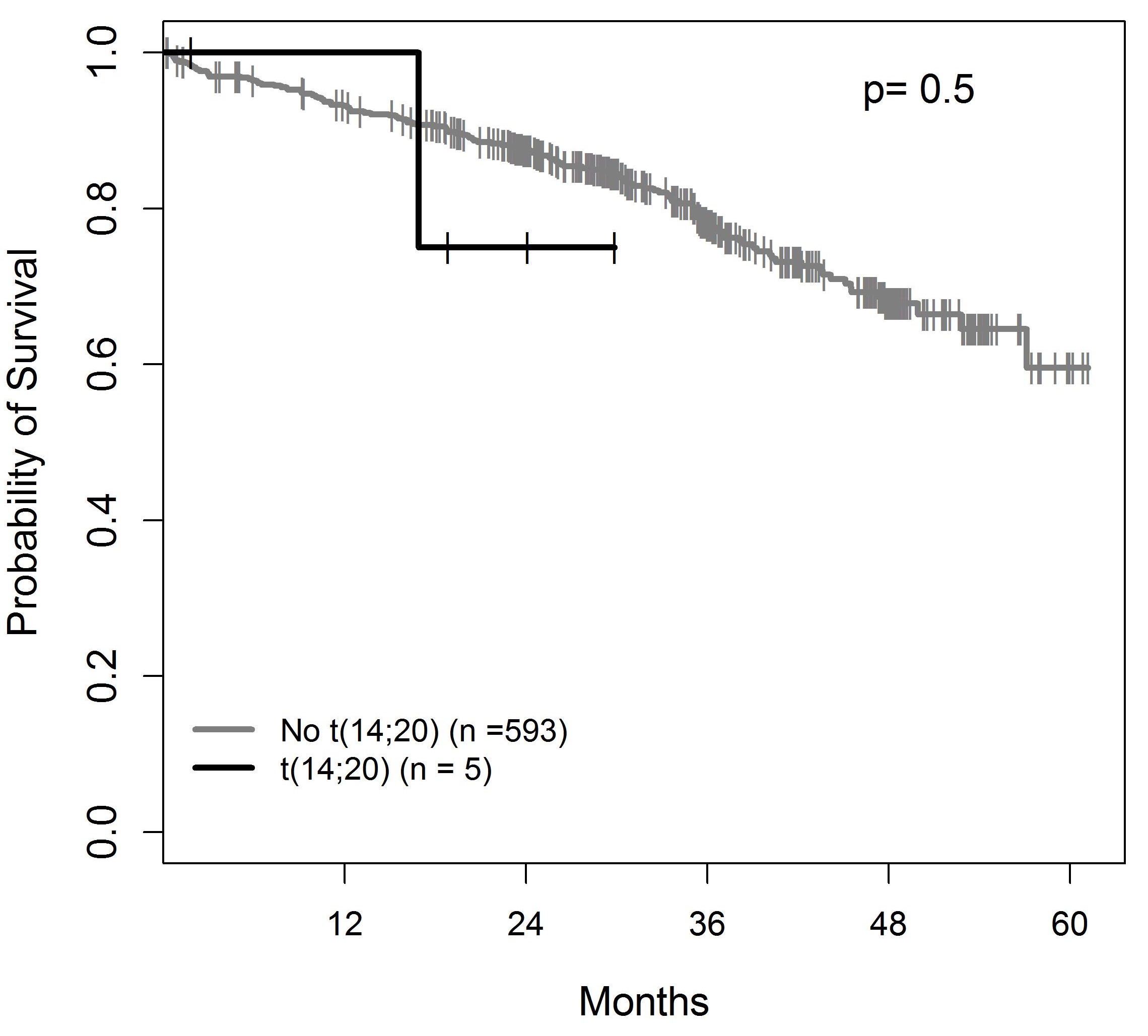 | h | 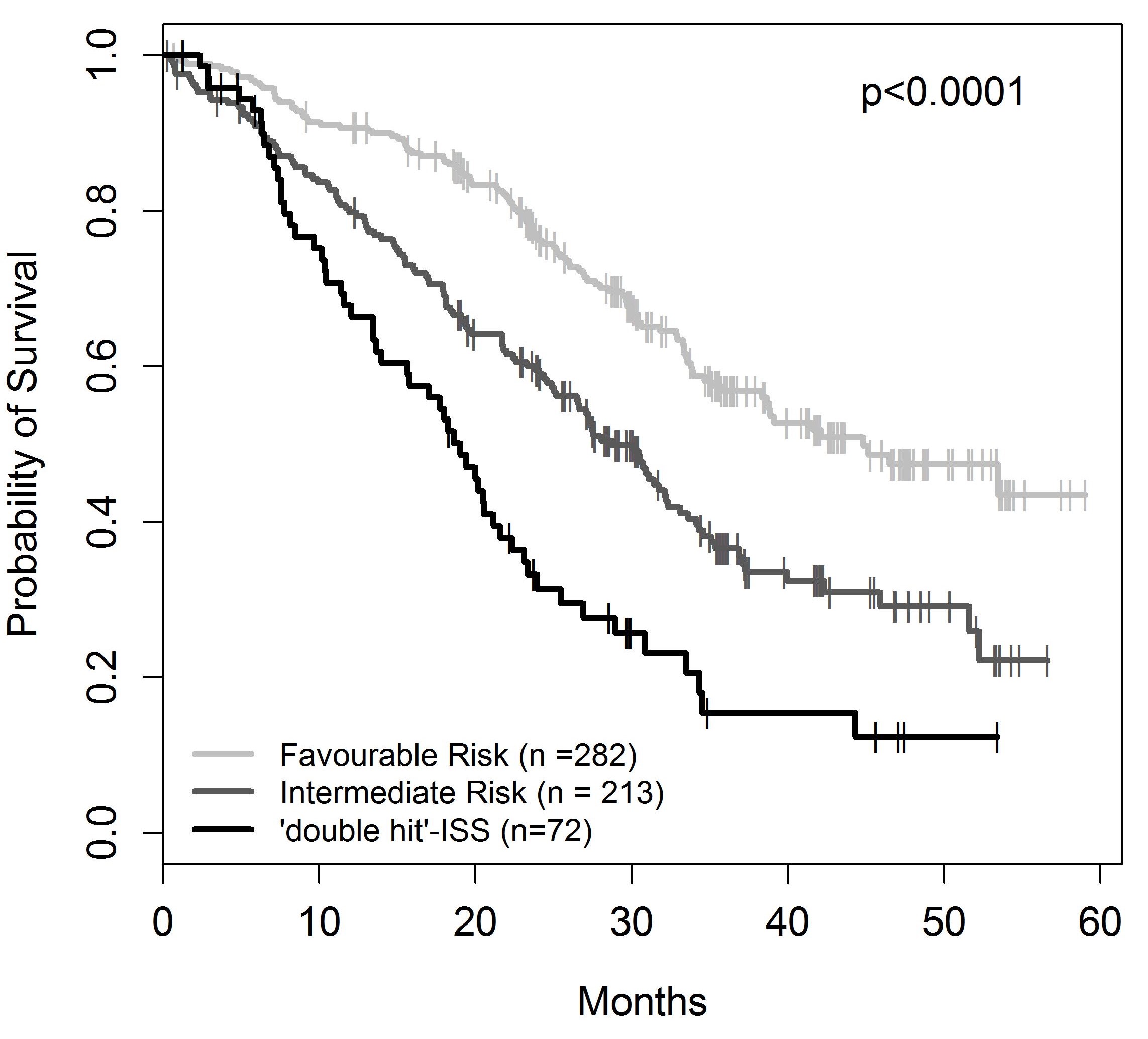 | 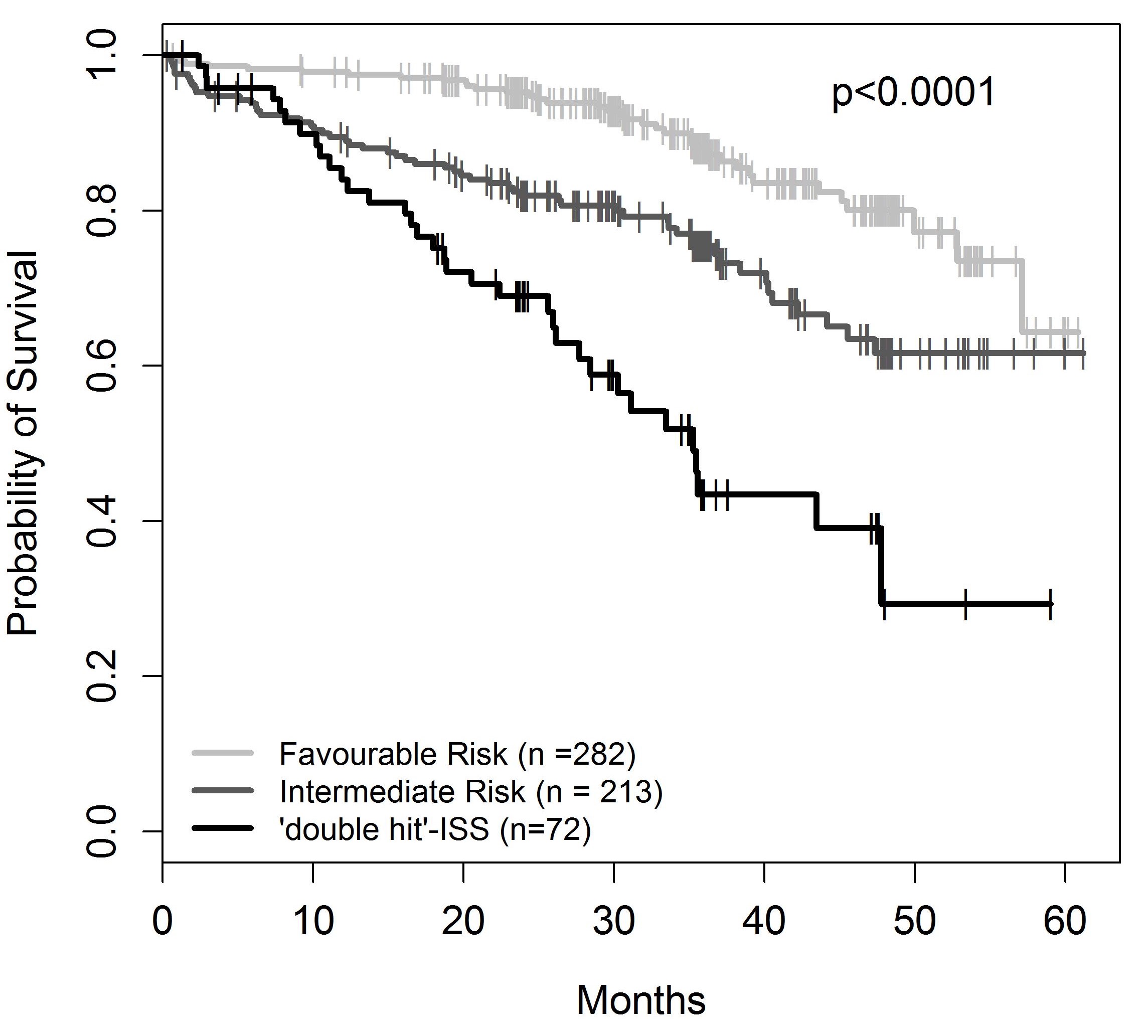 |

**Supplementary Figure 4: Genetic risk markers and survival**

Kaplan-Meier curves and log-rank p-values for 511 intensively treated MRC Myeloma IX patients in the context of presence of absence of recurrent genetic aberrations

1. Adverse Translocation
2. t(4;14)
3. t(14;16)
4. t(14;20)
5. del(17p)
6. gain(1q)
7. ‘double-hit’
8. ‘double-hit’-ISS

|  | PFS | OS |  | PFS | OS |
| --- | --- | --- | --- | --- | --- |
| a | 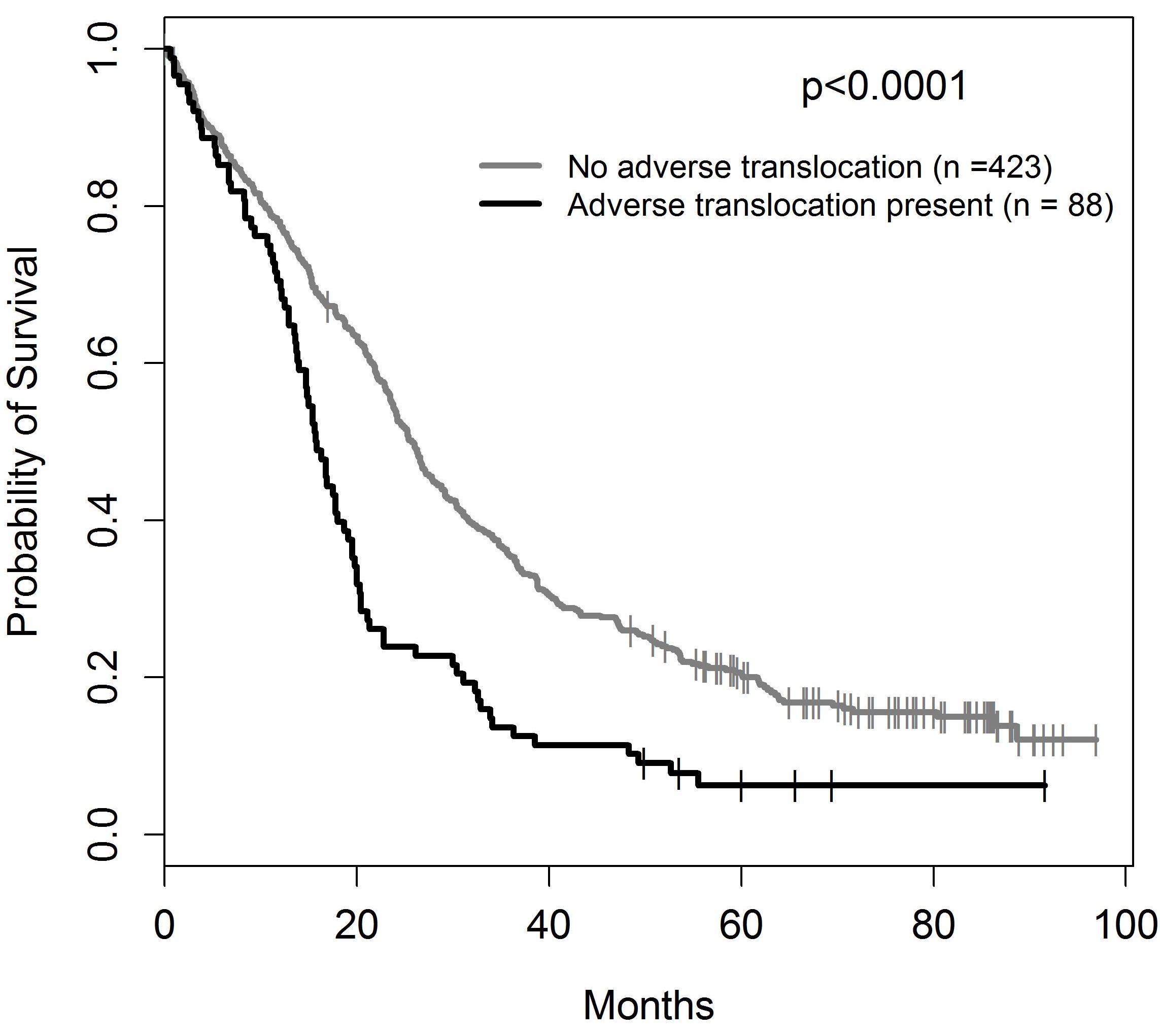 | 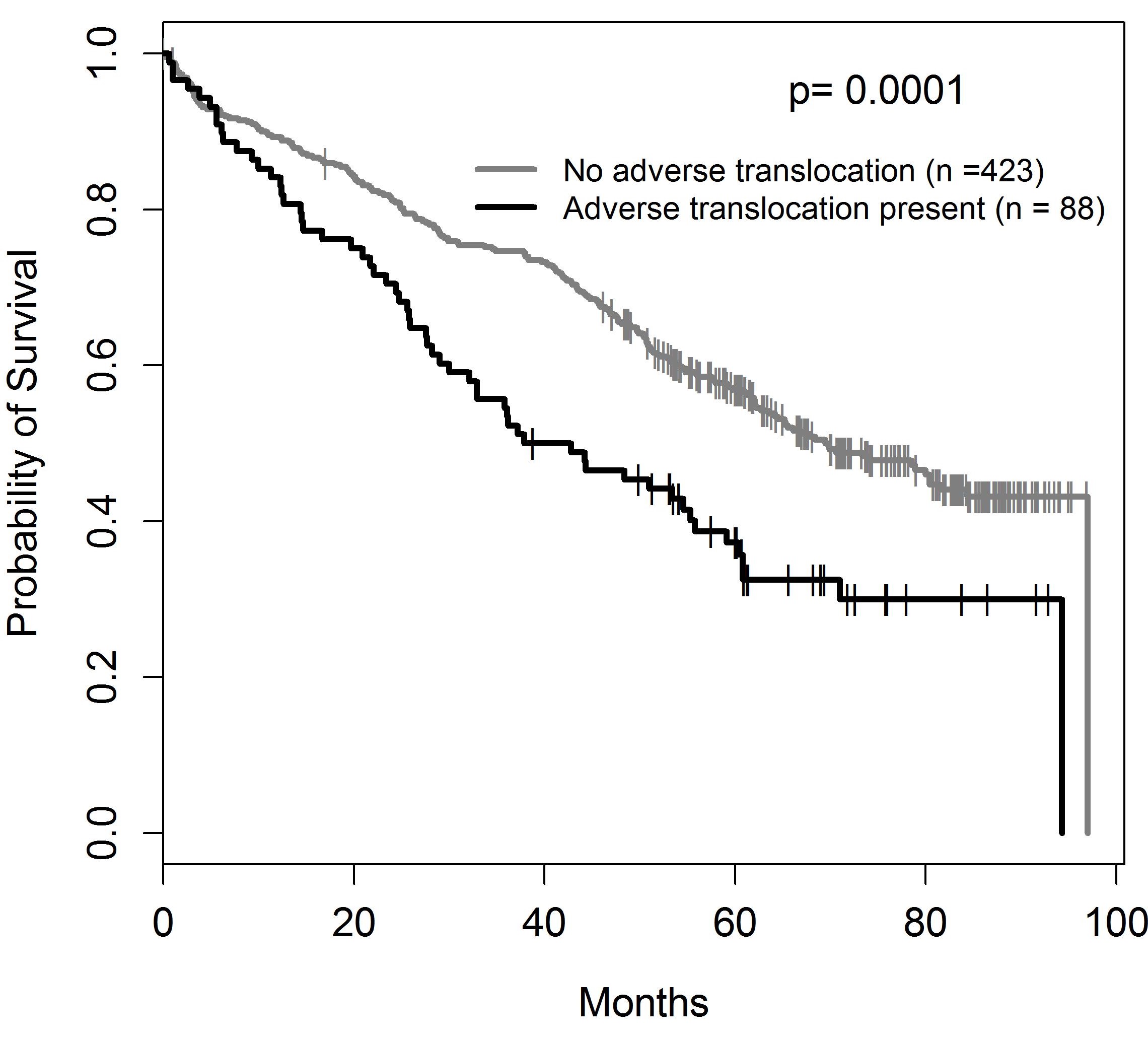 | e | 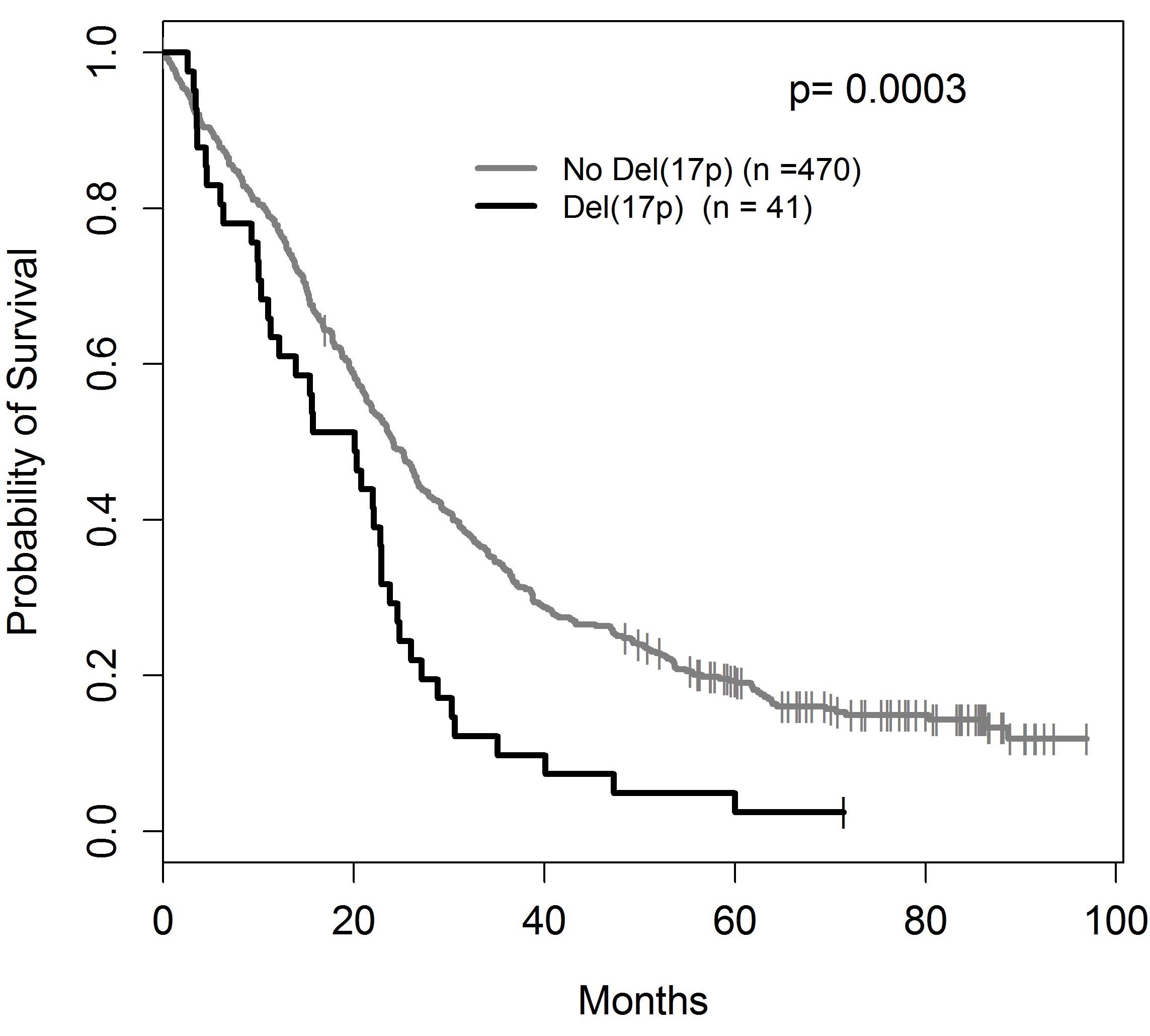 | 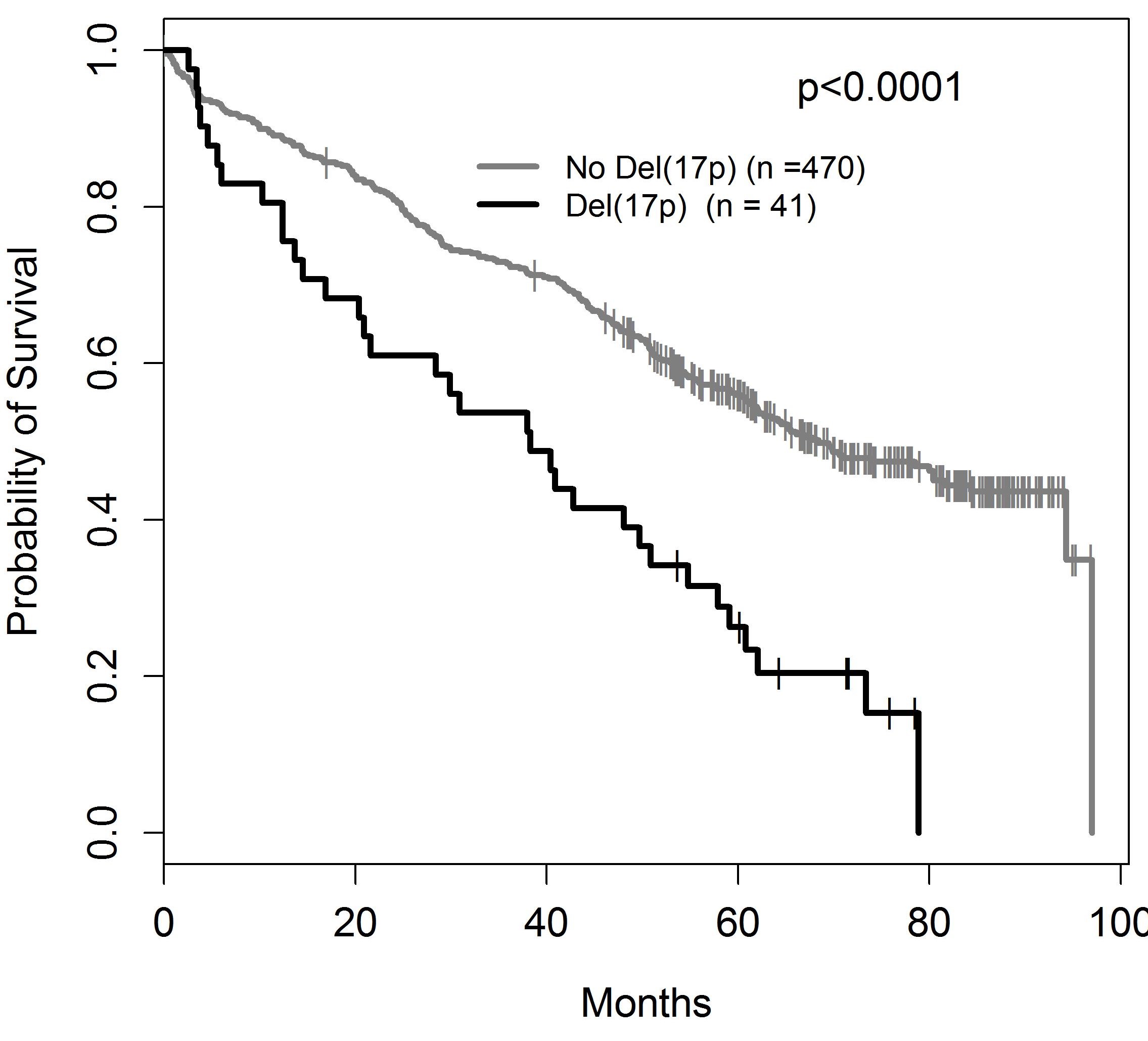 |
| b | 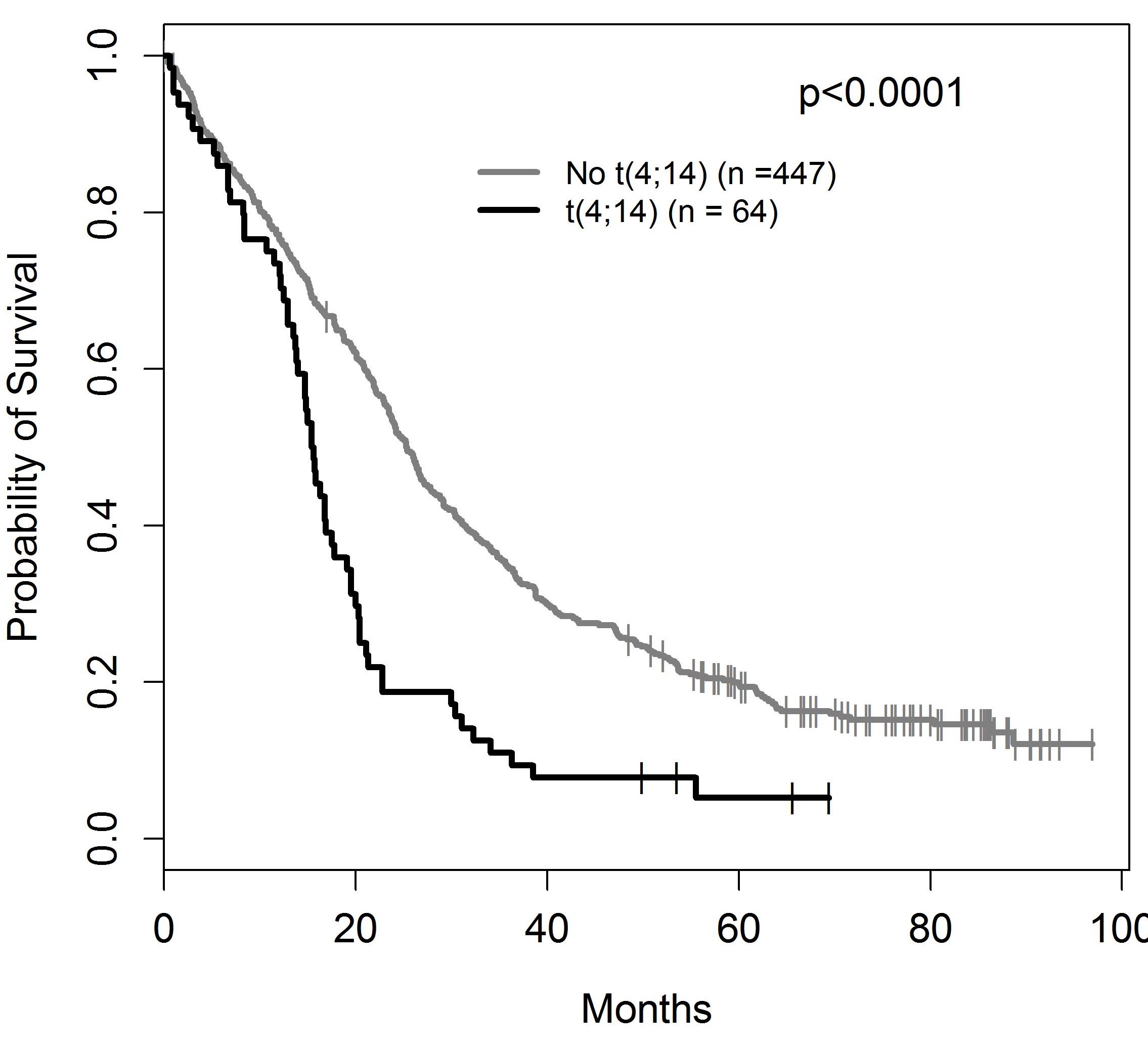 | 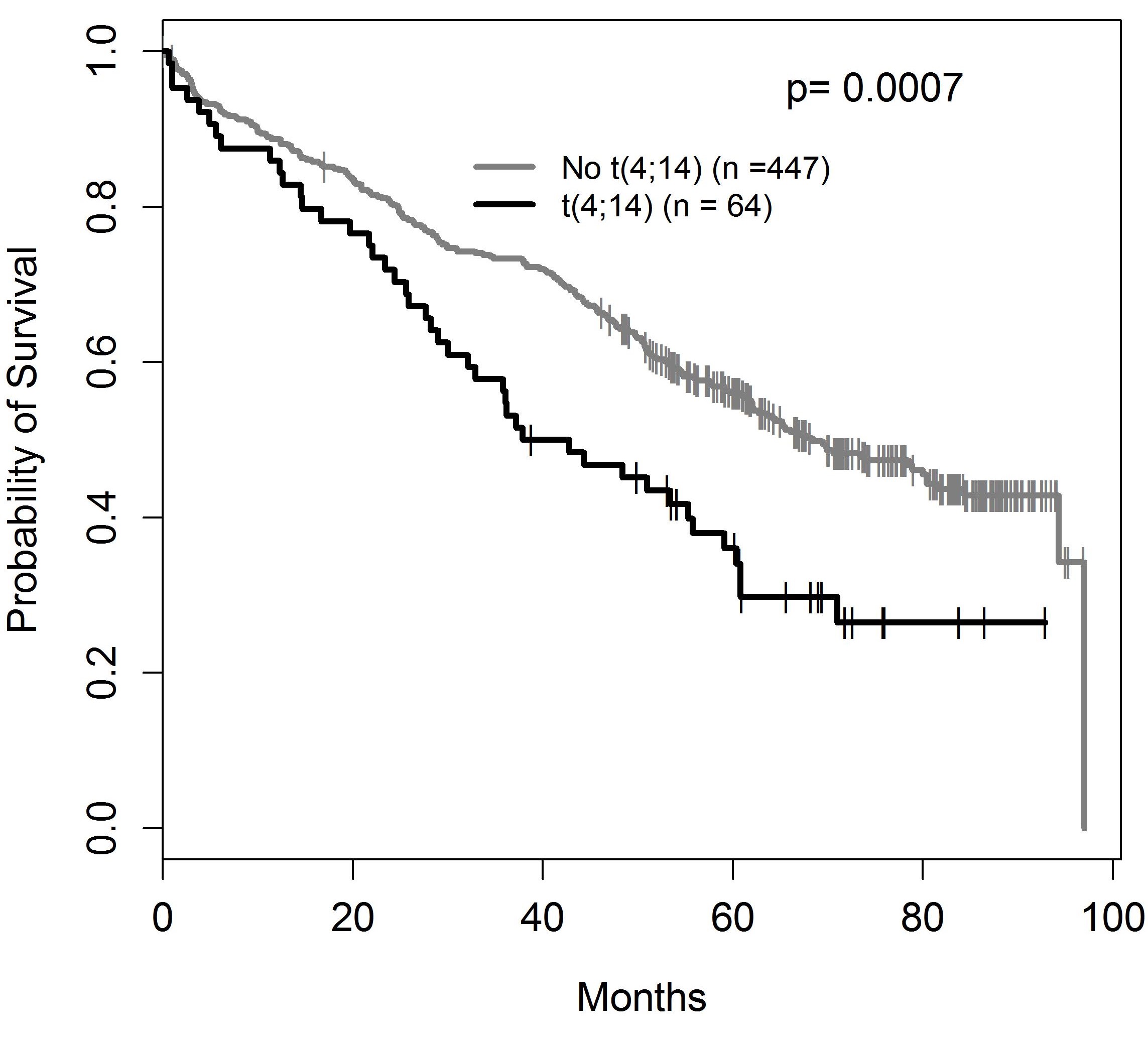 | f | 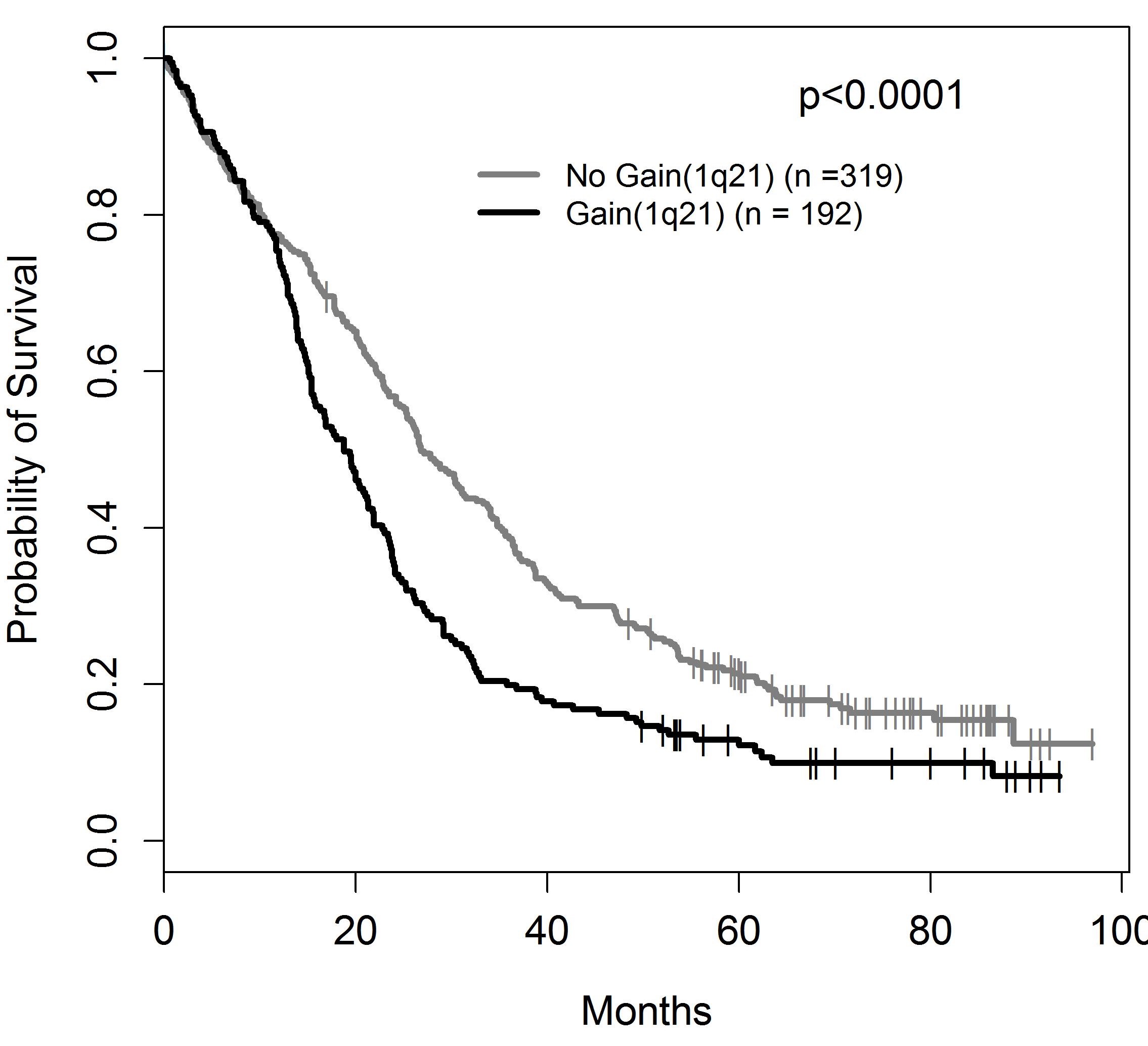 | 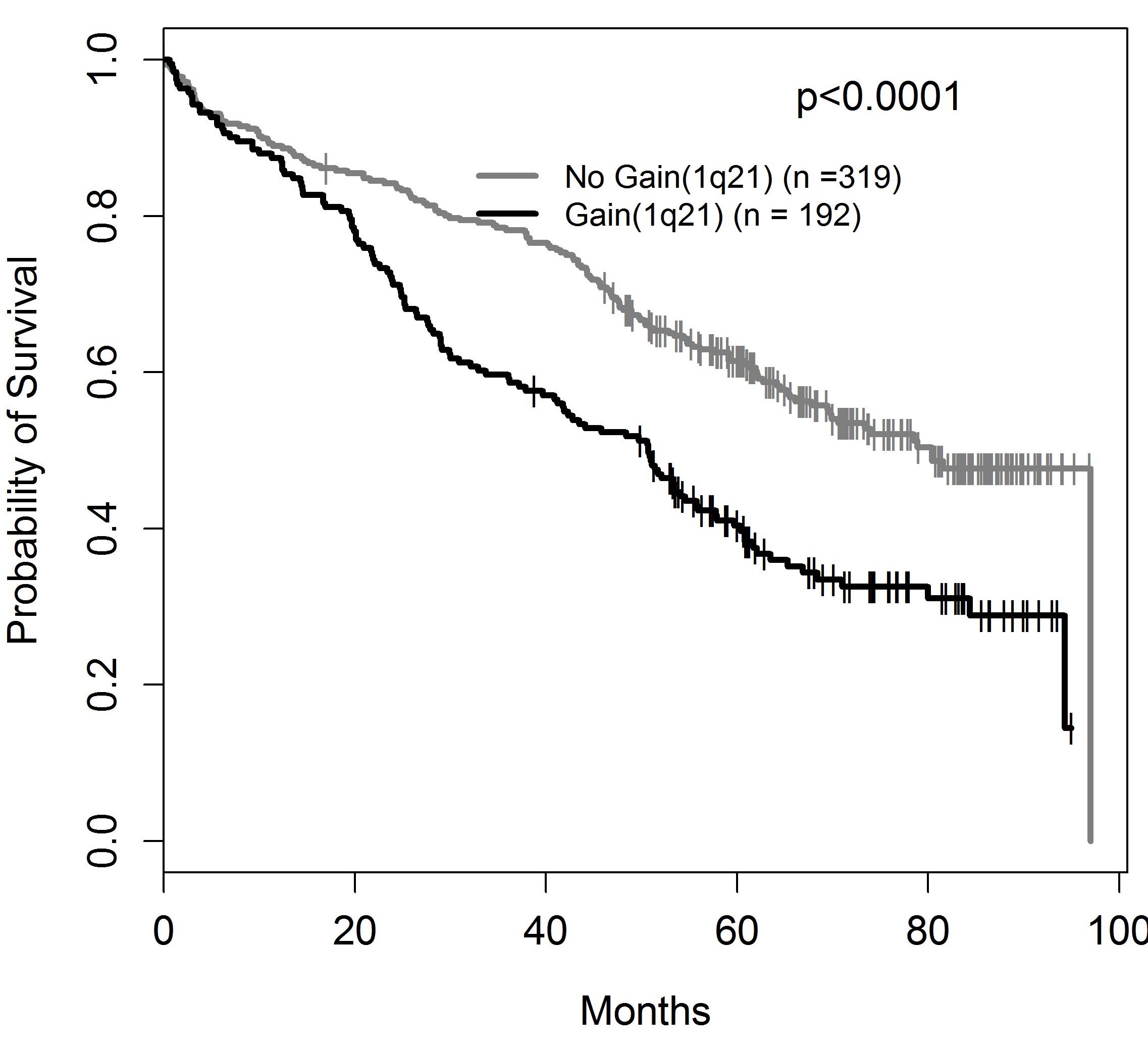 |
| c | 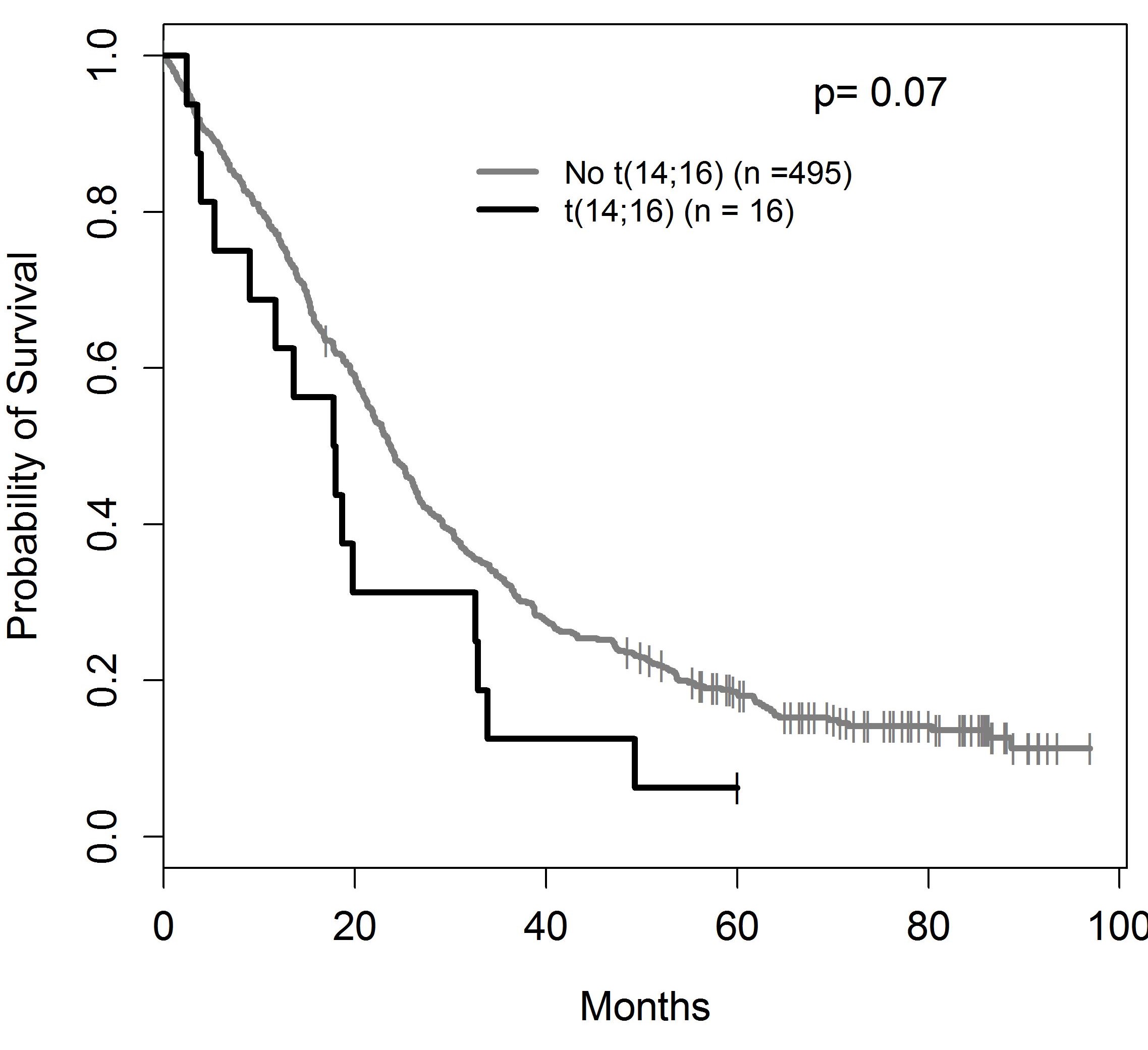 | 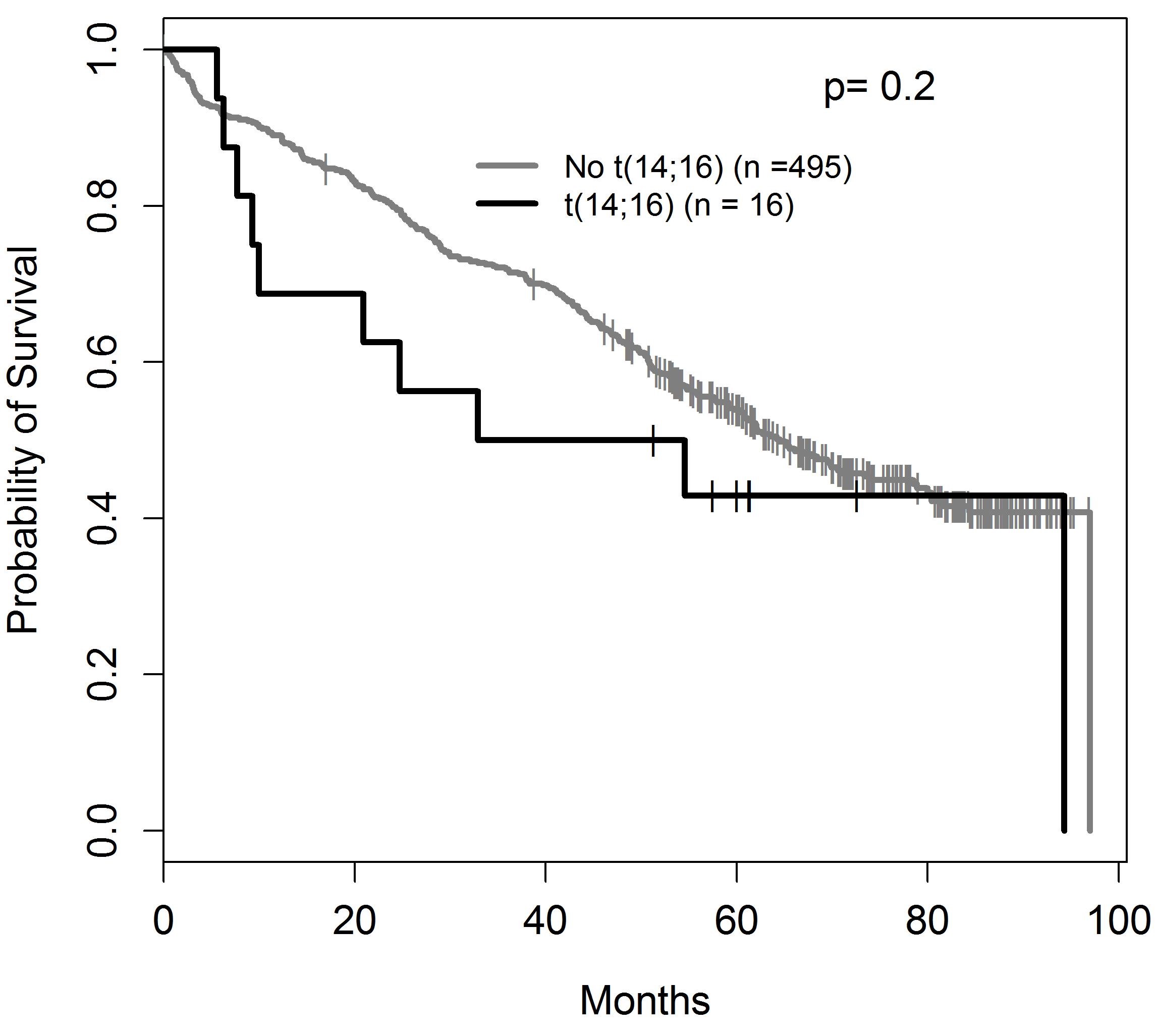 | g | 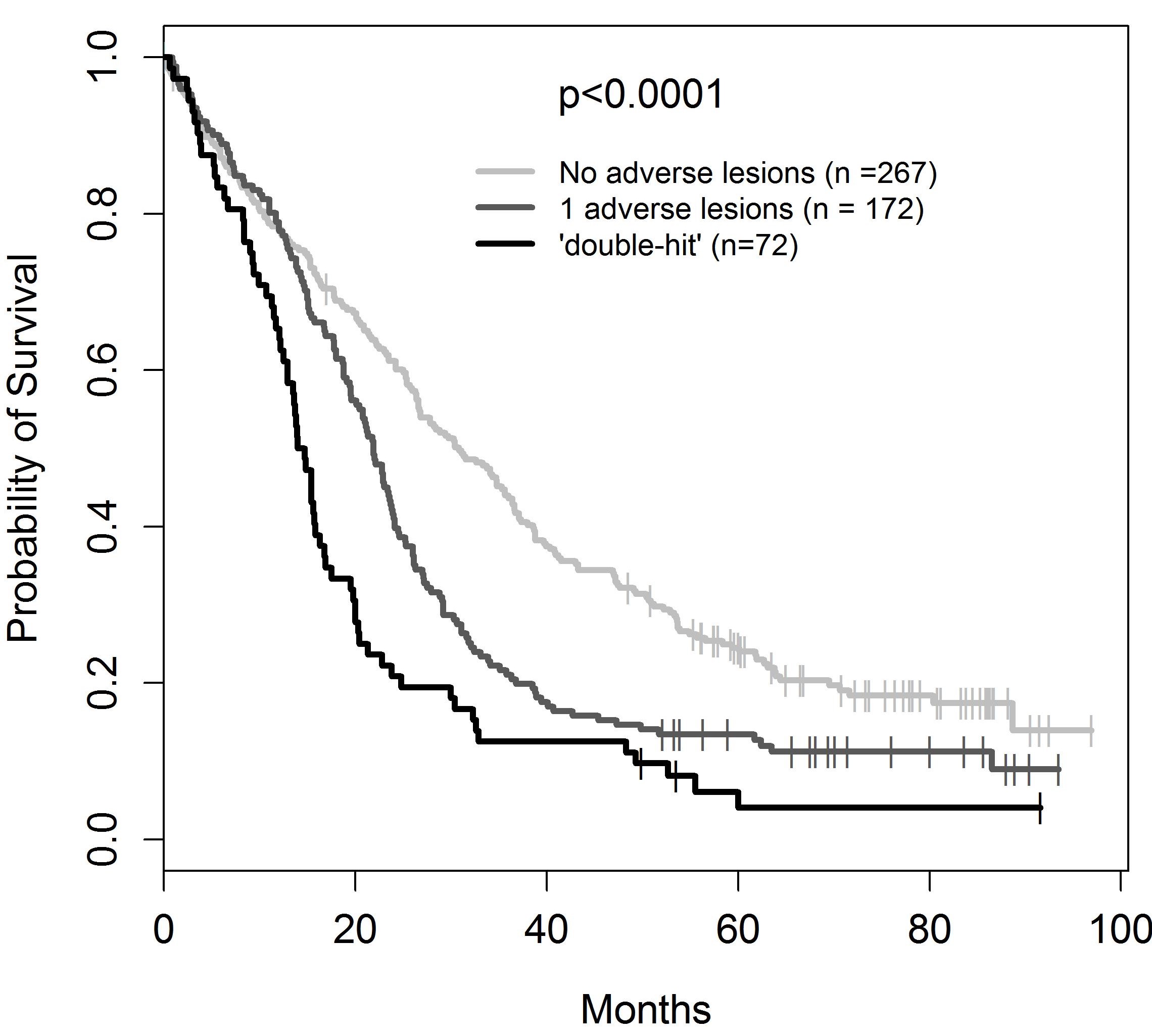 | 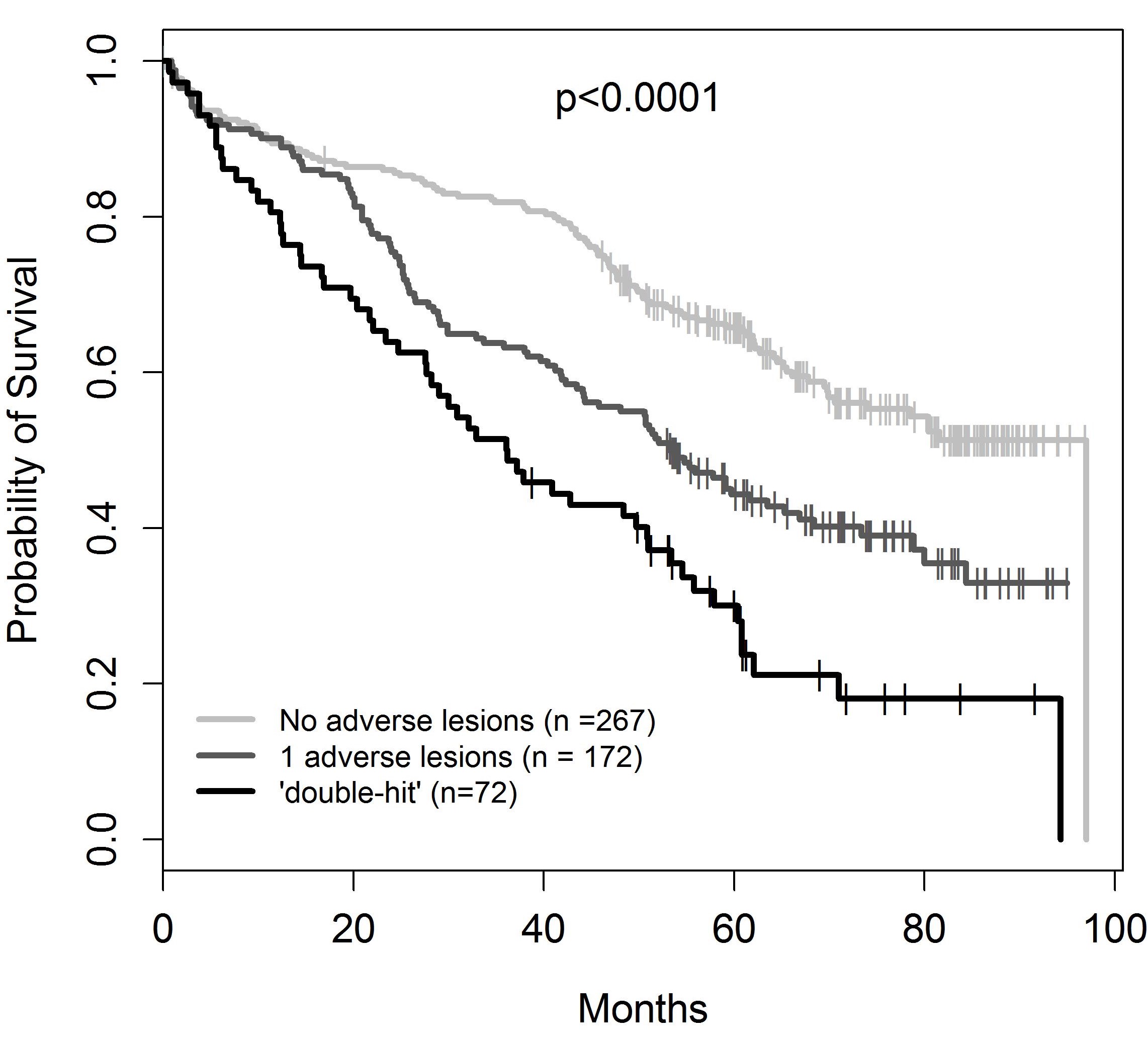 |
| d | 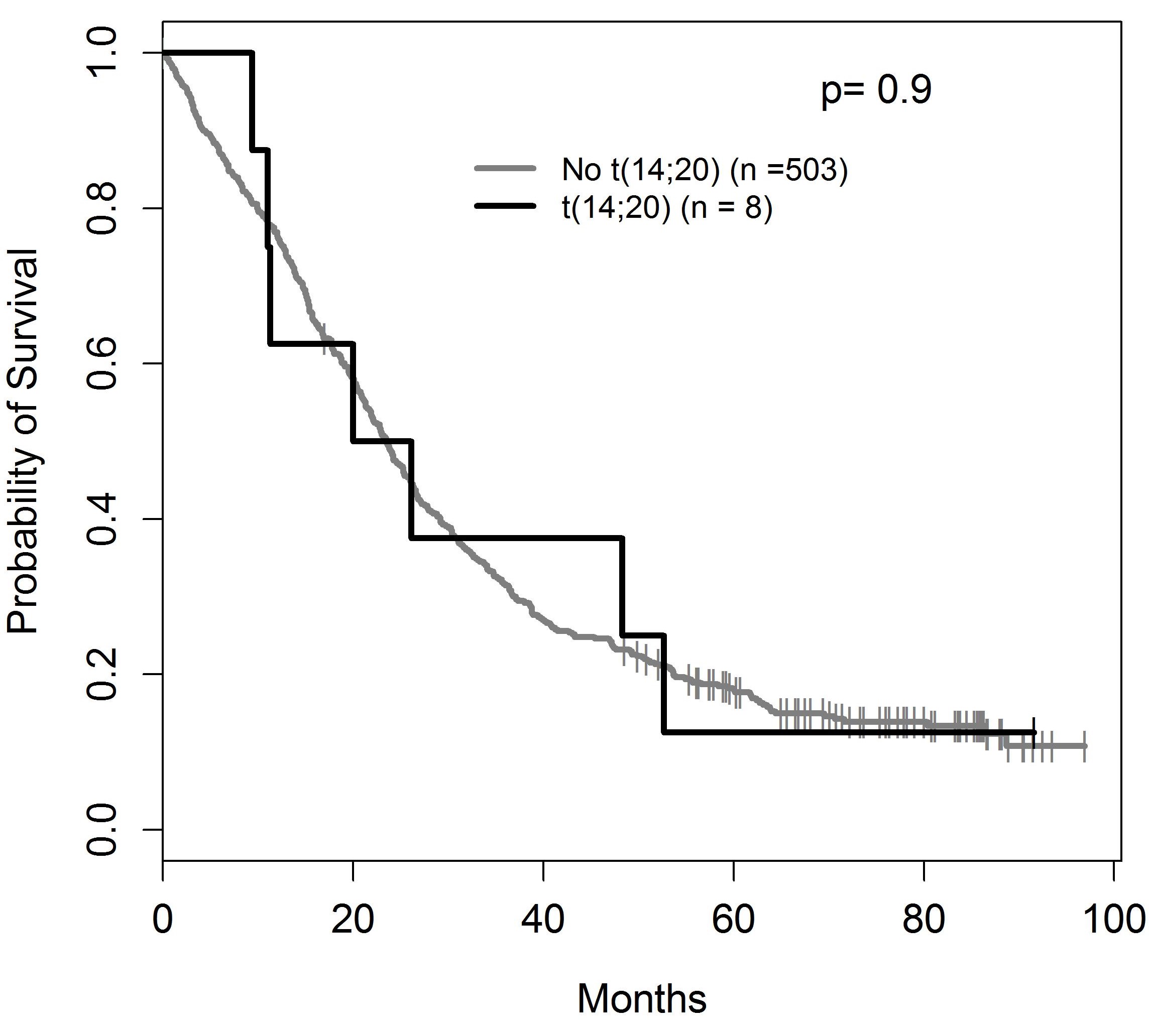 | 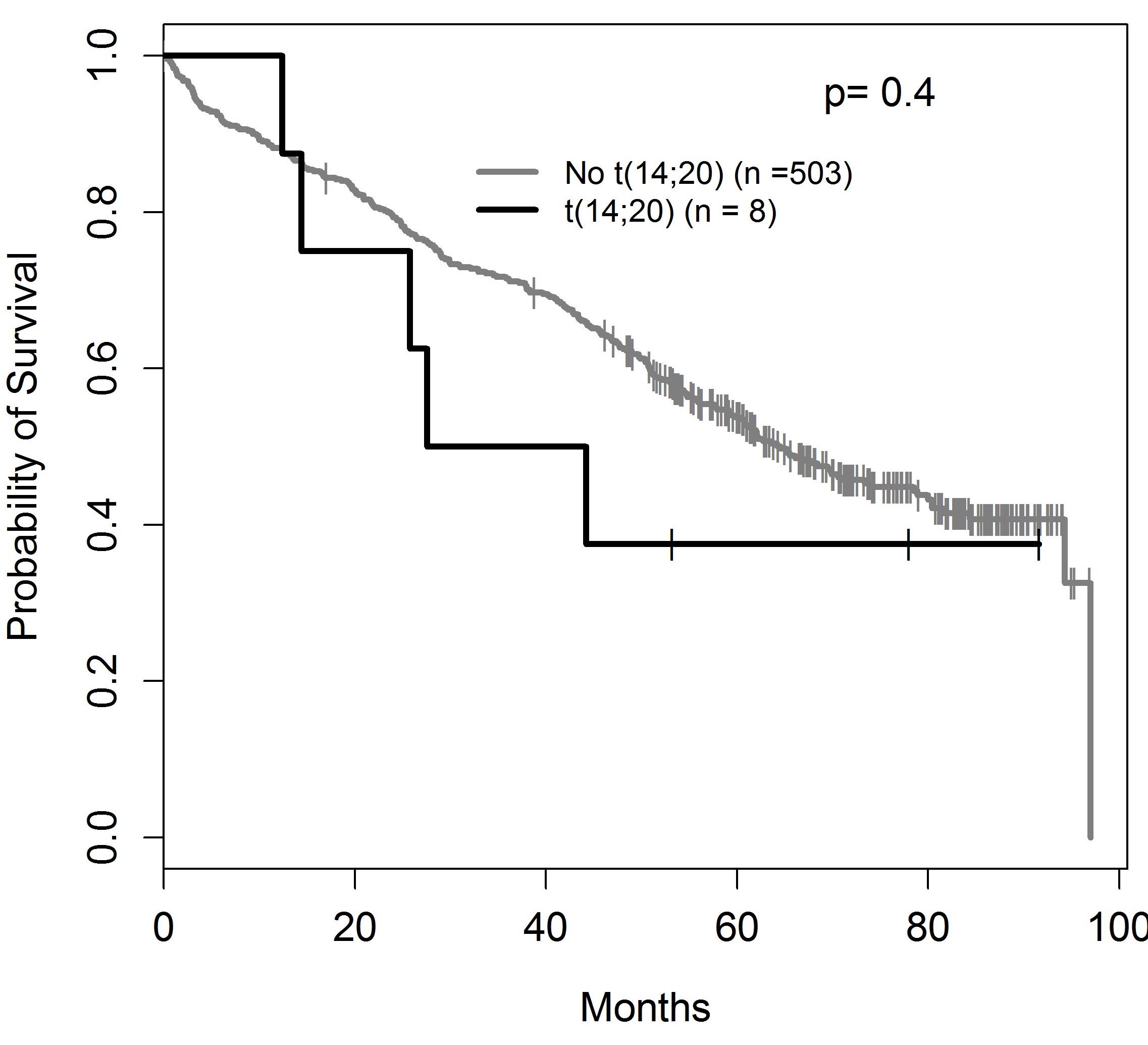 | h | 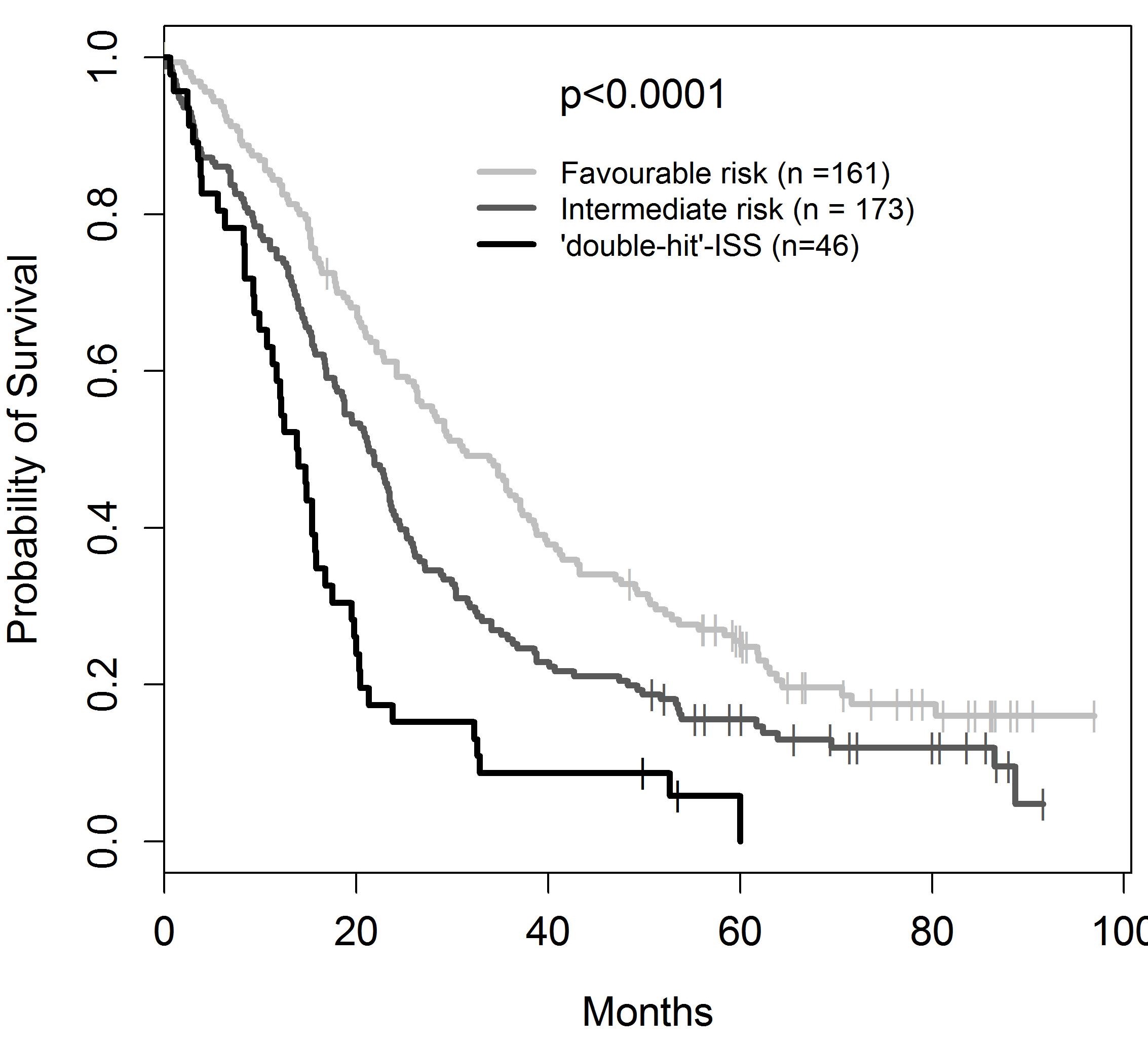 | 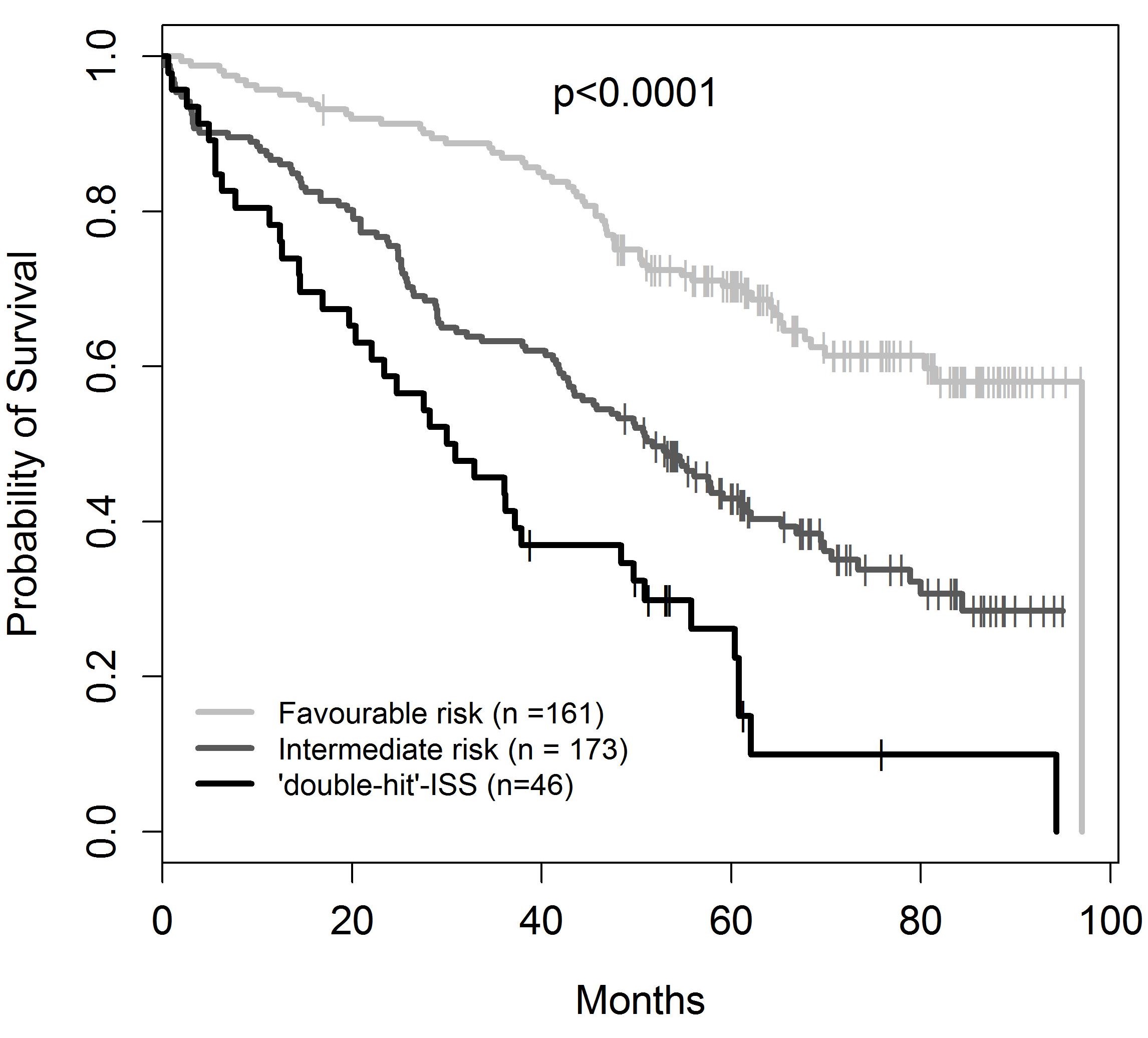 |

**Supplementary Figure 5: Genetic risk markers and survival**

Kaplan-Meier curves and log-rank p-values for 1036 MRC Myeloma XI patients with 0,1,2 or 3 adverse genetic lesions.

| **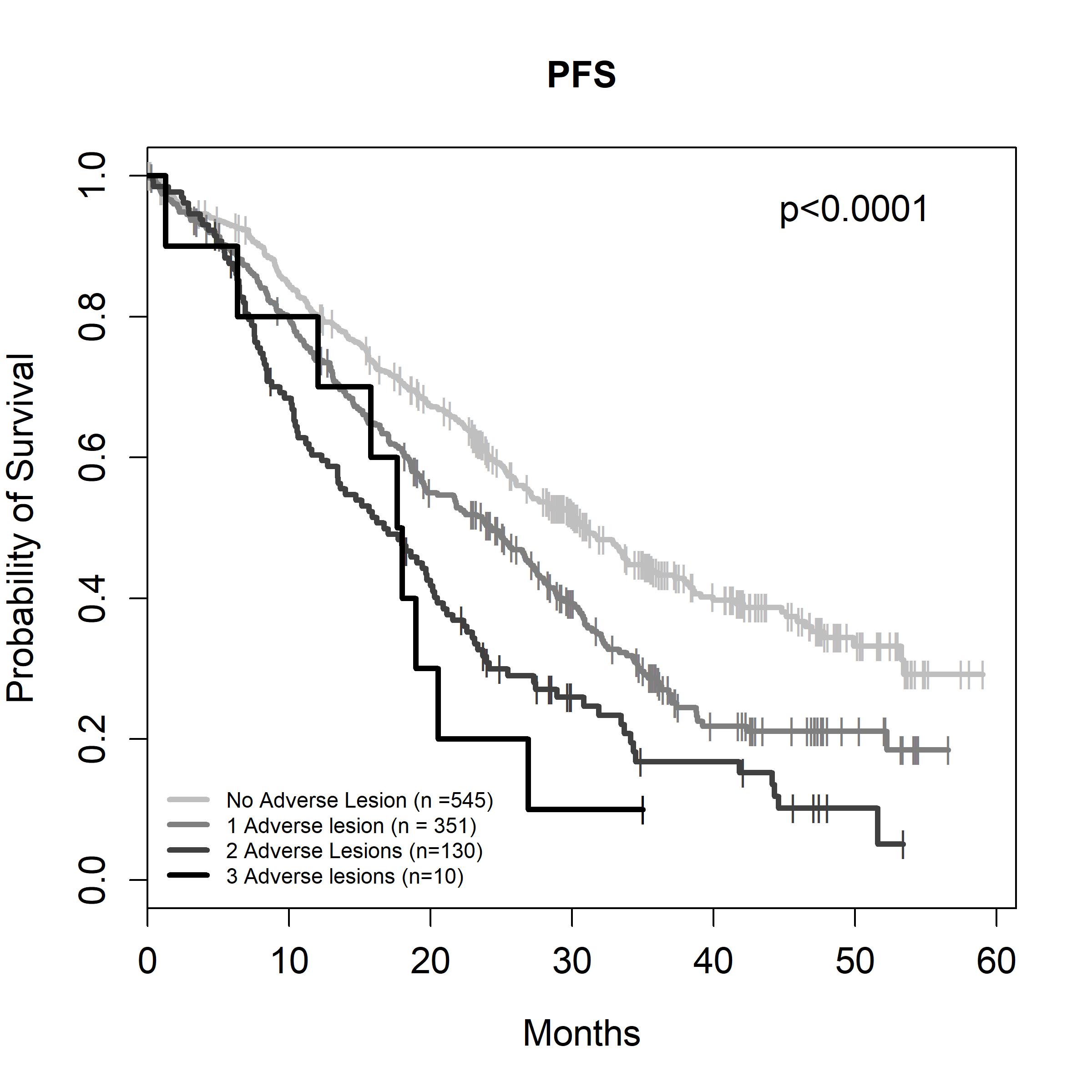** | **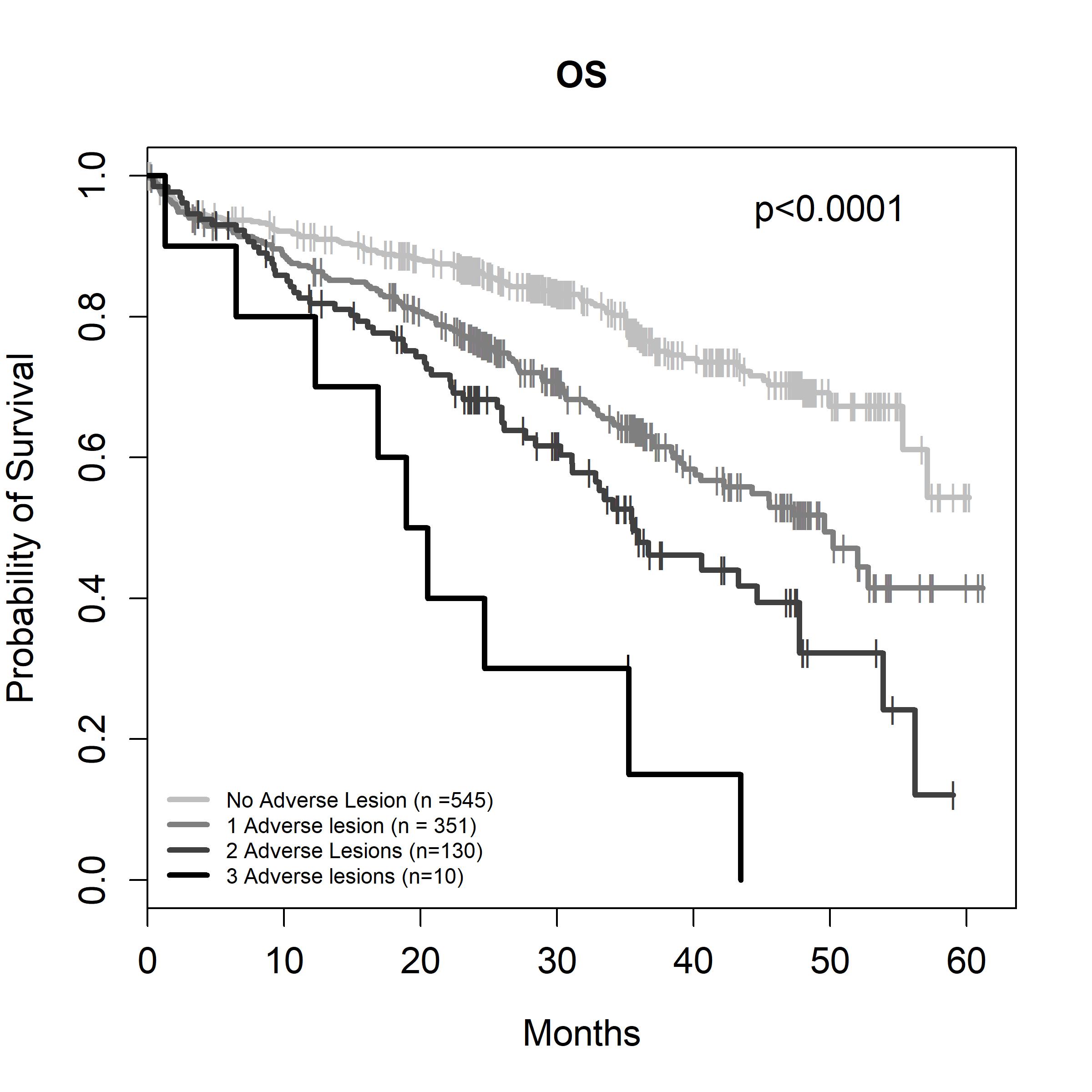** |
| --- | --- |

**Supplementary Figure 6: Frequency of CNA in HRD vs. Non-HRD MM in Myeloma XI**

**Supplementary Figure 7: CCND expression in HRD sub-groups**

Affymetrix GeneChip Human Genome U133 Plus 2.0, GeneChip Mapping 500K Array data (GSE15695) and FISH data from the Myeloma IX trial were integrated and analysed for (a.) CCND1 and (b.) CCND2 expression levels in gain(1q)-HRD and gain(11q)-HRD sub-groups.

1. b.

| 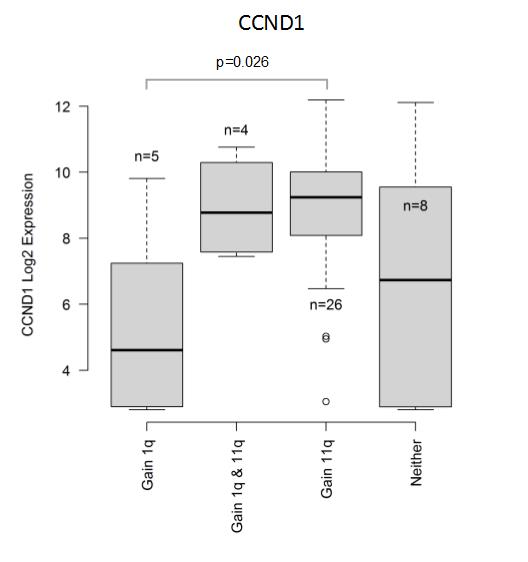 | 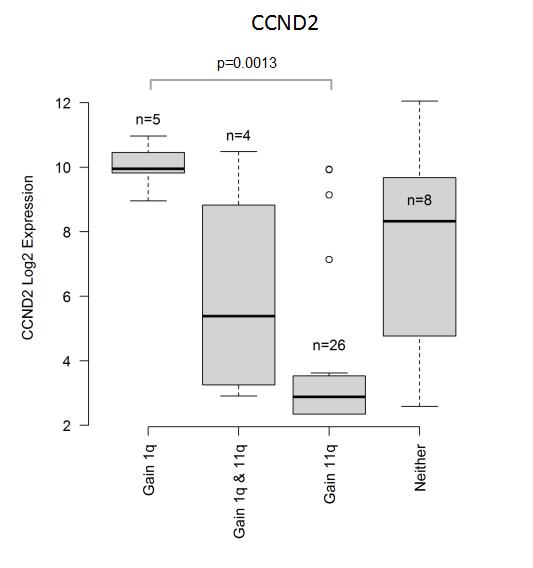 |
| --- | --- |

**Supplementary Figure 8: CNA and survival in HRD MM**

Kaplan-Meier curves and log-rank p-values for sub-groups of 488 Myeloma XI HRD MM characterised by presence of absence of individual risk markers.

a.+b. del(1p32)

c.+d. gain(1q21)

e.+f. del(17p)

|  |  |  |  |
| --- | --- | --- | --- |
| a. | 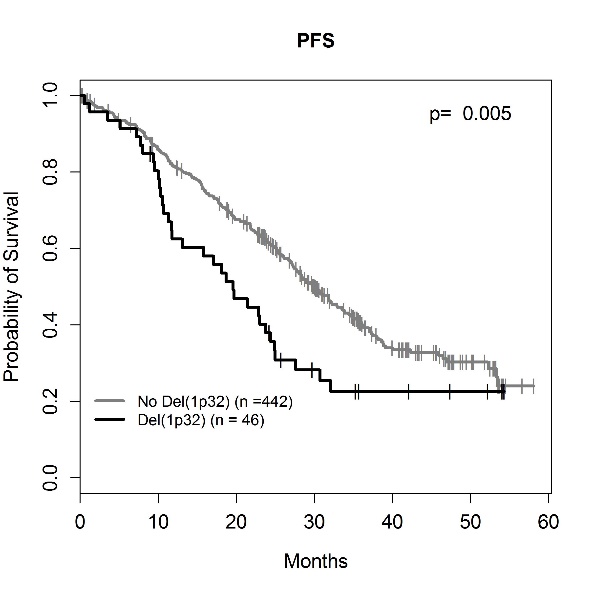 | b. | 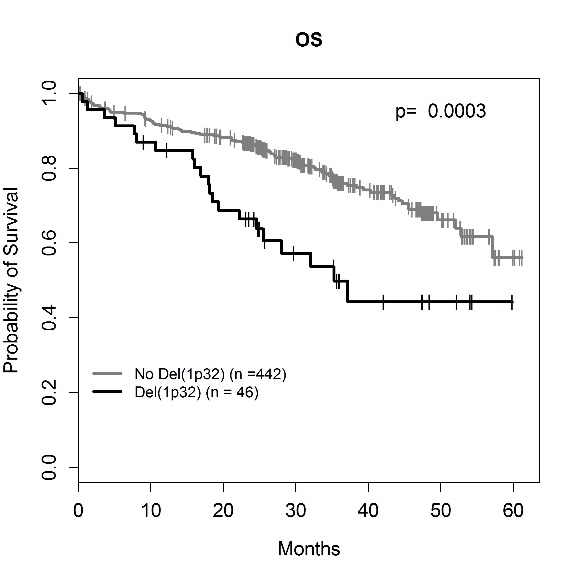 |
| c. | 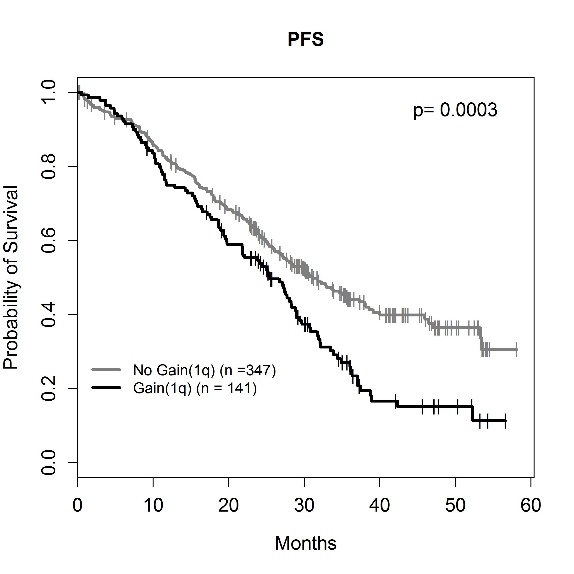 | d. | 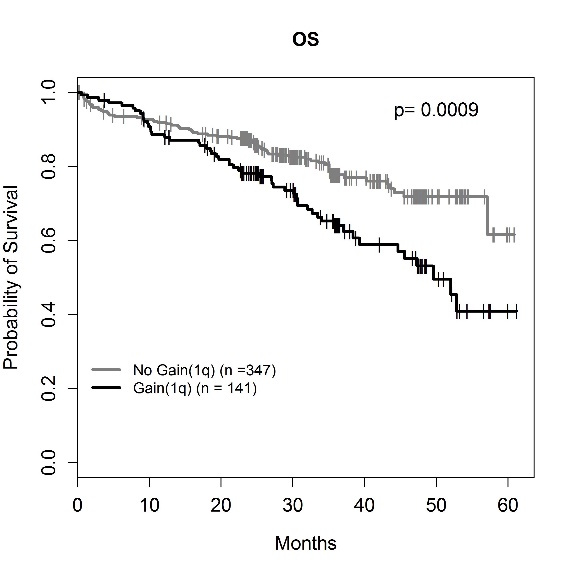 |
| e. | 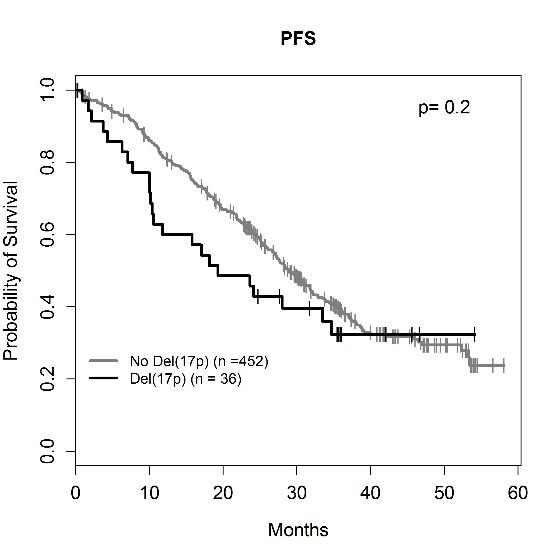 | f. | 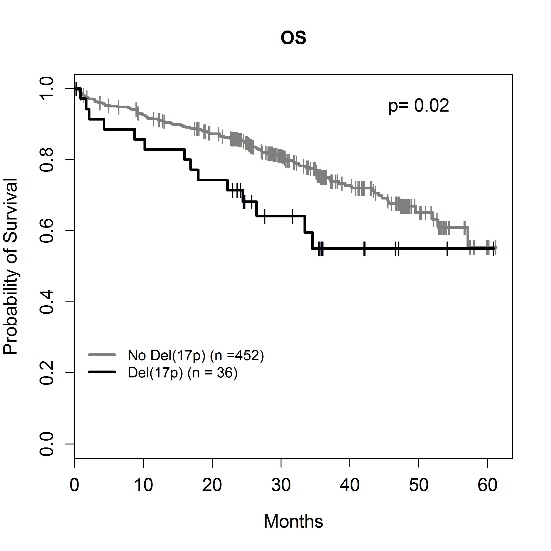 |

**Supplementary Figure 9: Adverse lesions and survival in HRD MM**

Kaplan-Meier curves for sub-groups of 488 Myeloma XI HRD MM characterised by presence of absence of any of genetic risk markers del(1p32), gain(1q21) or del(17p).

|  | **PFS** |  | **OS** |
| --- | --- | --- | --- |
|  | 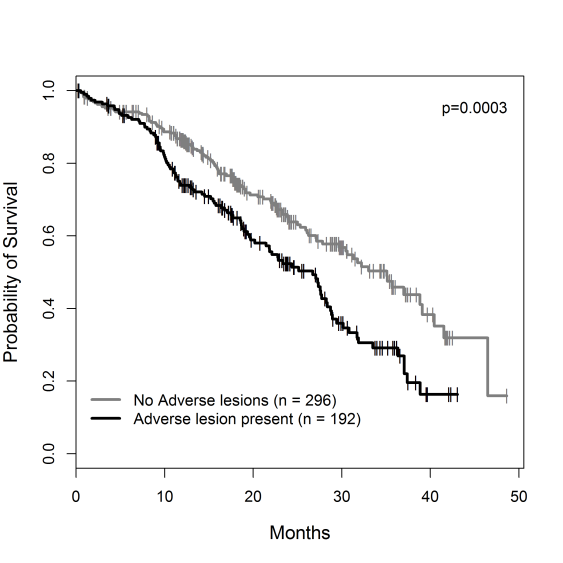 |  | 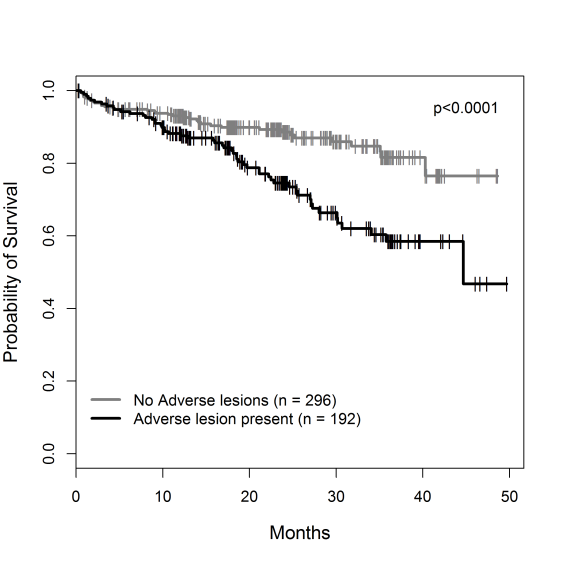 |
